# Testing macroevolutionary predictions of the Grant‐Stebbins model in the origin of *Aeschynanthus acuminatus*

**DOI:** 10.1111/nph.70871

**Published:** 2026-01-27

**Authors:** Jing‐Yi Lu, Yao‐Wu Xing, Hong Truong Luu, Richard H. Ree

**Affiliations:** ^1^ Committee on Evolutionary Biology University of Chicago Chicago IL 60637 USA; ^2^ Department of Collections, Conservation, and Research Field Museum Chicago IL 60605 USA; ^3^ State Key Laboratory of Plant Diversity and Specialty Crops Xishuangbanna Tropical Botanical Garden, Chinese Academy of Sciences Mengla Yunnan 666303 China; ^4^ Institute of Advanced Technology Vietnam Academy of Science and Technology Ho Chi Minh City 700000 Vietnam; ^5^ Southern Institute of Ecology Green Development Solution Services Company Limited Ho Chi Minh City 700000 Vietnam

**Keywords:** *Aeschynanthus*, generalization, Grant‐Stebbins model, ornithophily, pollinator shift, range dynamics, speciation

## Abstract

- The Grant‐Stebbins model predicts that a plant species encountering different pollinators across its range may undergo local adaptation and, subsequently, ecological speciation. We tested whether this could explain the origin of *Aeschynanthus acuminatus* (Gesneriaceae), a species phylogenetically derived from sunbird specialist ancestors. *A. acuminatus* is widespread throughout mainland E Asia but also occurs in Taiwan, beyond the range of sunbirds, where it is pollinated by generalist passerines.
- We hypothesized that *A. acuminatus* originated from an ancestral lineage that colonized Taiwan, rapidly adapted to its novel pollinator fauna, and secondarily spread to the mainland. We tested among evolutionary scenarios by integrating studies of phylogeography, pollination, and floral morphology.
- Phylogeographic analysis of genome‐wide SNPs revealed a mainland origin. Pollinator observations showed varied visitation by both sunbirds and generalist passerines across mainland Asia. The origin of *A. acuminatus* likely involved a pollinator niche expansion to include generalist passerines, an ecological shift that enabled its subsequent range expansion.
- Hypothetical pollinator‐mediated fitness models suggest that the derived floral morphology of *A. acuminatus* represents an adaptive optimum for generalist passerine pollination rather than an intermediate phenotype. Our research illustrates how the evolution of pollinator niches can influence the origin and range dynamics of plant species.

## Introduction

A compelling reason to study plant‐pollinator interactions is their potential to bridge the gaps between microevolution and macroevolution. A key question is how geographic variation in pollinator availability may drive adaptation and speciation in plants (Aigner, 2005; Thompson, 2005; Johnson, 2006). The Grant‐Stebbins model of pollinator‐mediated speciation (Johnson, 2006) posits that: (1) pollinators are distributed unevenly across the landscape, forming a geographic mosaic of local pollinator communities (Grant & Grant, 1965); and (2) floral phenotypes are selected by pollinator effectiveness (‘the most effective pollinator principle’, Stebbins, 1970). The model predicts that in geographically isolated populations with different pollinator communities, floral traits will become adapted to the locally most effective pollinators, and this morphological divergence will then lead to reproductive isolation, population genetic divergence, and eventually speciation. The process highlights the dual role of floral traits in the key components of ecological speciation: morphological divergence and reproductive isolation (Van der Niet *et al*., 2014a).

The Grant‐Stebbins model is thus a useful framework for investigating the roles of local adaptation and pollinator switching in the origin of species that are distributed across a geographic mosaic of pollinators. Previous research has mainly focused on detecting covariation between the geographic distributions of plants and their animal pollinators to demonstrate coevolution (e.g., Anderson & Johnson, 2008; Martén‐Rodríguez *et al*., 2011). More recently, infraspecific studies have examined whether floral traits covary geographically with local pollinators, especially in generalized pollination systems (Torres‐Vanegas *et al*., 2024; Moir *et al*., 2025), showing that pollination ecotypes can indeed arise through local adaptation to the most effective pollinators. However, to understand how such ecotypes can lead to pollinator‐mediated speciation over a longer time frame, integration of phylogeographic inferences with ecological data is needed (see Van der Niet *et al*., 2014b). Ancestral state estimation of pollinators and geographic ranges on population phylogenies can help determine whether or not the origin of an extant species or ecotype coincided with a change in pollinator availability.

An opportunity to test predictions of the Grant‐Stebbins model presents itself in the SE Asian genus *Aeschynanthus* (Gesneriaceae). All but one of its 160 species exhibit a typical bird pollination syndrome, with long reddish corolla tubes and copious dilute nectar (Burtt & Woods, 1975; Freeman *et al*., 1991; Middleton, 2007), and their primary pollinators are believed to be specialized nectar‐feeding sunbirds (Nectariniidae; Middleton, 2016). As expected, the geographic ranges of these species are contained within the range of sunbirds.

**Fig. 1:**
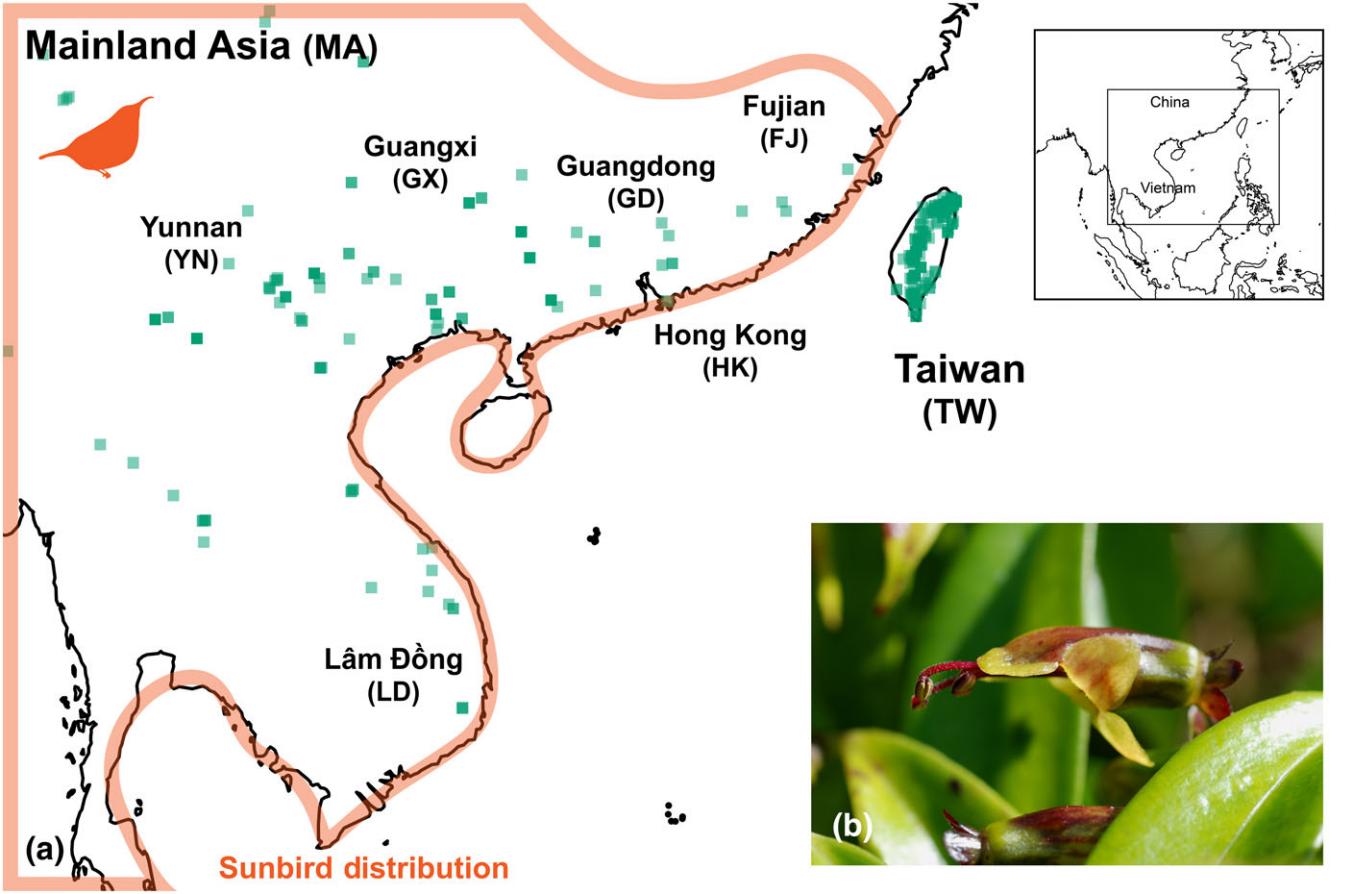
Geographic context and study species. (a) Geographic ranges of *Aeschynanthus acuminatus* and sunbirds (Nectariniidae). The inset map (top right) shows the regional context in Asia, with the black box indicating the extent of the main panel. Green squares indicate occurrence records of *A. acuminatus*. The vermilion line outlines the geographic range of sunbirds. Taiwan is a unique mismatch in the ranges of *A. acuminatus* and sunbirds. Geographic regions sampled in this study are labeled. (b) Lateral view of the flower of *A. acuminatus*.

A conspicuous exception is *Aeschynanthus acuminatus*, a species that is widespread across mainland East Asia, within the range of sunbirds, but also occurs in Taiwan, where sunbirds are absent (Fig. 1). In Taiwan, studies of pollen transfer and female fitness showed the species to be exclusively pollinated by a group of uncommon avian pollinators, generalist passerine birds (Chen *et al*., 2019). Corolla tubes of *A. acuminatus* are wider and shorter than the typical sunbird‐pollinated form of other congeneric species, facilitating nectar access by the usually thicker and shorter beaks of generalist passerines. Phylogenetically, the species is nested well within *Aeschynanthus* (Denduangboripant *et al*., 2001), so it is likely that the characteristic floral traits of *A. acuminatus*, and its pollination by generalist birds in Taiwan, represent traits derived from a sunbird‐pollinated ancestor.

**Fig. 2:**
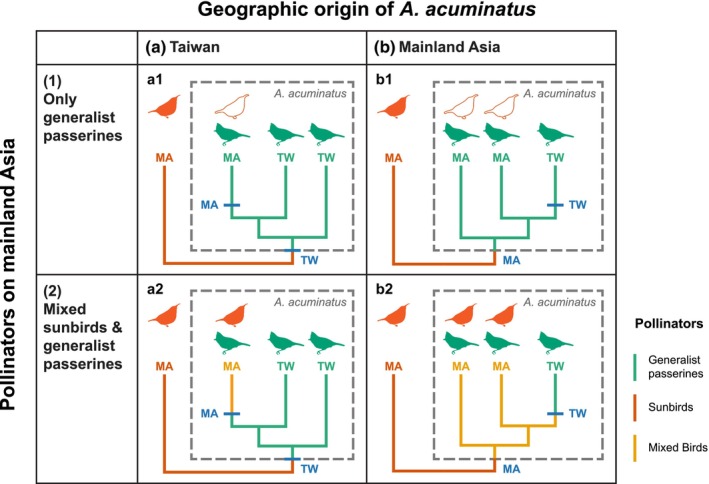
Phylogenetic scenarios of geographic range evolution and pollinator shifts in the origin of *Aeschynanthus acuminatus*. Tips of phylogenetic branches are labeled by geographic region: Taiwan (TW) and mainland Asia (MA). The vermilion branch represents the sunbird‐specialist outgroup. Ingroup branches are colored by their pollinators (green, generalist passerines; orange, mixed generalist passerines and sunbirds). Bird silhouettes indicate pollinators (green, generalist passerines; vermilion, sunbirds). Hollow vermilion silhouettes represent sunbirds not visiting flowers when they are present. Dispersal events are indicated by bars on phylogenetic branches, labeled by the colonized area. Columns correspond to hypotheses of geographic origin: Taiwan (a) vs mainland Asia (b). Rows correspond to hypothesized pollinators of *A. acuminatus* in mainland Asia: generalist birds only (1) vs generalist birds and sunbirds (2). These yield four possible historical scenarios. a1: Origin in Taiwan via a shift to exclusive generalist passerine pollination that is maintained on the mainland. a2: As a1, with pollination by both bird types on the mainland. b1: Origin on the mainland via a shift to exclusive generalist passerine pollination. b2: Origin on the mainland via a shift to pollination by both bird types.

What roles did range expansion and pollinator switching play in the origin of *A. acuminatus*? An obvious prediction of the Grant‐Stebbins model is that a sunbird‐pollinated ancestral species colonized Taiwan and rapidly evolved generalist passerine pollination (Scenario a, Fig. 2a). In this scenario, *A. acuminatus* originated in Taiwan by adapting to its local pollinator fauna (Johnson, 2006), and the modern presence of the species in mainland Asia is due to recolonization from Taiwan. Phylogenetically, this predicts that the mainland populations should be sister to, or derived from, those in Taiwan.

An alternative scenario is that *A. acuminatus* first evolved pollination by generalist passerines on the mainland, an ecological shift that facilitated its subsequent range expansion to Taiwan (Scenario b, Fig. 2b). As this shift would have occurred within the range of sunbirds, it could have involved a complete switch of pollinator niche from sunbirds to generalist passerines, or an expansion, in which some degree of sunbird pollination is retained in mainland populations. This raises an important secondary question: what pollinates *A. acuminatus* on the mainland?

If *A. acuminatus* originated on the mainland in the presence of both sunbirds and generalist passerines, the pollination mode of mainland populations should reflect how the species evolved from its sunbird‐specialist ancestor. Exclusive pollination by generalist passerines would indicate that the origin of *A. acuminatus* coincided with a replacement of sunbird pollinators with generalist passerines (Fig. 2b1). Sympatric pollinator niche partitioning can arise if differential pollinator effectiveness leads to the formation of ecotypes in the ancestral population (Abrahamczyk, 2019). On the other hand, pollination of mainland *A. acuminatus* by both sunbirds and generalist passerines would indicate that the species originated via an expansion of its pollinator niche (Fig. 2b2), which could arise under an unstable pollinator climate as an adaptation to increase reproductive assurance (Waser *et al*., 1996).

In addition to where *A. acuminatus* originated and how it ancestrally evolved generalist‐passerine pollination, the Grant‐Stebbins model predicts that its floral traits in Taiwan reflect local adaptation to generalist passerines. If pollination by sunbirds occurs on the mainland, floral traits should vary accordingly, perhaps driven by selection toward an intermediate phenotype that balances fitness contributions by each bird type.

To investigate these evolutionary scenarios, we conducted studies of phylogeography, pollination, and floral morphology. Our objectives are to determine: (1) whether *A. acuminatus* originated on Taiwan or the mainland; (2) how pollination mode evolved at the origin of the species and during its subsequent range expansion; and (3) if floral traits exhibit evidence of local adaptation by covarying with pollinators across its range.

## Materials and Methods

### Phylogeography

We collected leaf tissues of *A. acuminatus* Wall. ex A.DC. across its geographic range. In total, 172 individuals were sampled from 44 populations, 24 on the mainland and 20 on Taiwan. Guided by previous studies of morphology (Middleton, 2009) and molecular phylogeny (Denduangboripant *et al*., 2001), we also sampled its putatively close relative, *A. bracteatus* Wall. ex A.DC. (17 individuals from 9 populations), particularly in the regions adjacent to the distribution of *A. acuminatus*, as well as other species in the same taxonomic section (*Haplotrichium* Benthem) as *A. acuminatus*, *A. superbus* C.B.Clarke and *A. wardii* Merr., and the more distantly related *A. hookeri* C.B.Clarke and *A. jouyi* D.J.Middleton (list of samples in Supporting Information Table S1). Voucher specimens, 1 to 3 per population, were deposited in the Field Museum Herbarium (F).

We generated genome‐wide SNPs using restriction‐site associated DNA (RAD) sequencing (Davey & Blaxter, 2010) with a single restriction enzyme *ApeKI*, using a genotyping by sequencing protocol (Elshire *et al*., 2011). We first isolated DNA from silica‐dried leaf samples using the Invisorb® Spin Plant Mini Kit (STRATEC Molecular, Birkenfeld, Germany). The library was prepared and sequenced at the University of Wisconsin Biotechnology Center DNA Sequencing Facility on an Illumina NovaSeq (2 × 150‐bp reads).

We assembled raw reads into loci using ipyrad (Eaton & Overcast, 2020). First, we demultiplexed, trimmed, and filtered the raw reads, removing reads < 35 bps long or with 5 or more bps having a Qscore < 33. We then aligned reads to a *de novo* assembled *A. acuminatus* genome (Lu *et al*., in prep) and called single nucleotide polymorphisms (SNPs) with a minimum sequencing depth of 6. We removed loci with > 50% polymorphic sites, more than 5 indels, and all multiallelic SNPs (assuming diploids).

To examine the influence of missing data, we filtered loci based on a minimum number of samples, following Spriggs *et al*. (2019). We constructed four datasets: (1) min4: 75 178 loci shared by ≥ 4 individuals with 53.46% missing data (the original dataset); (2) min20: 54 169 loci shared by ≥ 20 individuals with 50.84% missing data; (3) min40: 46 851 loci shared by ≥ 40 individuals and 48.82% missing data; and (4) min60: 36 098 loci shared by ≥ 60 individuals with 46.87% missing data.

To ease the computational burden of downstream analyses, we created a reduced set of 136 samples in which each geographically defined population was represented by three individuals. This set was assembled with the locus filtering criteria mentioned above, resulting in four datasets: (1) taxa136: 72 398 loci and 50.55% missing data; (2) taxa136‐min20: 51803 loci and 47.39% missing data; (3) taxa136‐min40: 43894 loci and 44.58% missing data; and (4) taxa136‐min60: 38150 loci and 41.50% missing data.

To assess population genetic structure, we used clustering approaches without pre‐assigned populations in *ADMIXTURE* (Alexander *et al*., 2009). We determined the optimal number of genetic clusters *K* by cross‐validation (CV) error. To uncover finer‐scale clusters, we applied a hierarchical approach (Massatti & Knowles, 2014; Spriggs *et al*., 2019) in which we ran *ADMIXTURE* only on samples of *A. acuminatus*, excluding outgroup species.

We reconstructed the population tree using *tetrad*, part of the ipyrad toolkit (Eaton & Overcast, 2020), estimating clade support with 100 bootstrap replicates. We also constructed maximum‐likelihood phylogenies from concatenated SNP matrices with iq‐tree v.2.1.4‐beta (Minh *et al*., 2020) using the GTR + ASC model (Lewis, 2001) with ultrafast bootstraps (UFBoot, Minh *et al*., 2013).

To investigate patterns of historical divergence and gene flow between populations, we used *TreeMix* (Pickrell & Pritchard, 2012). We grouped individuals into populations based on geography and monophyly in the reconstructed *tetrad* phylogenies. We used both the full set of 195 samples and the subset of 136 samples, filtered to retain loci shared by at least 50% of individuals in each population. We fit models with 0–5 numbers of admixture edges (m) and compared the log‐likelihood scores. We randomly subsampled 1 SNP per locus with 9 iterations and compared the topologies and inferred admixture events. We also estimated genetic divergence by the pairwise fixation index (*F*_ST_) using vcftools (Danecek *et al*., 2011), employing the same population groupings.

### Pollination biology

Reproductive success of *A. acuminatus* relies on pollination by animal visitors. Similar to other congeneric species, the species is strongly protandrous with stamens and pistils separated spatially at each sexual stage: in the male phase, anthers are exserted and dehiscing while the stigma remains enclosed within the corolla; in the female phase, anthers have withered, the style elongates, and the stigma becomes receptive and exserted (Middleton, 2007; Chen *et al*., 2019). Previous reproductive experiments in Taiwan have demonstrated that the species is self‐compatible, but cannot achieve autonomous selfing (Chen *et al*., 2019).

Pollinator observations were conducted in mainland populations of *A. acuminatus* at 1 site in Lâm Đồng (LD) in southern Vietnam, 2 sites in Yunnan (YN) in southwestern China, and 2 sites in Fujian (FJ) in southeastern China, to cover its geographic range (Fig. 1). We also made observations at 6 sites in Taiwan (TW) where pollinators of *A. acuminatus* had not been previously studied (Chen *et al*., 2019).

To identify pollinators and record their visitation frequency and behavior, we conducted direct observations and utilized motion‐triggered cameras (Bushnell NatureView HD Cam, Model 119 740, Krauss *et al*., 2018; Johnson & Van der Niet, 2019). Cameras were positioned at a distance of 0.4–1.0 m from the target inflorescences with 1–4 flowers open. Three photos and a 20‐s‐long video were recorded upon triggers. The sensor levels of the motion‐triggered cameras were set to high. We also conducted direct human observations occasionally at all study sites (see Table S2 for total observation hours).

To assess pollinator assemblages, we reviewed visits documented by photos and videos. We categorized visiting animals into three pollination functional groups: sunbirds, generalist passerines, and rodents (Methods S1). To compare pollinator abundance among sites, we estimated visitation frequency, dividing the total number of visits by the total observation hours at a given site. Each identifiable visit by any animal to the flowers of *A. acuminatus* was counted as a single visit. We did not normalize estimates by the total number of flowers within the field of view of the cameras or the visual range of observers, so they represented estimates of overall visitor frequency and abundance in the studied population.

We further evaluated the pollination efficacy of each functional group across populations. We first determined whether a visit was potentially legitimate pollination by actual contact between the body parts of visitors and the stamens and stigmas. While the dimensions of mouthparts differ among the three pollinator function groups, effective contacts were made mostly on the front sides of pollinator heads, that is foreheads or throats of birds and front muzzles of rodents. We then estimated pollinator importance as the product of visitation rates (quantitative components) and contact rates (qualitative components; Moir *et al*., 2025; Lagomarsino & Muchhala, 2019). At locations with multiple functional groups of pollinators, we calculated the relative importance by scaling to 1.0. We estimated pollinator importance only for visitation datasets for which contact rates could be counted, that is, direction observations in southern Vietnam and all camera traps. We acknowledged that the low visitation rates in our study system may render contact rate estimates susceptible to sampling error; therefore, pollinator importance should be interpreted with caution.

### Floral traits

At each study site, we sampled 5–15 individual flowers to quantify floral traits. If fewer than 5 flowers were open, we sampled the maximum number of available flowers. We focused on floral traits associated with pollinator interactions: corolla tube length (dorsal and ventral), width of corolla opening, lengths of the didynamous stamens, stigma length in female stage flowers, peduncle and pedicel length (PedPed length), and calyx length. Due to protandry in *A. acuminatus*, we assembled separate data sets for male‐ and female‐staged flowers. To visualize the floral morphospace, we conducted principal components analyses (PCA) using the prcomp function in R and visualized the axes using the autoplot function in the ggfortify R package (Tang *et al*., 2016).

We then tested whether floral morphology differed among pollinator groups. For each floral trait and the first two PC axes of male‐ and female‐staged data sets, we first applied the Shapiro–Wilk test for normality and Levene's test for equality of variances. Almost all variables violate the assumptions of either normality or homogeneity of variances across groups (Table S3), so we conducted Welch's ANOVA for individual traits and the four PC axes among the three pollinator functional groups. We then performed Games‐Howell *post‐hoc* comparisons among groups for each variable using the R package rstatix (Kassambara, 2023). If floral traits covaried with local pollinators, we expected populations pollinated by generalist passerines to have shorter corolla tubes, wider corolla openings, shorter stamens and stigmas, and shorter PedPed lengths (see Methods S1). In contrast, populations with sunbird pollination would occupy the other end of the spectrum, while populations with mixed pollination lie in between.

## Results

### Phylogeography

Our sequencing generated a median of 3.56 M reads per sample, with a median of 3.47 M (97.4%) passing quality filters. We mapped a median of 1.91 M reads per sample to our *A. acuminatus* reference genome. After filtering for low read coverage (< 6), we assembled a median of 44 776 clusters per sample. Clustering across samples yielded a final dataset comprising 2 733 523 SNPs from 75 178 GBS loci. Data sets with different filtering criteria yielded 41.50–53.46% missing data. More stringent filtering criteria reduced the degree of unevenness in loci shared by individual samples. We observed no systematic bias in missing data (Heatmap of shared loci in Fig. S1).

*ADMIXTURE* analyses of the full taxon sample with 108 117 SNPs (after filtering SNPs shared across < 75% of all samples) showed continuously decreasing cross‐validation errors with higher values of *K* (Fig. S2a,b). Here, we present the results for $K\in \left\{3,5\right\}$, which yielded significant decreases in CV error (Fig. S2c). In particular, at K=3, genetic clusters correspond to the populations of *A. acuminatus* in southern Vietnam, the remaining *A. acuminatus*, and *A. bracteatus* from south and southeastern Yunnan. All other outgroups, including *A. bracteatus* from western Yunnan, are admixtures of these clusters. Analysis of *A. acuminatus* without outgroup samples at K=3 identified three genetic clusters corresponding to southern Vietnam, Taiwan, and mainland populations in China, with the easternmost mainland populations sharing more alleles with Taiwanese populations (Fig. S2d).

**Fig. 3:**
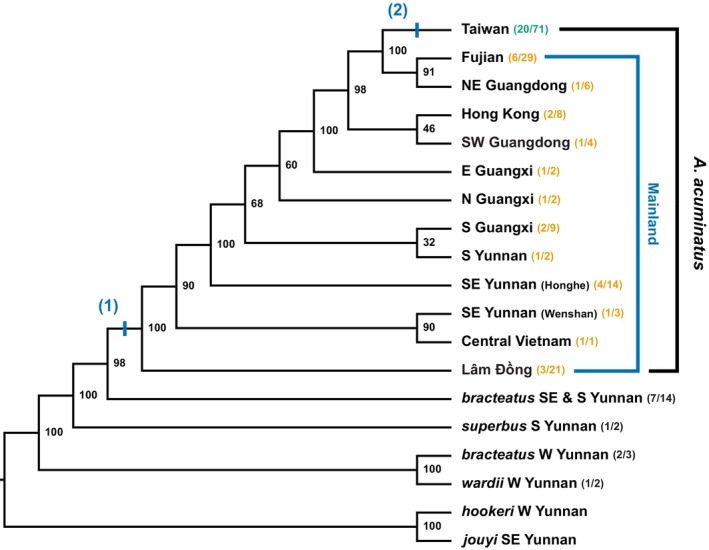
Population tree of *Aeschynanthus acuminatus* and related species estimated using the full set of 195 samples. Terminal branches represent monophyletic groups and are labeled by geographic location and the number of sampled populations and individuals. Node labels show bootstrap values. Vertical bars on branches mark key events: (1) speciation of *A. acuminatus* on the mainland; and (2) colonization of Taiwan.

The population trees reconstructed by *tetrad* across levels of locus filtering (min4, min20, min40, min60) and sample sets (full and taxa136) had similar topologies (Fig. S3). The maximum likelihood trees inferred from concatenated data were similar, with only minor differences in mainland population relationships, usually involving nodes without strong support (Fig. S4). We present the *tetrad* tree from the full dataset (195 samples, 2 733 523 SNPs from 72 445 loci; 2 582 836 out of 58 409 520 quartets sampled; Fig. 3) as the basis for further discussion.

In the *tetrad* tree, most major clades corresponding to geographically defined populations and internal nodes have high bootstrap support (> 90, Figs 3, S3), with a branching sequence that generally follows a west‐to‐east pattern. Individuals of *A. acuminatus* form a well‐supported clade sister to a lineage of *A. bracteatus* from SE and S Yunnan (Fig. 3). Within *A. acuminatus*, the southern Vietnam (Lâm Đồng) lineage diverges first and is sister to the rest. Populations from central Vietnam, southwestern China, and southeastern China diverge successively from the remaining populations. Samples from Taiwan form a strongly supported clade nested within this grade, sister to the Fujian and Guangdong populations of southeastern China. The topology of the *tetrad* tree thus fails to support the hypothesis that *A. acuminatus* originated on Taiwan, and is instead consistent with a mainland origin with subsequent range expansion to Taiwan.

*TreeMix* recovered a similar topology in which the Taiwan populations are deeply nested within the mainland populations (Fig. S5). Across all iterations in models with $m\in \left\{1,\ldots ,5\right\}$, the relationships among populations remain consistent, and none of the inferred genetic migration events involved the Taiwan clade, indicating that its phylogenetic position is unlikely to be a product of gene flow from nearby mainland populations. *F*_ST_ patterns among populations generally paralleled the west‐to‐east divergence history recovered by *tetrad*, with the Lâm Đồng population in southern Vietnam being the most genetically isolated (Table S4).

### Pollination biology

**Fig. 4:**
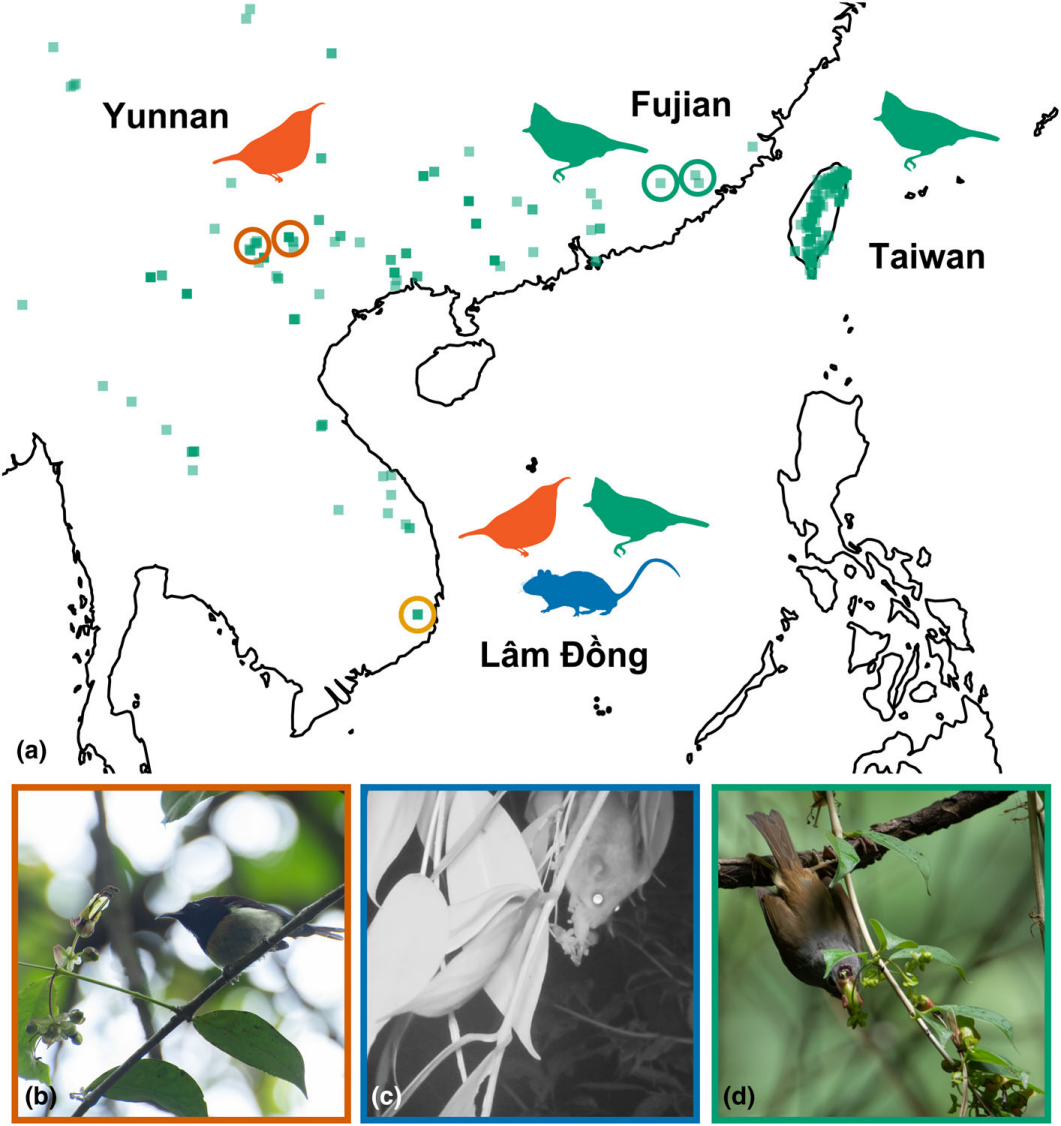
Geographic variation and functional groups of floral visitors (a) Summary of observed pollinator assemblages across the geographic range of *Aeschynanthus acuminatus*. Green squares indicate occurrences of *A. acuminatus*. Populations surveyed for the present study are circled, surrounded by silhouettes of observed pollinators at each location. Pollinators are indicated by color: vermilion, sunbirds; blue, rodents; and green, generalist birds. The orange circle indicates a mixed vertebrate pollination system. (b–d) Photographs showing floral visits of three pollinator functional groups. (b) Black‐throated Sunbird (*Aethopyga saturata*) perching on the branch after visiting flowers at Shibanzhai of southwestern China; (c) Nocturnal true mice (Muridae) visiting flowers of *A. acuminatus* for nectar at K'Long K'Lanh, southern Vietnam; (d) Huet's Fulvetta (*Alcippe hueti*) perching to visit flowers of *A. acuminatus* at Yixiantian, southeastern China, with its forehead making contact with stamens and stigma when probing for nectar.

Pollinator observations in 14 populations of *A. acuminatus* across its range showed varied degrees of visitation by both sunbirds and generalist passerines on mainland Asia (Fig. 4). Unexpectedly, we also observed rodent visitation in Vietnam. *A. acuminatus* thus exhibits a mixed vertebrate pollination system at the species level. Below, we describe regional visitation patterns in more detail and provide comprehensive lists in Tables S2 and S5.

#### Southern Vietnam

In Lâm Đồng, southern Vietnam, direct observations and multiple 2‐wk‐long camera traps revealed both sunbirds (Mrs. Gould's Sunbird, *Aethopyga gouldiae*, Nectariniidae) and generalist passerines (Mountain Fulvetta, *Alcippe peracensis*, Leiothrichidae) pollinating *A. acuminatus*. Both species made legitimate contact with the reproductive organs during their visits. The visitation frequencies of both functional groups of bird pollinators were surprisingly low in southern Vietnam. During 899 camera trapping and 28.5 direct observation hours, only two sunbird and two generalist passerine visits were observed (visitation rate = 0.0022 visits h^−1^).

In addition to avian pollinators, we also recorded unanticipated visits by small rodents (Note S1). Video recordings showed these small nocturnal and arboreal rodents, < 10 cm in length, climbing on branches and visiting flowers of *A. acuminatus* on consecutive nights. The rodents accessed nectar via the floral tube opening, and most flowers remained undamaged after the recording period. The observed behaviors suggest that rodents act as effective pollinators rather than flower foragers or nectar robbers. The rodents were also the most important pollinators among the three functional groups due to their relatively high visitation rate (relative pollinator importance = 0.571, Table S5).

#### Southwestern China

In Yunnan, we documented Black‐throated Sunbirds (*Aethopyga saturata*, Nectariniidae) pollinating *A. acuminatus* by both occasional observations and weeks‐long camera traps at two populations, Ladeng and Shibanzhai. In both populations, Black‐throated Sunbirds were the only flower visitors. Both direct observations and videos confirmed legitimate contact with reproductive organs when foraging for nectar, but the contact rates are lower compared to generalist passerines in other populations (25–43%; Table S5). Pollen was also visibly deposited on the heads of birds. Sunbirds visited flowers at a relatively high frequency compared to those in southern Vietnam (0.032–0.058 visits h^−1^; Table S5). We also observed an additional 134 visits during a 3.5‐h period of direct observation that included all visible flowers at Shibanzhai.

We documented generalist passerines nearby, but did not record confirmed visits and pollination. Yunnan Fulvetta (*Alcippe fratercula*, Leiothrichidae) perched near flowers of *A. acuminatus*, triggering camera traps at Shibanzhai. We cannot rule out the possibility that camera traps failed to capture short visits, but these visits should be far less frequent than those of sunbirds if they ever occurred. Whiskered Yuhinas (*Yuhina flavicollis*, Zosteropidae) and Blue‐winged Minlas (*Actinodura cyanouroptera*, Leiothrichidae) were observed forming foraging flocks at Ladeng but did not visit *A. acuminatus* at these sites.

#### Southeastern China

We documented a single species of generalist passerine, Huet's Fulvetta (*Alcippe hueti*, Leiothrichidae), pollinating *A. acuminatus* in two populations, Guxi in Fujian and Yixiantian in Guangdong. At Guxi, camera traps recorded frequent visits (0.037 visits h^−1^), while at Yixiantian, we observed a flock of six individuals visiting flowers. The generalist birds visited flowers with a high contact rate at Guxi (97.4%). A single sunbird species, the Fork‐tailed Sunbird (*Aethopyga christinae*, Nectariniidae), has a range that extends to SE China. We detected their presence through motion‐triggered cameras; however, no visits to the flowers were recorded.

#### Taiwan

The discovery of rodent pollination in Lâm Đồng, Vietnam, raises the possibility that this phenomenon may also occur in other East Asian regions. However, in Taiwan, despite including additional sites across the island, we still observe only generalist passerines as pollinators.

We recorded multiple species of generalist passerines pollinating *A. acuminatus* that were not previously reported (Chen *et al*., 2019). These include the Taiwan Scimitar‐Babbler (*Pomatorhinus musicus*, Timaliidae), Rufous‐capped Babbler (*Cyanoderma ruficeps*, Timaliidae), Black Bulbul (*Hypsipetes leucocephalus*, Pycnonotidae), and Taiwan Barwing (*Actinodura morrisoniana*, Leiothrichidae) across five study sites (Table S2). In populations with camera traps, these generalist passerines visited flowers with frequencies comparable to the mainland populations (0.0180–0.0320 visits h^−1^) and perfect contact rates (100%; Table S5).

### Floral traits

**Fig. 5:**
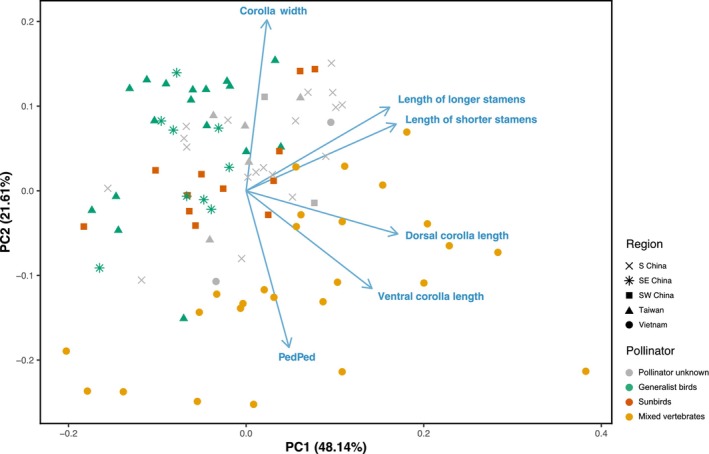
Principal component analysis (PCA) of floral traits shows no obvious adaptation to local pollinator assemblages. Each point represents an individual flower of male‐staged *Aeschynanthus acuminatus*. Color indicates pollinators: green, generalist birds; orange, mixed vertebrates; vermilion, sunbirds; gray, pollinator unknown. Point shapes depict geographic regions. Blue arrows indicate floral trait variable loadings.

We made 212 trait measurements of individual flowers of *A. acuminatus* from 23 populations. Most floral traits show strong positive correlations, suggesting a single functional module (Fig. S6). The strongest correlations are between stamen lengths and corolla tube lengths. The male‐stage dataset (92 individuals, Fig. 5) and the female‐stage dataset (81 individuals, Fig. S7) show similar results. We hereafter describe the findings based on the male‐stage dataset. We also removed calyx measurements from the main analyses to reduce missing data, but observed similar trends when they were included.

The first PC axis represents 48.14% of the total variation in floral morphospace, and most floral traits, except corolla width and PedPed length, align with this axis (eigenvectors in Fig. 5). PC1 does not completely separate populations by pollinator group. However, mixed‐vertebrate pollination (sunbirds, generalist passerines, and rodents) in southern Vietnam is associated with significantly higher trait variances than pollination by sunbirds or generalist passerines alone (Table S6). The second PC axis explains 21.61% of the total floral variation, and is associated with the corolla opening width and PedPed length (in opposite directions). Within *A. acuminatus*, populations with mixed vertebrate pollinators occupy a larger region of floral morphospace (Fig. 5).

Welch's ANOVA indicates that in five out of seven floral traits, and all four selected PC axes, the means are significantly different among pollinator groups (Table S7). In most of these variables, *post‐hoc* comparisons revealed that populations with mixed‐vertebrate pollination differ from those pollinated by sunbirds or generalist passerines alone (Table S8). Flowers in mixed‐vertebrate pollinated populations tend to have longer corollas, narrower corolla openings, and longer PedPed lengths. In contrast, most variables were not different between populations pollinated by generalist passerines and those by sunbirds (Table S8). Flowers in populations pollinated by generalist passerines were only significantly smaller in corolla tube length measurements.

## Discussion

### Lack of support for Grant‐Stebbins predictions

Despite its deep roots, the Grant‐Stebbins model was only recently formulated as such (Johnson, 2006, 2010), and studies of its fit to empirical systems remain limited (see Van der Niet *et al*., 2014b). The model predicts microevolutionary responses to geographic mosaics of pollinators (local adaptation to the most effective pollinator) with macroevolutionary consequences (the origin of species adapted to pollinators different from their ancestors). In this study, we integrate evidence from pollinator observations, floral trait variation, ancestral state estimation, and phylogeography to test both the microevolutionary and macroevolutionary aspects of the model in *A. acuminatus*.

Contrary to Grant‐Stebbins predictions, we found that variation in floral traits does not show obvious ecotypic associations with local pollinator faunas, and ancestral reconstructions indicated that the species did not originate beyond the range of sunbirds in Taiwan through a shift to generalist passerine pollination. Instead, it evolved on the mainland in the presence of both types of birds (Scenario b; Fig. 2b). These unexpected results raise questions about how they might be explained by existing conceptual frameworks for pollinator‐mediated floral evolution.

### How did generalized pollination evolve in *Aeschynanthus acuminatus*?

The evolution of generalized mixed‐vertebrate pollination from sunbird specialization in *A. acuminatus* runs counter to the more typical expectation that specialists evolve from generalists (Futuyma & Moreno, 1988; Barrett, 2013). However, transitions from specialized to generalized pollination systems are not uncommon (Armbruster & Baldwin, 1998; Tripp & Manos, 2008; Armbruster, 2017), and may even be labile, as in the case of secondary specialization through paedomorphosis in *Dalechampia* (Armbruster *et al*., 2013). In *Passiflora*, specialized pollination by Sword‐billed Hummingbirds (*Ensifera ensifera*) transitioned multiple times to other vertebrate pollinators (Abrahamczyk *et al*., 2014). Generalized bird and mixed‐vertebrate pollination has also evolved from highly specialized insect pollination systems. For example, in South American Merianieae (Melastomataceae), the evolution of novel food bodies for passerine birds characterized the shift away from specialized buzz pollination by bees (Dellinger *et al*., 2019).

**Fig. 6:**
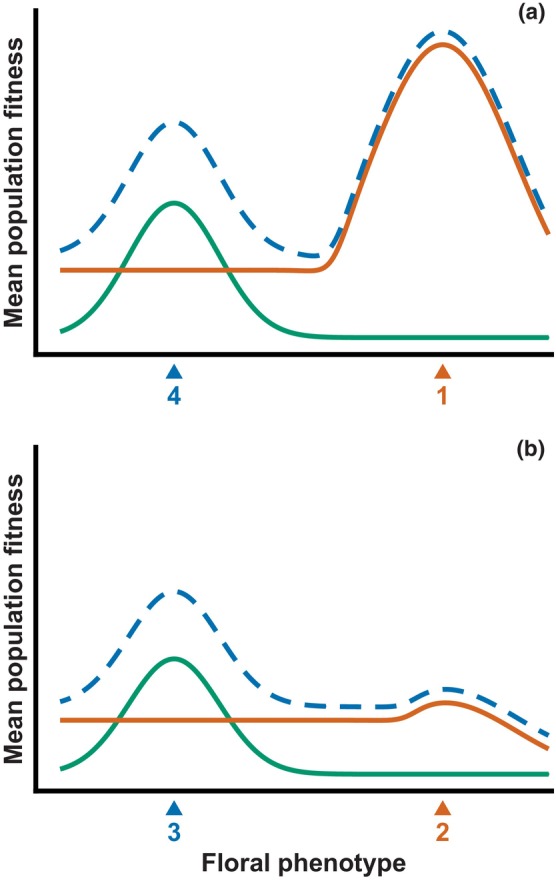
Hypothetical scenario by which *Aeschynanthus acuminatus* could have originated from a sunbird‐specialized ancestor via a shift to pollination by sunbirds and generalist birds. In plots a and b, fitness contributed by pollination is shown for sunbirds (vermilion), generalist birds (green), and their sum (blue dashed, offset for visibility). (a) In the ancestral population, the floral phenotype was maintained by stabilizing selection at the adaptive optimum for sunbird visitation (1), separated from the adaptive optimum for generalist birds by a valley induced by phenotypic trade‐offs. (b) During a period of reduced sunbird prevalence, fitness through sunbird pollination declined to a level at or below the valley, resulting in directional selection moving the mean population phenotype from (2 to 3). The phenotype at (3) is the adaptive optimum for generalist birds, but also allows visitation by sunbirds; in this region of morphospace, the flat sunbird curve ensures stabilizing selection around (3) even if sunbirds were to contribute greater fitness than generalist birds (not shown). The return of sunbird prevalence to previous levels (plot a) restored the adaptive valley, preventing the nascent *A. acuminatus* (4) from reverting to sunbird specialization.

Here, we use an optimality model to form a hypothesis about the origin and maintenance of generalization (pollination by sunbirds and generalist passerines) from specialization (pollination by sunbirds only). In this model, the realized fitness contribution of each pollinator group varies in relation to floral traits, represented by a univariate phenotype axis (Aigner, 2001). In this hypothetical adaptive landscape (Fig. 6a), the relatively long and narrow flowers of the ancestral population occupied a phenotypic optimum for high fitness through pollination by sunbirds, and low fitness through other pollinator groups. This ancestral phenotype was maintained by stabilizing selection arising from phenotypic trade‐offs, that is, local deviation from this optimum induced losses of fitness through reduced sunbird visitation without compensatory gains from other pollinators (Aigner, 2001). The landscape features a second adaptive optimum for pollination by generalist passerines, characterized by shorter and wider flowers. In this region of floral morphospace, as yet unoccupied in the ancestral population, sunbird pollination also contributes to fitness, but the curve is relatively flat. The additive fitness curve for sunbirds and generalist passerines thus has two distinct peaks separated by a valley.

We hypothesize that the origin of *A. acuminatus* began when the ancestral population experienced a period of reduced sunbird prevalence, lowering realized fitness via sunbird pollination to a level at or below the valley, reducing the strength of the phenotypic trade‐off between sunbirds and generalist passerines (Fig. 6b). The population then evolved shorter, wider flowers by unilateral selection (Ohashi *et al*., 2021), stabilizing on the optimum for generalist passerines. Note that this outcome is predicted even if the peak of the generalist passerine fitness curve is below the sunbird curve. When sunbird prevalence returned to normal levels, the new species was prevented from re‐evolving sunbird specialization by the adaptive valley between it and the sunbird optimum, leading to reproductive isolation and subsequent range expansion across mainland Asia and, eventually, to Taiwan.

### Little evidence for local adaptation in floral traits

Absolute and relative rates of sunbird and generalist passerine visitation to *A. acuminatus* vary across its range (Fig. 4; Table S5), suggesting a geographic mosaic that could potentially result in local adaptation to the most effective pollinator (Stebbins, 1970). However, the floral variation of *A. acuminatus* among populations mostly did not covary with local pollinator assemblages. The most significant variation in floral traits was in southern Vietnam, where we also observed the broadest array of pollinators. We would expect flowers adapted to generalized pollination to have relatively wide corolla tube openings; however, those in southern Vietnam are relatively narrow and exhibit greater variation in corolla tube length (Fig. 5; Table S6). We are investigating whether this could be the result of introgression from adjacent populations of co‐flowering *Aeschynanthus pedunculatus* (Lu *et al*., in preparation).

Corolla tube length was the only floral trait potentially demonstrating adaptation to local pollinators, with populations pollinated by generalist passerines having shorter corolla tubes. However, populations solely pollinated by sunbirds also had shorter corolla tubes than those with mixed vertebrate pollination, contrary to our predictions. Relatively low contact rates by sunbirds (< 50%; Table S5) further suggested a local functional mismatch in floral morphology in sunbird‐only populations. Our hypothesized adaptive landscape offers an explanation: the species is held at the phenotypic optimum for generalist passerine visitation by stabilizing selection, regardless of whether sunbirds are present at high or low frequencies, as in mainland Asia, or absent entirely, as in Taiwan. Interestingly, the high sunbird visitation rate in southwestern China potentially complemented the functional mismatch and resulted in a similar degree of pollinator importance to populations in southeastern China and Taiwan (Table S5), retaining the floral morphology near the generalist passerine optimum.

Lack of local adaptation to geographic variation in pollinator assemblages could also arise from ecological factors such as resource partitioning (Janzen, 1985; Thompson, 2005; Armbruster, 2017). The relative abundances of pollinators in a community can mediate biotic interactions related to competition and partitioning of food resources. For example, in the Ladeng population of southwestern China, black‐throated sunbirds are the exclusive pollinators, but generalist passerines (Whiskered Yuhinas and Blue‐winged Minlas) co‐occur in the same river valley. Both generalist passerine species have been confirmed to act as pollinators for other plant species (Zhang *et al*., 2012; Collar & Robson, 2020). During our observations, they fed only on fruits and insects, passing over flowers of *A. acuminatus* even when they were nearby. Foraging flocks of these birds occasionally encountered sunbirds, but no aggressive behavior occurred, suggesting that the two groups had partitioned their resources and did not actively compete for the nectar of *A. acuminatus*. By contrast, aggression seems to be common between generalist passerines and sunbirds in other communities. For example, generalist passerines, Swinhoe's White‐eyes (*Zosterops simplex*, Zosteropidae) and Black‐headed Sibias (*Heterophasia desgodinsi*, Leiothrichidae) fight aggressively against Black‐throated Sunbirds when foraging together on *Clematis leschenaultiana* (Ranunculaceae) in an adjacent location in southwestern China (Lu, personal observation).

### Future considerations

Are the floral phenotypes of *A. acuminatus* at or near the optimum for generalist passerine pollination? Confirming our hypothesized landscapes needs further microevolutionary studies of fitness responses along floral trait axes by different pollinator functional groups. The population in Lâm Đồng, southern Vietnam, is of particular interest for its unusual pollinator assemblage, floral variation, and potential hybridization with congeneric sunbird specialists.

Our research highlights the importance of integrating field studies of pollination with phylogeographic inferences to understand the dynamics of pollinator‐mediated speciation. The elusive nature of visiting passerines hinders the effectiveness of direct observation in close proximity to flowers, but camera traps allowed us to detect rare pollinators and made it easier to conduct broad geographic sampling (Krauss *et al*., 2018; Cozien *et al*., 2019). It is important to allow sufficient time to document low‐frequency visitors. For example, *c*. 900 h of video led to our documentation of visits by all three functional groups of pollinators in southern Vietnam. Our observations in mainland East Asia also confirmed the long‐speculated close association between *Aeschynanthus* and sunbirds (Middleton, 2007, 2016; Mansibang & Senarillos, 2022) and yielded new evidence of generalist passerines and rodents also acting as pollinators. The range‐wide variation in pollination in *A. acuminatus*, in contrast to its lack of apparent locally adapted floral variation, underscores the risks of using floral syndrome to predict pollinators, particularly when treating wide‐ranging species as single prediction units (Dellinger, 2020; Van der Niet, 2021).

## Competing interests

None declared.

## Author contributions

J‐YL and RHR conceptualized and designed this study. J‐YL conducted the pollination studies and field collections. Y‐WX and HTL assisted with the field work. J‐YL performed the pollination, genetic, and morphological analyses with guidance from RHR. J‐YL and RHR wrote the manuscript, and all authors edited and revised it.

## Disclaimer

The New Phytologist Foundation remains neutral with regard to jurisdictional claims in maps and in any institutional affiliations.

## Supporting information


**Fig. S1** Heatmap of shared loci among samples across eight data sets.
**Fig. S2** Cross‐validation errors (CV errors) and individual ancestry proportions in *ADMIXTURE* analyses.

**Fig. S3** Population tree of *Aeschynanthus acuminatus* and related species estimated using *tetrad* on SNP matrices of eight data sets.

**Fig. S4** Maximum likelihood phylogenetic inference of *Aeschynanthus acuminatus* and related species using IQ‐TREE analyses based on concatenated SNP matrices of eight data sets.

**Fig. S5** Results of *TreeMix* analyses.
**Fig. S6** Correlations between different floral trait measurements among three datasets.
**Fig. S7** Principal component analysis (PCA) of floral traits for the female‐staged individuals.
**Methods S1** Categorization of pollinator functional groups.
**Notes S1** Identification, habits, and morphology of rodent visitors in Vietnam.

**Table S1** List of samples, population, and region groupings in each analysis.
**Table S2** Observation hours, types, and identified pollinators at each study site.

**Table S3** Shapiro–Wilk tests for normality and Levene's tests for equality of variances among floral traits.
**Table S4** *F*_ST_ among populations.
**Table S5** Visitation rate, contact rate, and pollinator importance among pollinator functional groups across study sites.
**Table S6** Means and SD of floral trait measurements among pollinator functional groups.
**Table S7** Welch's ANOVA for testing floral morphology among pollinator functional groups.
**Table S8** Games‐Howell nonparametric *post‐hoc* tests for significant differences in floral traits between pollinator functional groups.
Please note: Wiley is not responsible for the content or functionality of any Supporting Information supplied by the authors. Any queries (other than missing material) should be directed to the *New Phytologist* Central Office.

## Data Availability

Raw sequence reads that support the findings of this study are openly available in the NCBI BioProject PRJNA1376515 at http://www.ncbi.nlm.nih.gov/bioproject/1376515. Additional data and R scripts supporting the findings of this study are available on the GitHub repository at https://github.com/jingyilu/grant‐stebbins‐aeschynanthus‐acuminatus.
