# Supplementary material for: Testing macroevolutionary predictions of the Grant‐Stebbins model in the origin of Aeschynanthus acuminatus
Source: New Phytol. 2026 Jan 27;249(6):3137–48. doi: 10.1111/nph.70871 (PMC12917478; doi:10.1111/nph.70871)
Supplement: Supplementary file 1 — Fig. S1 Heatmap of shared loci among samples across eight data sets. Fig. S2 Cross‐validation errors (CV errors) and individual ancestry proportions in ADMIXTURE analyses. [file NPH-249-3137-s005.pdf]

New Phytologist Supporting Information

Article title: Testing macroevolutionary predictions of the Grant-Stebbins model in the origin of *Aeschynanthus acuminatus*
Authors: Jing-Yi Lu, Yaowu Xing, Hong Truong Luu, Richard H Ree
Article acceptance date: 4 December 2025

Figure S1. Heatmaps of shared loci among samples across eight data sets.

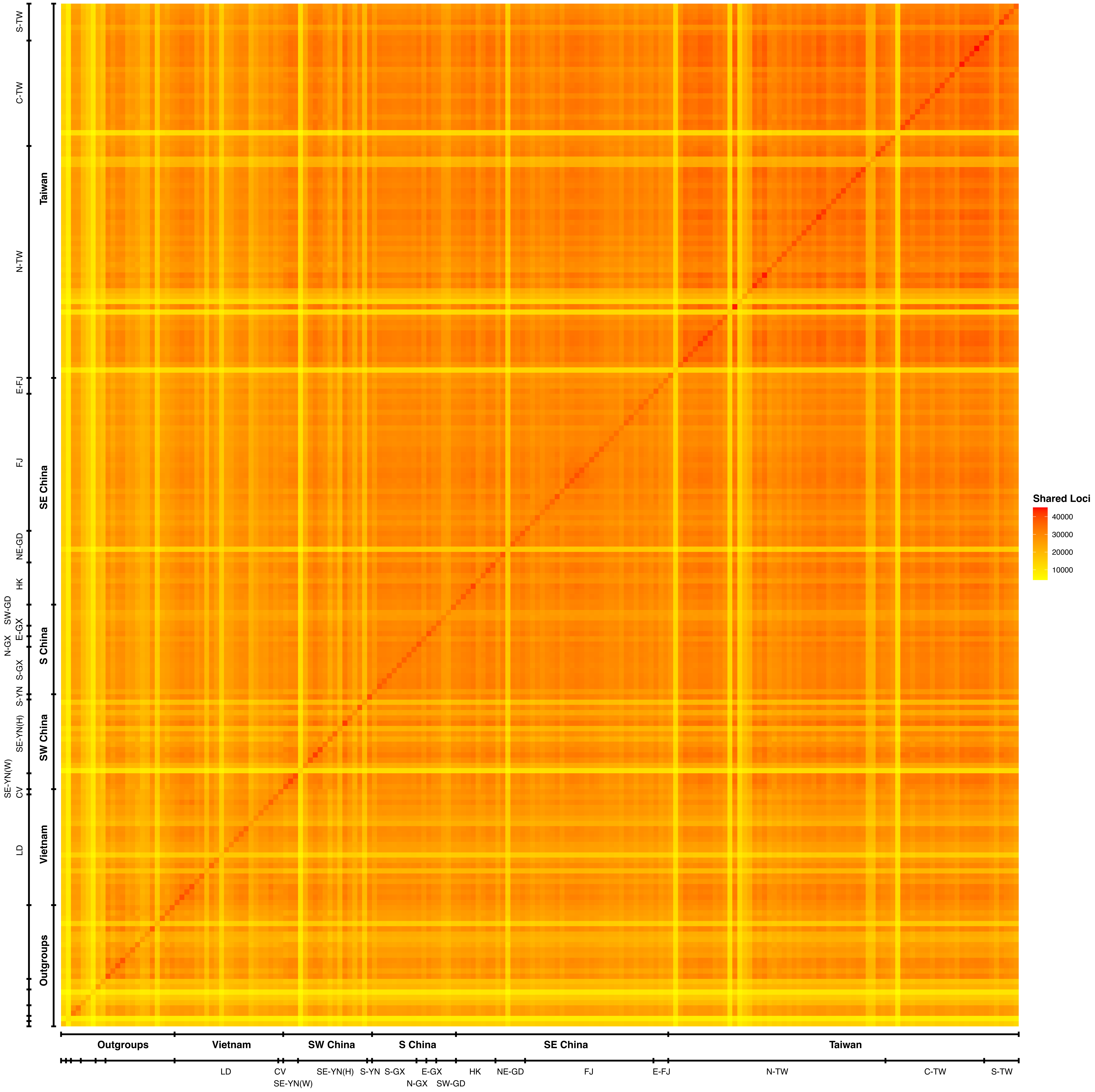

Data matrix: 195 taxa (min4)
72,445 total loci

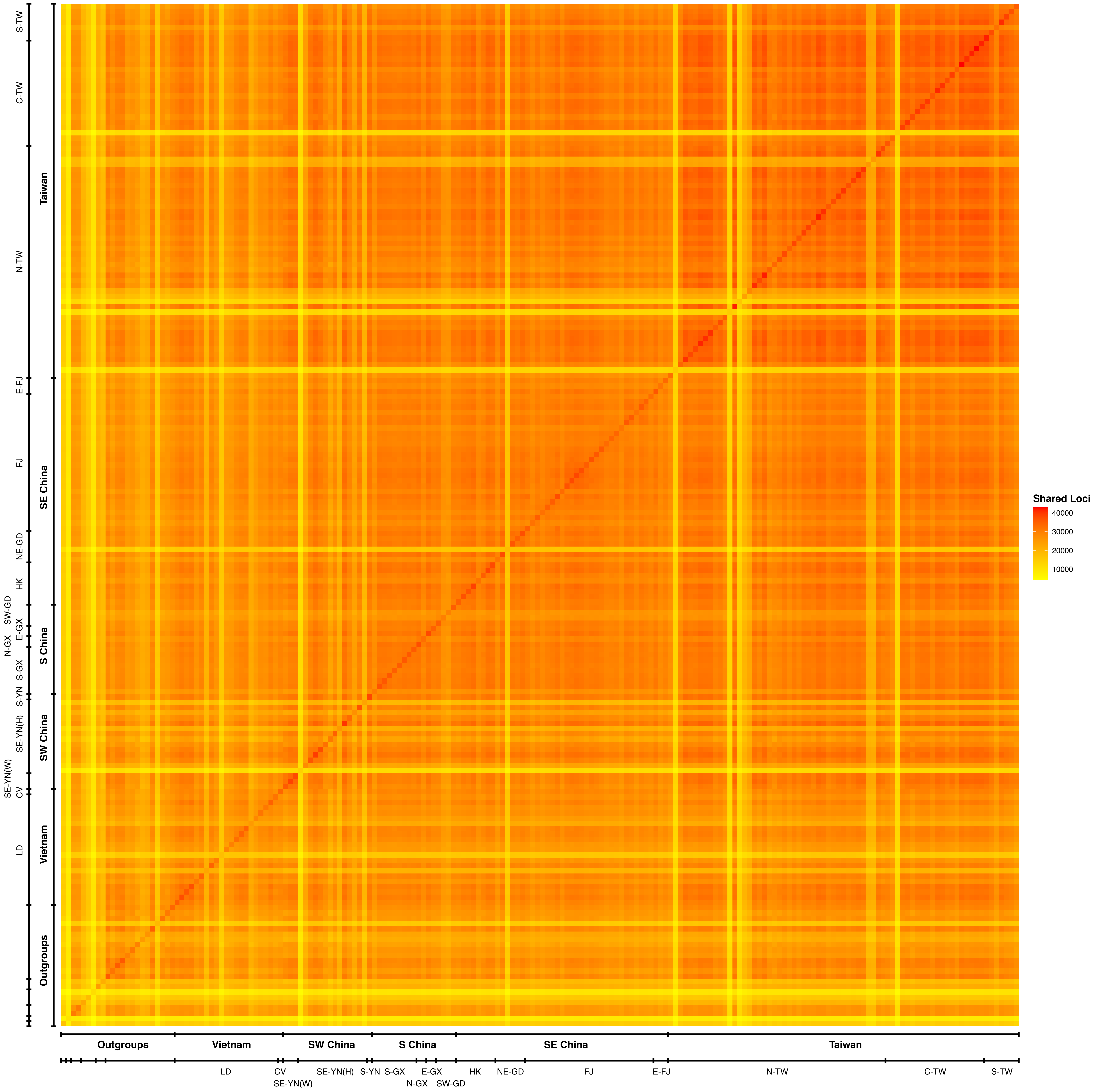

Data matrix: 195 taxa min20
53,928 total loci

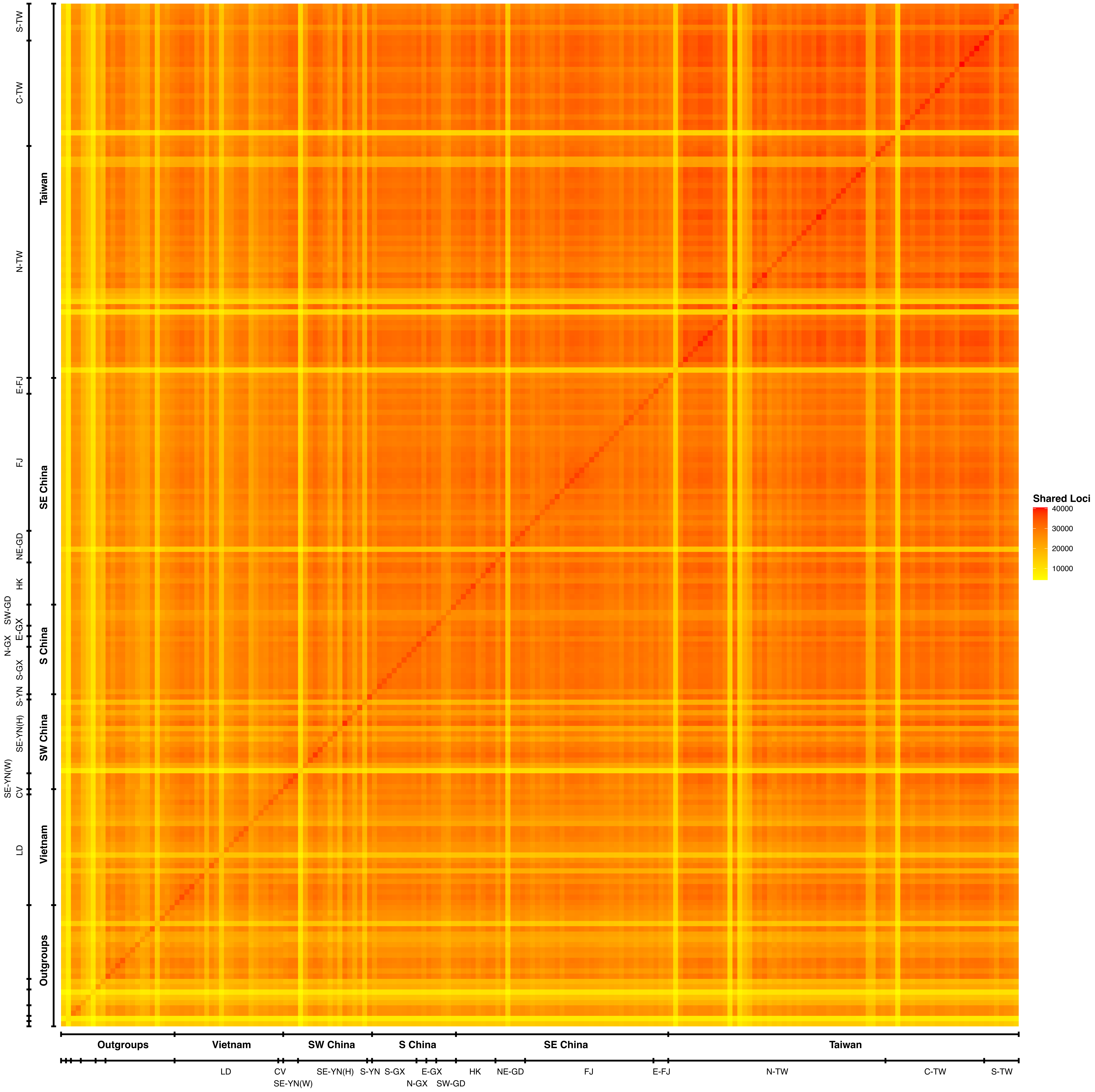

Data matrix: 195 taxa min40
46,776 total loci

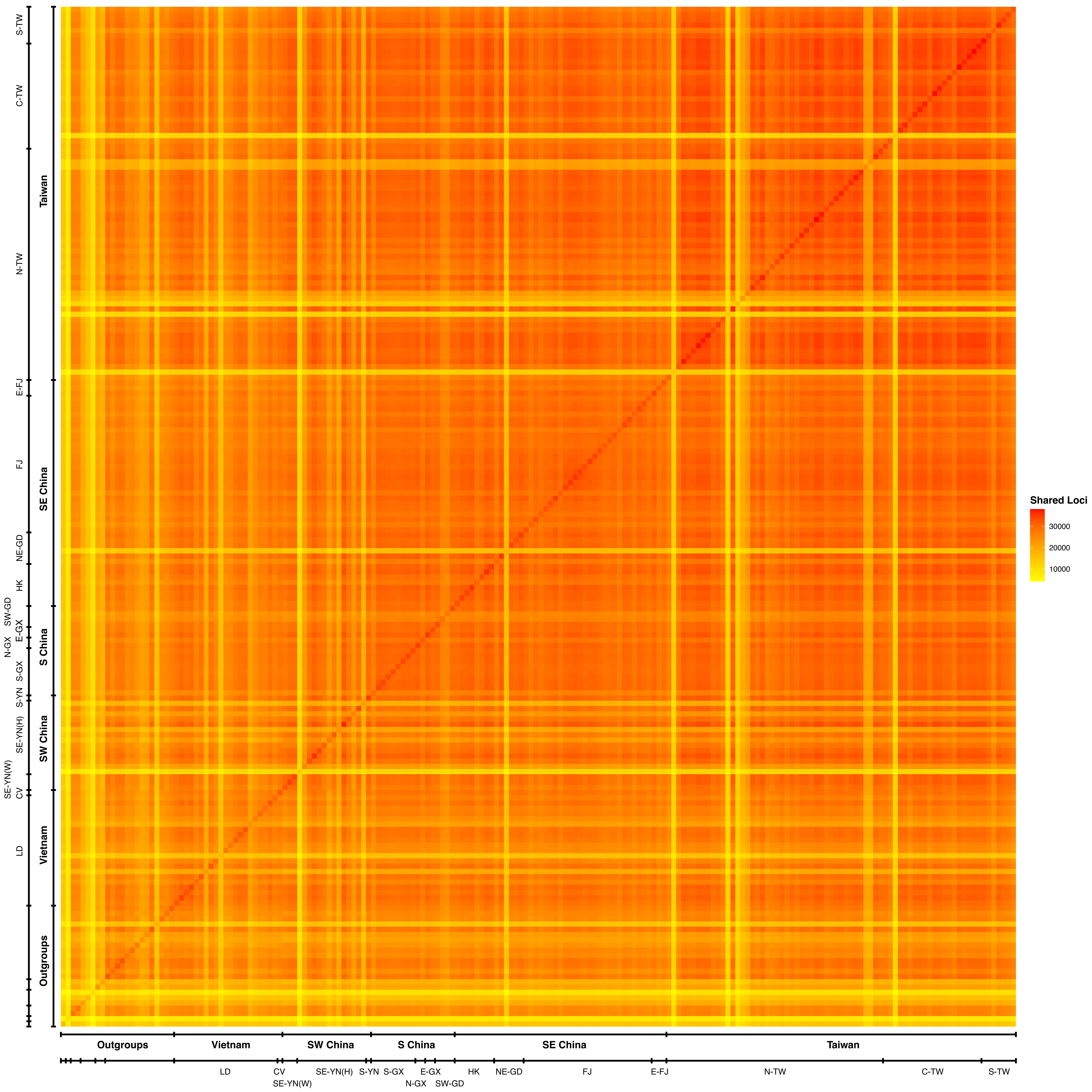

Data matrix: 195 taxa min60
41,990 total loci

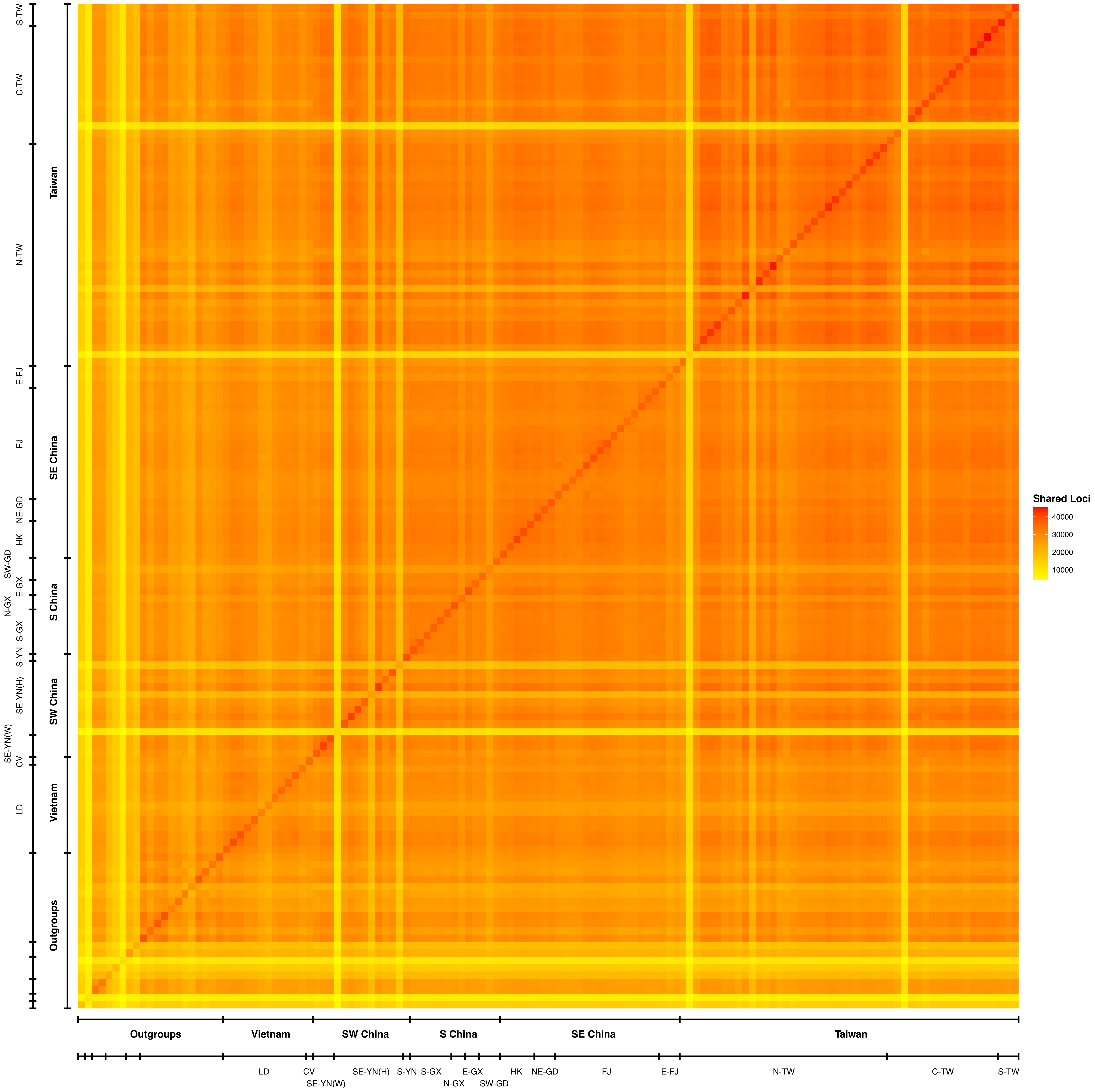

Data matrix: 136 taxa (min4)
69,834 total loci

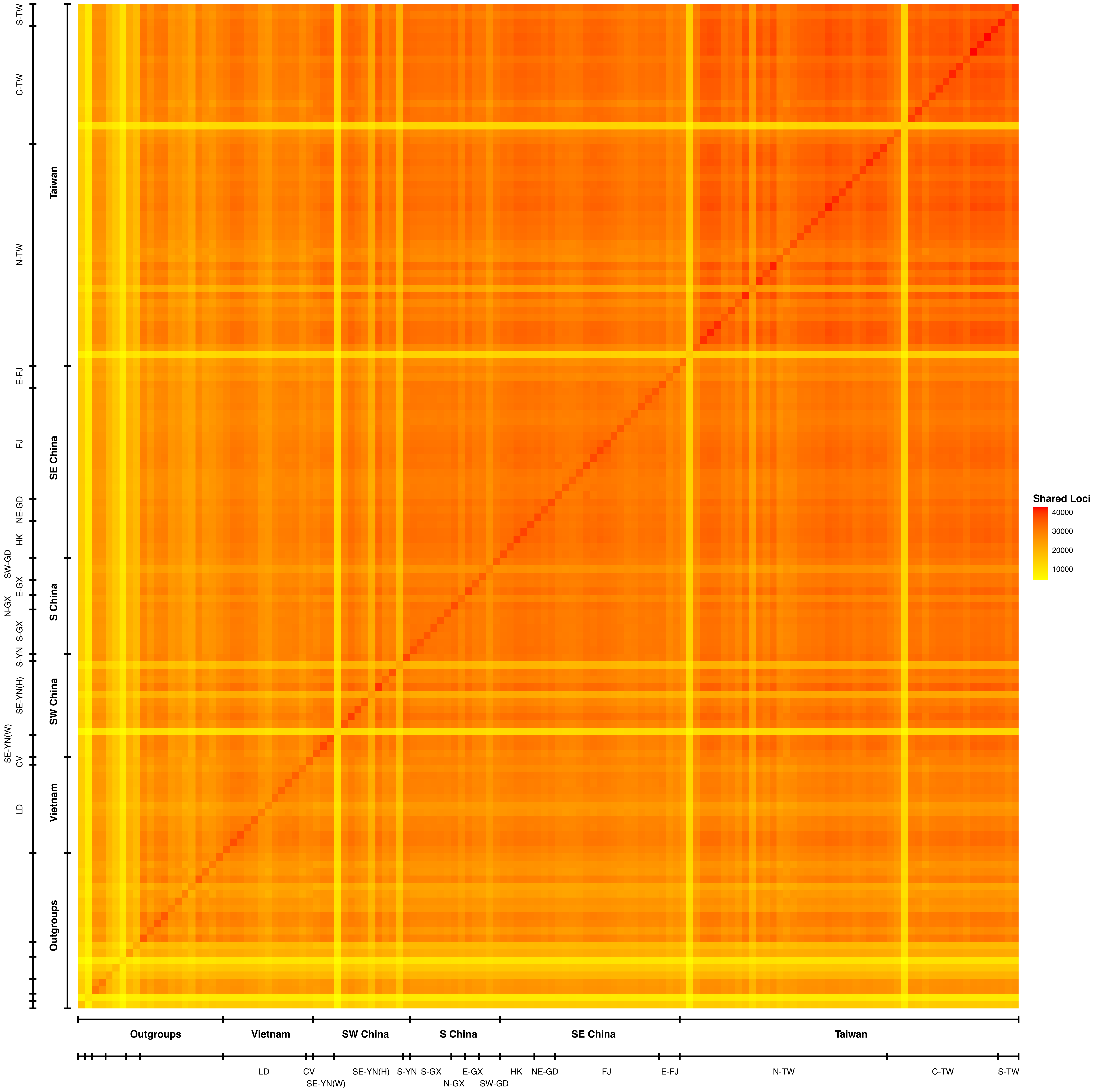

Data matrix: 136 taxa min20
51,534 total loci

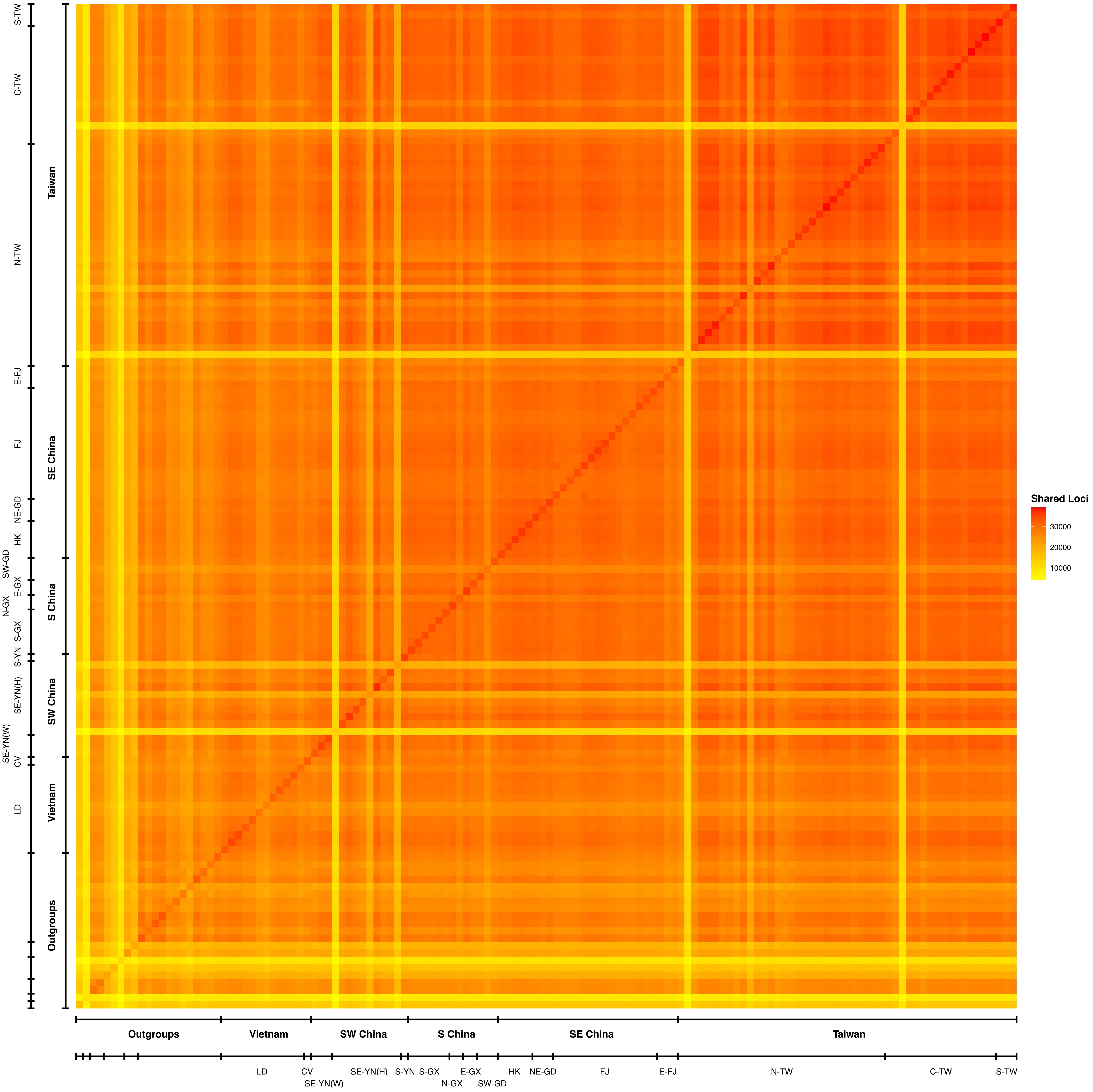

Data matrix: 136 taxa min40
43,817 total loci

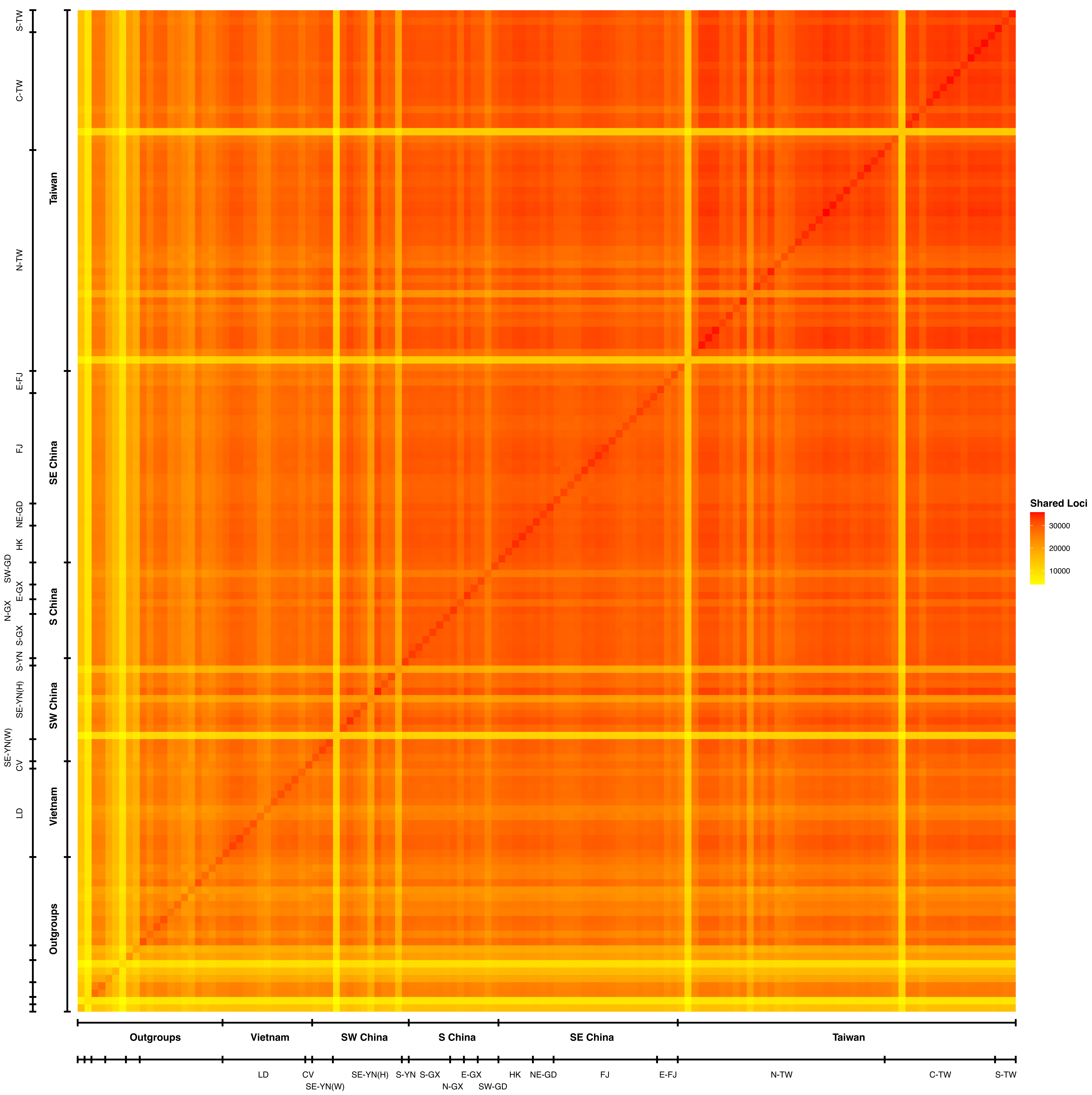

Data matrix: 136 taxa min60
38,110 total loci
