# Supplementary material for: Testing macroevolutionary predictions of the Grant‐Stebbins model in the origin of Aeschynanthus acuminatus
Source: New Phytol. 2026 Jan 27;249(6):3137–48. doi: 10.1111/nph.70871 (PMC12917478; doi:10.1111/nph.70871)
Supplement: Supplementary file 2 — Fig. S3 Population tree of Aeschynanthus acuminatus and related species estimated using tetrad on SNP matrices of eight data sets. [file NPH-249-3137-s002.pdf]

New Phytologist Supporting Information

Article title: Testing macroevolutionary predictions of the Grant-Stebbins model in the origin of *Aeschynanthus acuminatus*
Authors: Jing-Yi Lu, Yaowu Xing, Hong Truong Luu, Richard H Ree
Article acceptance date: 4 December 2025

Figure S3. Population tree of *Aeschynanthus acuminatus* and related species estimated using *tetrad* on SNP matrices of eight data sets. Terminal branches represent monophyletic groups and are labeled by geographic location and the number of sampled populations and individuals. Node labels show bootstrap values. Vertical bars on branches mark key events: (1) Speciation of *A. acuminatus* on the mainland and (2) colonization of Taiwan.

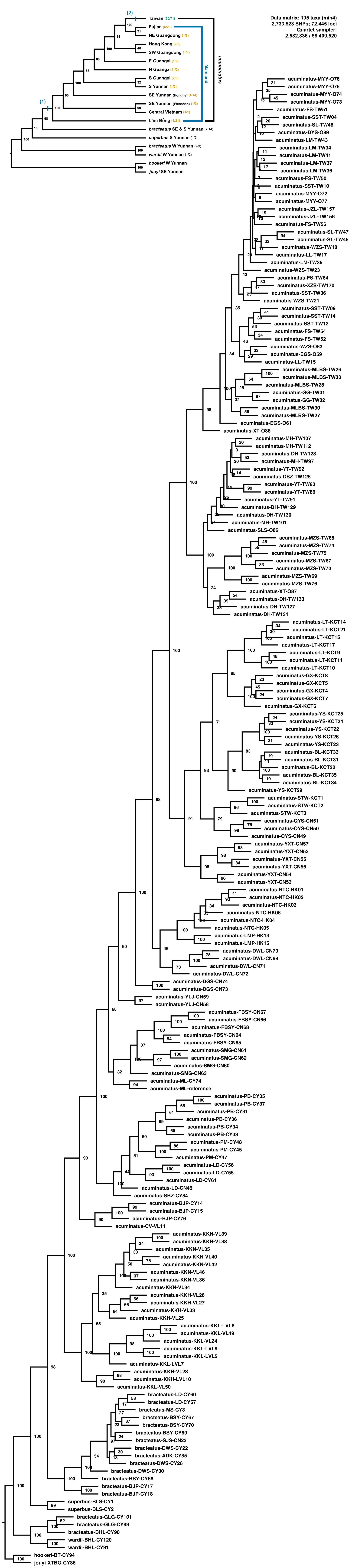

Data matrix: 195 taxa (min4)
2,733,523 SNPs; 72,445 loci
Quartet sampler:
2,582,836 / 58,409,520
(1)
(2)
Taiwan (20/71)
Fujian (6/29)
NE Guangdong (1/6)
Hong Kong (2/9)
SW Guangdong (1/4)
E Guangxi (1/2)
N Guangxi (1/2)
S Guangxi (2/9)
S Yunnan (1/2)
SE Yunnan (Honghe) (4/14)
SE Yunnan (Wenshan) (1/3)
Central Vietnam (1/1)
Lâm Đồng (3/21)
bracteatus SE & S Yunnan (7/14)
superbus S Yunnan (1/2)
bracteatus W Yunnan (2/3)
wardii W Yunnan (1/2)
hookeri W Yunnan
jouyi SE Yunnan
Mainland
acuminatus
acuminatus-MYY-O76
acuminatus-MYY-O75
acuminatus-MYY-O74
acuminatus-MYY-O73
acuminatus-FS-TW51
acuminatus-SST-TW04
acuminatus-SL-TW48
acuminatus-DYS-O89
acuminatus-LM-TW43
acuminatus-LM-TW34
acuminatus-LM-TW41
acuminatus-LM-TW37
acuminatus-LM-TW36
acuminatus-FS-TW50
acuminatus-SST-TW10
acuminatus-MYY-O72
acuminatus-MYY-O77
acuminatus-JZL-TW157
acuminatus-JZL-TW156
acuminatus-FS-TW56
acuminatus-SL-TW47
acuminatus-SL-TW45
acuminatus-WZS-TW18
acuminatus-LL-TW17
acuminatus-LM-TW35
acuminatus-WZS-TW23
acuminatus-FS-TW64
acuminatus-XZS-TW170
acuminatus-SST-TW06
acuminatus-WZS-TW21
acuminatus-SST-TW09
acuminatus-SST-TW14
acuminatus-SST-TW12
acuminatus-FS-TW54
acuminatus-FS-TW52
acuminatus-WZS-O63
acuminatus-EGS-O59
acuminatus-LL-TW15
acuminatus-MLBS-TW26
acuminatus-MLBS-TW33
acuminatus-MLBS-TW28
acuminatus-GG-TW01
acuminatus-GG-TW02
acuminatus-MLBS-TW30
acuminatus-MLBS-TW27
acuminatus-EGS-O61
acuminatus-XT-O88
acuminatus-MH-TW107
acuminatus-MH-TW112
acuminatus-DH-TW128
acuminatus-MH-TW97
acuminatus-YT-TW92
acuminatus-DSZ-TW125
acuminatus-YT-TW83
acuminatus-YT-TW86
acuminatus-YT-TW91
acuminatus-DH-TW129
acuminatus-DH-TW130
acuminatus-MH-TW101
acuminatus-SLS-O86
acuminatus-MZS-TW68
acuminatus-MZS-TW74
acuminatus-MZS-TW75
acuminatus-MZS-TW67
acuminatus-MZS-TW70
acuminatus-MZS-TW69
acuminatus-MZS-TW76
acuminatus-XT-O87
acuminatus-DH-TW133
acuminatus-DH-TW127
acuminatus-DH-TW131
acuminatus-LT-KCT14
acuminatus-LT-KCT21
acuminatus-LT-KCT15
acuminatus-LT-KCT17
acuminatus-LT-KCT9
acuminatus-LT-KCT11
acuminatus-LT-KCT10
acuminatus-GX-KCT8
acuminatus-GX-KCT5
acuminatus-GX-KCT4
acuminatus-GX-KCT7
acuminatus-GX-KCT6
acuminatus-YS-KCT25
acuminatus-YS-KCT24
acuminatus-YS-KCT22
acuminatus-YS-KCT26
acuminatus-YS-KCT23
acuminatus-BL-KCT33
acuminatus-BL-KCT31
acuminatus-BL-KCT32
acuminatus-BL-KCT35
acuminatus-BL-KCT34
acuminatus-YS-KCT29
acuminatus-STW-KCT1
acuminatus-STW-KCT2
acuminatus-STW-KCT3
acuminatus-QYS-CN51
acuminatus-QYS-CN50
acuminatus-QYS-CN49
acuminatus-YXT-CN57
acuminatus-YXT-CN52
acuminatus-YXT-CN55
acuminatus-YXT-CN56
acuminatus-YXT-CN54
acuminatus-YXT-CN53
acuminatus-NTC-HK01
acuminatus-NTC-HK02
acuminatus-NTC-HK03
acuminatus-NTC-HK06
acuminatus-NTC-HK04
acuminatus-NTC-HK05
acuminatus-LMP-HK13
acuminatus-LMP-HK15
acuminatus-DWL-CN70
acuminatus-DWL-CN69
acuminatus-DWL-CN71
acuminatus-DWL-CN72
acuminatus-DGS-CN74
acuminatus-DGS-CN73
acuminatus-YLJ-CN59
acuminatus-YLJ-CN58
acuminatus-FBSY-CN67
acuminatus-FBSY-CN66
acuminatus-FBSY-CN68
acuminatus-FBSY-CN64
acuminatus-FBSY-CN65
acuminatus-SMG-CN61
acuminatus-SMG-CN62
acuminatus-SMG-CN60
acuminatus-SMG-CN63
acuminatus-ML-CY74
acuminatus-ML-reference
acuminatus-PB-CY35
acuminatus-PB-CY37
acuminatus-PB-CY31
acuminatus-PB-CY36
acuminatus-PB-CY34
acuminatus-PB-CY33
acuminatus-PM-CY48
acuminatus-PM-CY45
acuminatus-PM-CY47
acuminatus-LD-CY56
acuminatus-LD-CY55
acuminatus-LD-CY61
acuminatus-LD-CN45
acuminatus-SBZ-CY84
acuminatus-BJP-CY14
acuminatus-BJP-CY15
acuminatus-BJP-CY76
acuminatus-CV-VL11
acuminatus-KKN-VL39
acuminatus-KKN-VL38
acuminatus-KKN-VL35
acuminatus-KKN-VL40
acuminatus-KKN-VL42
acuminatus-KKN-VL46
acuminatus-KKN-VL36
acuminatus-KKN-VL34
acuminatus-KKH-VL26
acuminatus-KKH-VL27
acuminatus-KKH-VL33
acuminatus-KKH-VL25
acuminatus-KKL-LVL8
acuminatus-KKL-VL49
acuminatus-KKL-VL24
acuminatus-KKL-LVL9
acuminatus-KKL-LVL5
acuminatus-KKL-LVL7
acuminatus-KKH-VL28
acuminatus-KKH-LVL10
acuminatus-KKL-VL50
bracteatus-LD-CY60
bracteatus-LD-CY57
bracteatus-MS-CY3
bracteatus-BSY-CY67
bracteatus-BSY-CY70
bracteatus-BSY-CY69
bracteatus-SJS-CN23
bracteatus-DWS-CY22
bracteatus-ADK-CY85
bracteatus-DWS-CY26
bracteatus-DWS-CY30
bracteatus-BSY-CY68
bracteatus-BJP-CY17
bracteatus-BJP-CY18
superbus-BLS-CY1
superbus-BLS-CY2
bracteatus-GLG-CY101
bracteatus-GLG-CY99
bracteatus-BHL-CY90
wardii-BHL-CY120
wardii-BHL-CY91
hookeri-BT-CY94
jouyi-XTBG-CY86

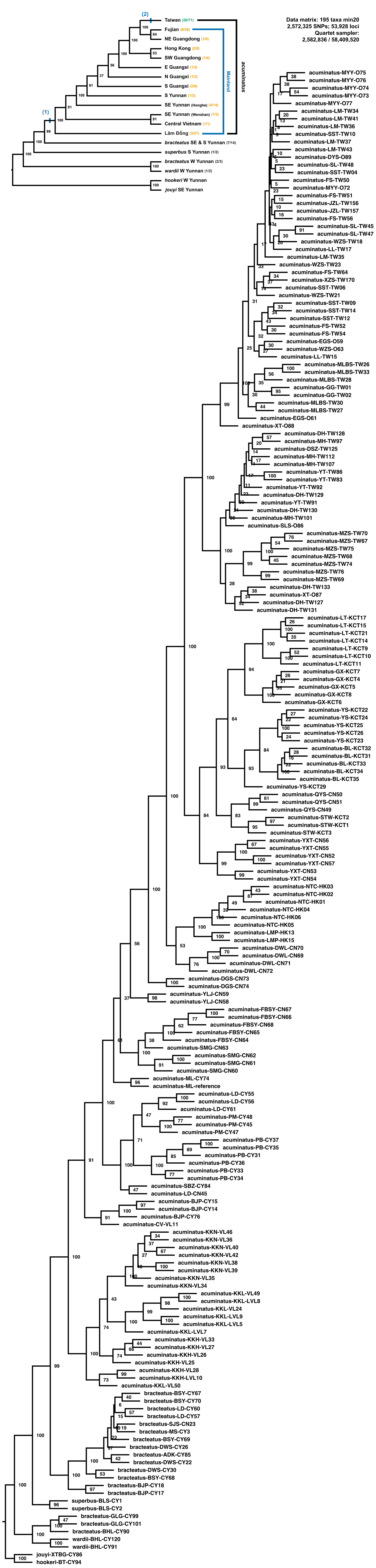

Data matrix: 195 taxa min20
2,572,325 SNPs; 53,928 loci
Quartet sampler:
2,582,836 / 58,409,520
(1)
(2)
Taiwan (20/71)
Fujian (6/28)
NE Guangdong (1/6)
Hong Kong (2/8)
SW Guangdong (1/4)
E Guangxi (1/2)
N Guangxi (1/2)
S Guangxi (2/9)
S Yunnan (1/2)
SE Yunnan (Honghe) (4/14)
SE Yunnan (Wenshan) (1/3)
Central Vietnam (1/1)
Lâm Đồng (3/21)
Mainland
acuminatus
bracteatus SE & S Yunnan (7/14)
superbus S Yunnan (1/2)
bracteatus W Yunnan (2/3)
wardii W Yunnan (1/2)
hookeri W Yunnan
jouyi SE Yunnan
acuminatus-MYY-O75
acuminatus-MYY-O76
acuminatus-MYY-O74
acuminatus-MYY-O73
acuminatus-MYY-O77
acuminatus-LM-TW34
acuminatus-LM-TW41
acuminatus-LM-TW36
acuminatus-SST-TW10
acuminatus-LM-TW37
acuminatus-LM-TW43
acuminatus-DYS-O89
acuminatus-SL-TW48
acuminatus-SST-TW04
acuminatus-FS-TW50
acuminatus-MYY-O72
acuminatus-FS-TW51
acuminatus-JZL-TW156
acuminatus-JZL-TW157
acuminatus-FS-TW56
acuminatus-SL-TW45
acuminatus-SL-TW47
acuminatus-WZS-TW18
acuminatus-LL-TW17
acuminatus-LM-TW35
acuminatus-WZS-TW23
acuminatus-FS-TW64
acuminatus-XZS-TW170
acuminatus-SST-TW06
acuminatus-WZS-TW21
acuminatus-SST-TW09
acuminatus-SST-TW14
acuminatus-SST-TW12
acuminatus-FS-TW52
acuminatus-FS-TW54
acuminatus-EGS-O59
acuminatus-WZS-O63
acuminatus-LL-TW15
acuminatus-MLBS-TW26
acuminatus-MLBS-TW33
acuminatus-MLBS-TW28
acuminatus-GG-TW01
acuminatus-GG-TW02
acuminatus-MLBS-TW30
acuminatus-MLBS-TW27
acuminatus-EGS-O61
acuminatus-XT-O88
acuminatus-DH-TW128
acuminatus-MH-TW97
acuminatus-DSZ-TW125
acuminatus-MH-TW112
acuminatus-MH-TW107
acuminatus-YT-TW86
acuminatus-YT-TW83
acuminatus-YT-TW92
acuminatus-DH-TW129
acuminatus-YT-TW91
acuminatus-DH-TW130
acuminatus-MH-TW101
acuminatus-SLS-O86
acuminatus-MZS-TW70
acuminatus-MZS-TW67
acuminatus-MZS-TW75
acuminatus-MZS-TW68
acuminatus-MZS-TW74
acuminatus-MZS-TW76
acuminatus-MZS-TW69
acuminatus-DH-TW133
acuminatus-XT-O87
acuminatus-DH-TW127
acuminatus-DH-TW131
acuminatus-LT-KCT17
acuminatus-LT-KCT15
acuminatus-LT-KCT21
acuminatus-LT-KCT14
acuminatus-LT-KCT9
acuminatus-LT-KCT10
acuminatus-LT-KCT11
acuminatus-GX-KCT7
acuminatus-GX-KCT4
acuminatus-GX-KCT5
acuminatus-GX-KCT8
acuminatus-GX-KCT6
acuminatus-YS-KCT22
acuminatus-YS-KCT24
acuminatus-YS-KCT25
acuminatus-YS-KCT26
acuminatus-YS-KCT23
acuminatus-BL-KCT32
acuminatus-BL-KCT31
acuminatus-BL-KCT33
acuminatus-BL-KCT34
acuminatus-BL-KCT35
acuminatus-YS-KCT29
acuminatus-QYS-CN50
acuminatus-QYS-CN51
acuminatus-QYS-CN49
acuminatus-STW-KCT2
acuminatus-STW-KCT1
acuminatus-STW-KCT3
acuminatus-YXT-CN56
acuminatus-YXT-CN55
acuminatus-YXT-CN52
acuminatus-YXT-CN57
acuminatus-YXT-CN53
acuminatus-YXT-CN54
acuminatus-NTC-HK03
acuminatus-NTC-HK02
acuminatus-NTC-HK01
acuminatus-NTC-HK04
acuminatus-NTC-HK06
acuminatus-NTC-HK05
acuminatus-LMP-HK13
acuminatus-LMP-HK15
acuminatus-DWL-CN70
acuminatus-DWL-CN69
acuminatus-DWL-CN71
acuminatus-DWL-CN72
acuminatus-DGS-CN73
acuminatus-DGS-CN74
acuminatus-YLJ-CN59
acuminatus-YLJ-CN58
acuminatus-FBSY-CN67
acuminatus-FBSY-CN66
acuminatus-FBSY-CN68
acuminatus-FBSY-CN65
acuminatus-FBSY-CN64
acuminatus-SMG-CN63
acuminatus-SMG-CN62
acuminatus-SMG-CN61
acuminatus-SMG-CN60
acuminatus-ML-CY74
acuminatus-ML-reference
acuminatus-LD-CY55
acuminatus-LD-CY56
acuminatus-LD-CY61
acuminatus-PM-CY48
acuminatus-PM-CY45
acuminatus-PM-CY47
acuminatus-PB-CY37
acuminatus-PB-CY35
acuminatus-PB-CY31
acuminatus-PB-CY36
acuminatus-PB-CY33
acuminatus-PB-CY34
acuminatus-SBZ-CY84
acuminatus-LD-CN45
acuminatus-BJP-CY15
acuminatus-BJP-CY14
acuminatus-BJP-CY76
acuminatus-CV-VL11
acuminatus-KKN-VL46
acuminatus-KKN-VL36
acuminatus-KKN-VL40
acuminatus-KKN-VL42
acuminatus-KKN-VL38
acuminatus-KKN-VL39
acuminatus-KKN-VL35
acuminatus-KKN-VL34
acuminatus-KKL-VL49
acuminatus-KKL-LVL8
acuminatus-KKL-VL24
acuminatus-KKL-LVL9
acuminatus-KKL-LVL5
acuminatus-KKL-LVL7
acuminatus-KKH-VL33
acuminatus-KKH-VL27
acuminatus-KKH-VL26
acuminatus-KKH-VL25
acuminatus-KKH-VL28
acuminatus-KKH-LVL10
acuminatus-KKH-VL50
bracteatus-BSY-CY67
bracteatus-BSY-CY70
bracteatus-LD-CY60
bracteatus-LD-CY57
bracteatus-SJS-CN23
bracteatus-MS-CY3
bracteatus-BSY-CY69
bracteatus-DWS-CY26
bracteatus-ADK-CY85
bracteatus-DWS-CY22
bracteatus-DWS-CY30
bracteatus-BSY-CY68
bracteatus-BJP-CY18
bracteatus-BJP-CY17
superbus-BLS-CY1
superbus-BLS-CY2
bracteatus-GLG-CY99
bracteatus-GLG-CY101
bracteatus-BHL-CY90
wardii-BHL-CY120
wardii-BHL-CY91
jouyi-XTBG-CY86
hookeri-BT-CY94

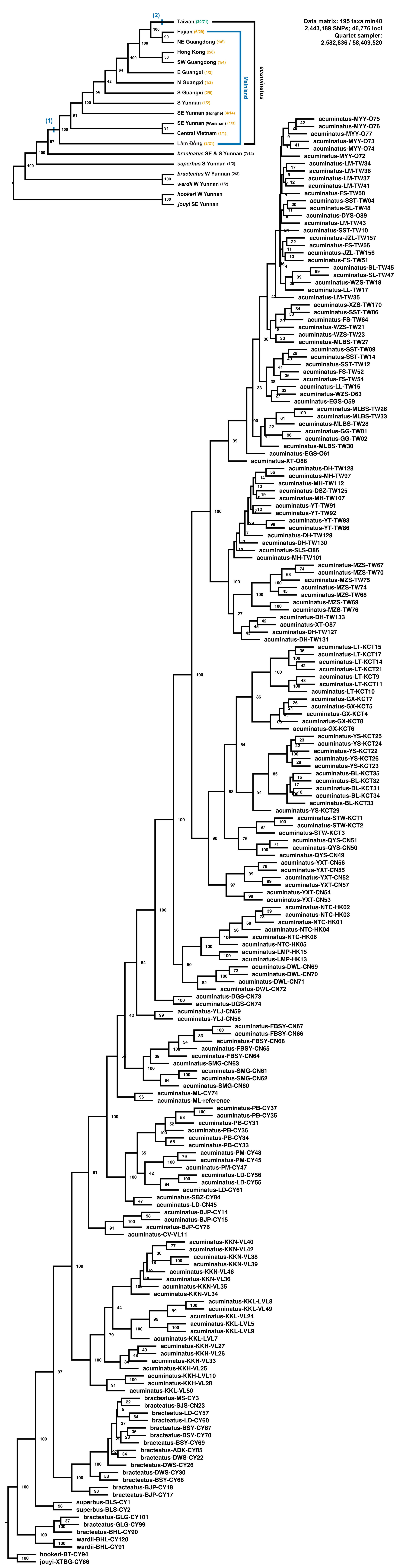

Data matrix: 195 taxa min40
2,443,189 SNPs; 46,776 loci
Quartet sampler:
2,582,836 / 58,409,520
(1)
(2)
Taiwan (20/71)
Fujian (6/29)
NE Guangdong (1/6)
Hong Kong (2/8)
SW Guangdong (1/4)
E Guangxi (1/2)
N Guangxi (1/2)
S Guangxi (2/9)
S Yunnan (1/2)
SE Yunnan (Honghe) (4/14)
SE Yunnan (Wenshan) (1/3)
Central Vietnam (1/1)
Lâm Đồng (3/21)
Mainland
acuminatus
bracteatus SE & S Yunnan (7/14)
superbus S Yunnan (1/2)
bracteatus W Yunnan (2/3)
wardii W Yunnan (1/2)
hookeri W Yunnan
jouyi SE Yunnan
acuminatus-MYY-O75
acuminatus-MYY-O76
acuminatus-MYY-O77
acuminatus-MYY-O73
acuminatus-MYY-O74
acuminatus-MYY-O72
acuminatus-LM-TW34
acuminatus-LM-TW36
acuminatus-LM-TW37
acuminatus-LM-TW41
acuminatus-FS-TW50
acuminatus-SST-TW04
acuminatus-SL-TW48
acuminatus-DYS-O89
acuminatus-LM-TW43
acuminatus-SST-TW10
acuminatus-JZL-TW157
acuminatus-FS-TW56
acuminatus-JZL-TW156
acuminatus-FS-TW51
acuminatus-SL-TW45
acuminatus-SL-TW47
acuminatus-WZS-TW18
acuminatus-LL-TW17
acuminatus-LM-TW35
acuminatus-XZS-TW170
acuminatus-SST-TW06
acuminatus-FS-TW64
acuminatus-WZS-TW21
acuminatus-WZS-TW23
acuminatus-MLBS-TW27
acuminatus-SST-TW09
acuminatus-SST-TW14
acuminatus-SST-TW12
acuminatus-FS-TW52
acuminatus-FS-TW54
acuminatus-LL-TW15
acuminatus-WZS-O63
acuminatus-EGS-O59
acuminatus-MLBS-TW26
acuminatus-MLBS-TW33
acuminatus-MLBS-TW28
acuminatus-GG-TW01
acuminatus-GG-TW02
acuminatus-MLBS-TW30
acuminatus-EGS-O61
acuminatus-XT-O88
acuminatus-DH-TW128
acuminatus-MH-TW97
acuminatus-MH-TW112
acuminatus-DSZ-TW125
acuminatus-MH-TW107
acuminatus-YT-TW91
acuminatus-YT-TW92
acuminatus-YT-TW83
acuminatus-YT-TW86
acuminatus-DH-TW129
acuminatus-DH-TW130
acuminatus-SLS-O86
acuminatus-MH-TW101
acuminatus-MZS-TW67
acuminatus-MZS-TW70
acuminatus-MZS-TW75
acuminatus-MZS-TW74
acuminatus-MZS-TW68
acuminatus-MZS-TW69
acuminatus-MZS-TW76
acuminatus-DH-TW133
acuminatus-XT-O87
acuminatus-DH-TW127
acuminatus-DH-TW131
acuminatus-LT-KCT15
acuminatus-LT-KCT17
acuminatus-LT-KCT14
acuminatus-LT-KCT21
acuminatus-LT-KCT9
acuminatus-LT-KCT11
acuminatus-LT-KCT10
acuminatus-GX-KCT7
acuminatus-GX-KCT5
acuminatus-GX-KCT4
acuminatus-GX-KCT8
acuminatus-GX-KCT6
acuminatus-YS-KCT25
acuminatus-YS-KCT24
acuminatus-YS-KCT22
acuminatus-YS-KCT23
acuminatus-YS-KCT26
acuminatus-BL-KCT35
acuminatus-BL-KCT32
acuminatus-BL-KCT31
acuminatus-BL-KCT34
acuminatus-BL-KCT33
acuminatus-YS-KCT29
acuminatus-STW-KCT1
acuminatus-STW-KCT2
acuminatus-STW-KCT3
acuminatus-QYS-CN51
acuminatus-QYS-CN50
acuminatus-QYS-CN49
acuminatus-YXT-CN56
acuminatus-YXT-CN55
acuminatus-YXT-CN52
acuminatus-YXT-CN57
acuminatus-YXT-CN54
acuminatus-YXT-CN53
acuminatus-NTC-HK02
acuminatus-NTC-HK03
acuminatus-NTC-HK01
acuminatus-NTC-HK04
acuminatus-NTC-HK06
acuminatus-NTC-HK05
acuminatus-LMP-HK15
acuminatus-LMP-HK13
acuminatus-DWL-CN69
acuminatus-DWL-CN70
acuminatus-DWL-CN71
acuminatus-DWL-CN72
acuminatus-DGS-CN73
acuminatus-DGS-CN74
acuminatus-YLJ-CN59
acuminatus-YLJ-CN58
acuminatus-FBSY-CN67
acuminatus-FBSY-CN66
acuminatus-FBSY-CN68
acuminatus-FBSY-CN65
acuminatus-FBSY-CN64
acuminatus-SMG-CN63
acuminatus-SMG-CN61
acuminatus-SMG-CN62
acuminatus-SMG-CN60
acuminatus-ML-CY74
acuminatus-ML-reference
acuminatus-PB-CY37
acuminatus-PB-CY35
acuminatus-PB-CY31
acuminatus-PB-CY36
acuminatus-PB-CY34
acuminatus-PB-CY33
acuminatus-PM-CY48
acuminatus-PM-CY45
acuminatus-PM-CY47
acuminatus-LD-CY56
acuminatus-LD-CY55
acuminatus-LD-CY61
acuminatus-SBZ-CY84
acuminatus-LD-CN45
acuminatus-BJP-CY14
acuminatus-BJP-CY15
acuminatus-BJP-CY76
acuminatus-CV-VL11
acuminatus-KKN-VL40
acuminatus-KKN-VL42
acuminatus-KKN-VL38
acuminatus-KKN-VL39
acuminatus-KKN-VL46
acuminatus-KKN-VL36
acuminatus-KKN-VL35
acuminatus-KKN-VL34
acuminatus-KKL-LVL8
acuminatus-KKL-VL49
acuminatus-KKL-VL24
acuminatus-KKL-LVL5
acuminatus-KKL-LVL9
acuminatus-KKL-LVL7
acuminatus-KKH-VL27
acuminatus-KKH-VL26
acuminatus-KKH-VL33
acuminatus-KKH-VL25
acuminatus-KKH-LVL10
acuminatus-KKH-VL28
acuminatus-KKL-VL50
bracteatus-MS-CY3
bracteatus-SJS-CN23
bracteatus-LD-CY57
bracteatus-LD-CY60
bracteatus-BSY-CY67
bracteatus-BSY-CY70
bracteatus-BSY-CY69
bracteatus-ADK-CY85
bracteatus-DWS-CY22
bracteatus-DWS-CY26
bracteatus-DWS-CY30
bracteatus-BSY-CY68
bracteatus-BJP-CY18
bracteatus-BJP-CY17
superbus-BLS-CY1
superbus-BLS-CY2
bracteatus-GLG-CY101
bracteatus-GLG-CY99
bracteatus-BHL-CY90
wardii-BHL-CY120
wardii-BHL-CY91
hookeri-BT-CY94
jouyi-XTBG-CY86

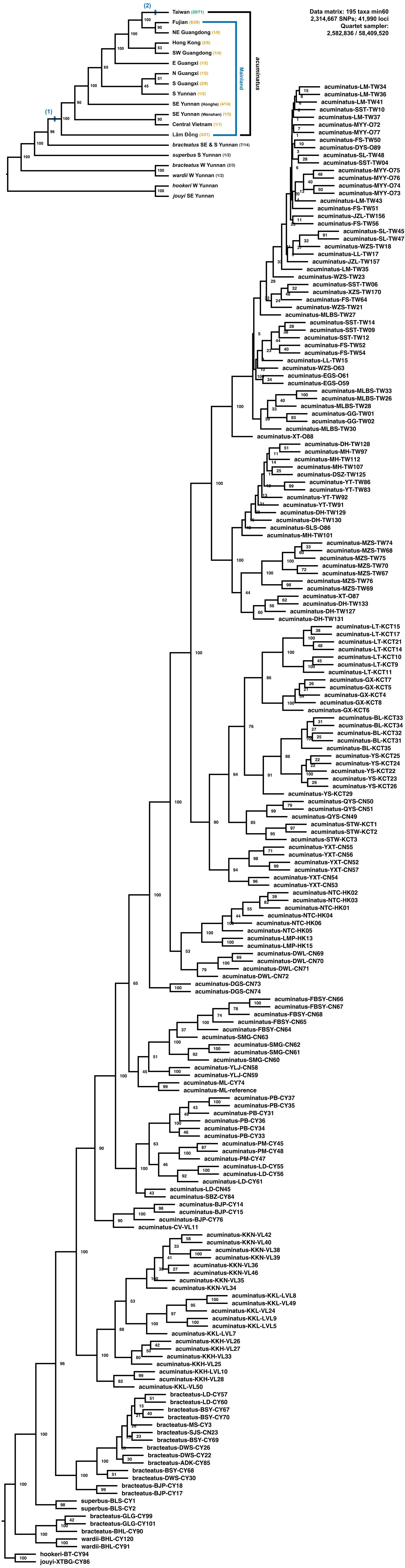
Data matrix: 195 taxa min60
2,314,667 SNPs; 41,990 loci
Quartet sampler:
2,582,836 / 58,409,520
acuminatus
Mainland

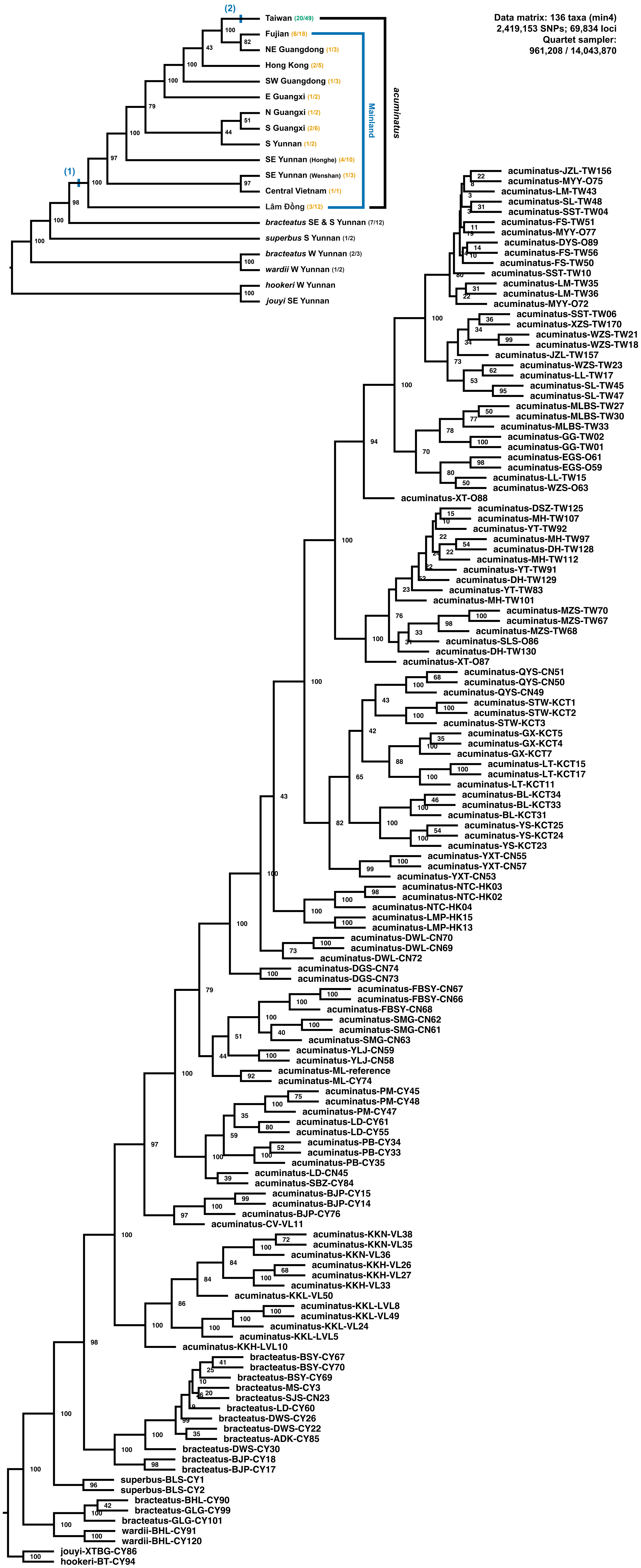
Data matrix: 136 taxa (min4)
2,419,153 SNPs; 69,834 loci
Quartet sampler:
961,208 / 14,043,870
(1)
(2)
Taiwan (20/49)
Fujian (6/18)
NE Guangdong (1/3)
Hong Kong (2/5)
SW Guangdong (1/3)
E Guangxi (1/2)
N Guangxi (1/2)
S Guangxi (2/6)
S Yunnan (1/2)
SE Yunnan (Honghe) (4/10)
SE Yunnan (Wenshan) (1/3)
Central Vietnam (1/1)
Lâm Đồng (3/12)
bracteatus SE & S Yunnan (7/12)
superbus S Yunnan (1/2)
bracteatus W Yunnan (2/3)
wardii W Yunnan (1/2)
hookeri W Yunnan
jouyi SE Yunnan
Mainland
acuminatus
acuminatus-JZL-TW156
acuminatus-MYY-O75
acuminatus-LM-TW43
acuminatus-SL-TW48
acuminatus-SST-TW04
acuminatus-FS-TW51
acuminatus-MYY-O77
acuminatus-DYS-O89
acuminatus-FS-TW56
acuminatus-FS-TW50
acuminatus-SST-TW10
acuminatus-LM-TW35
acuminatus-LM-TW36
acuminatus-MYY-O72
acuminatus-SST-TW06
acuminatus-XZS-TW170
acuminatus-WZS-TW21
acuminatus-WZS-TW18
acuminatus-JZL-TW157
acuminatus-WZS-TW23
acuminatus-LL-TW17
acuminatus-SL-TW45
acuminatus-SL-TW47
acuminatus-MLBS-TW27
acuminatus-MLBS-TW30
acuminatus-MLBS-TW33
acuminatus-GG-TW02
acuminatus-GG-TW01
acuminatus-EGS-O61
acuminatus-EGS-O59
acuminatus-LL-TW15
acuminatus-WZS-O63
acuminatus-XT-O88
acuminatus-DSZ-TW125
acuminatus-MH-TW107
acuminatus-YT-TW92
acuminatus-MH-TW97
acuminatus-DH-TW128
acuminatus-MH-TW112
acuminatus-YT-TW91
acuminatus-DH-TW129
acuminatus-YT-TW83
acuminatus-MH-TW101
acuminatus-MZS-TW70
acuminatus-MZS-TW67
acuminatus-MZS-TW68
acuminatus-SLS-O86
acuminatus-DH-TW130
acuminatus-XT-O87
acuminatus-QYS-CN51
acuminatus-QYS-CN50
acuminatus-QYS-CN49
acuminatus-STW-KCT1
acuminatus-STW-KCT2
acuminatus-STW-KCT3
acuminatus-GX-KCT5
acuminatus-GX-KCT4
acuminatus-GX-KCT7
acuminatus-LT-KCT15
acuminatus-LT-KCT17
acuminatus-LT-KCT11
acuminatus-BL-KCT34
acuminatus-BL-KCT33
acuminatus-BL-KCT31
acuminatus-YS-KCT25
acuminatus-YS-KCT24
acuminatus-YS-KCT23
acuminatus-YXT-CN55
acuminatus-YXT-CN57
acuminatus-YXT-CN53
acuminatus-NTC-HK03
acuminatus-NTC-HK02
acuminatus-NTC-HK04
acuminatus-LMP-HK15
acuminatus-LMP-HK13
acuminatus-DWL-CN70
acuminatus-DWL-CN69
acuminatus-DWL-CN72
acuminatus-DGS-CN74
acuminatus-DGS-CN73
acuminatus-FBSY-CN67
acuminatus-FBSY-CN66
acuminatus-FBSY-CN68
acuminatus-SMG-CN62
acuminatus-SMG-CN61
acuminatus-SMG-CN63
acuminatus-YLJ-CN59
acuminatus-YLJ-CN58
acuminatus-ML-reference
acuminatus-ML-CY74
acuminatus-PM-CY45
acuminatus-PM-CY48
acuminatus-PM-CY47
acuminatus-LD-CY61
acuminatus-LD-CY55
acuminatus-PB-CY34
acuminatus-PB-CY33
acuminatus-PB-CY35
acuminatus-LD-CN45
acuminatus-SBZ-CY84
acuminatus-BJP-CY15
acuminatus-BJP-CY14
acuminatus-BJP-CY76
acuminatus-CV-VL11
acuminatus-KKN-VL38
acuminatus-KKN-VL35
acuminatus-KKN-VL36
acuminatus-KKH-VL26
acuminatus-KKH-VL27
acuminatus-KKH-VL33
acuminatus-KKL-VL50
acuminatus-KKL-LVL8
acuminatus-KKL-VL49
acuminatus-KKL-VL24
acuminatus-KKL-LVL5
acuminatus-KKH-LVL10
bracteatus-BSY-CY67
bracteatus-BSY-CY70
bracteatus-BSY-CY69
bracteatus-MS-CY3
bracteatus-SJS-CN23
bracteatus-LD-CY60
bracteatus-DWS-CY26
bracteatus-DWS-CY22
bracteatus-ADK-CY85
bracteatus-DWS-CY30
bracteatus-BJP-CY18
bracteatus-BJP-CY17
superbus-BLS-CY1
superbus-BLS-CY2
bracteatus-BHL-CY90
bracteatus-GLG-CY99
bracteatus-GLG-CY101
wardii-BHL-CY91
wardii-BHL-CY120
jouyi-XTBG-CY86
hookeri-BT-CY94

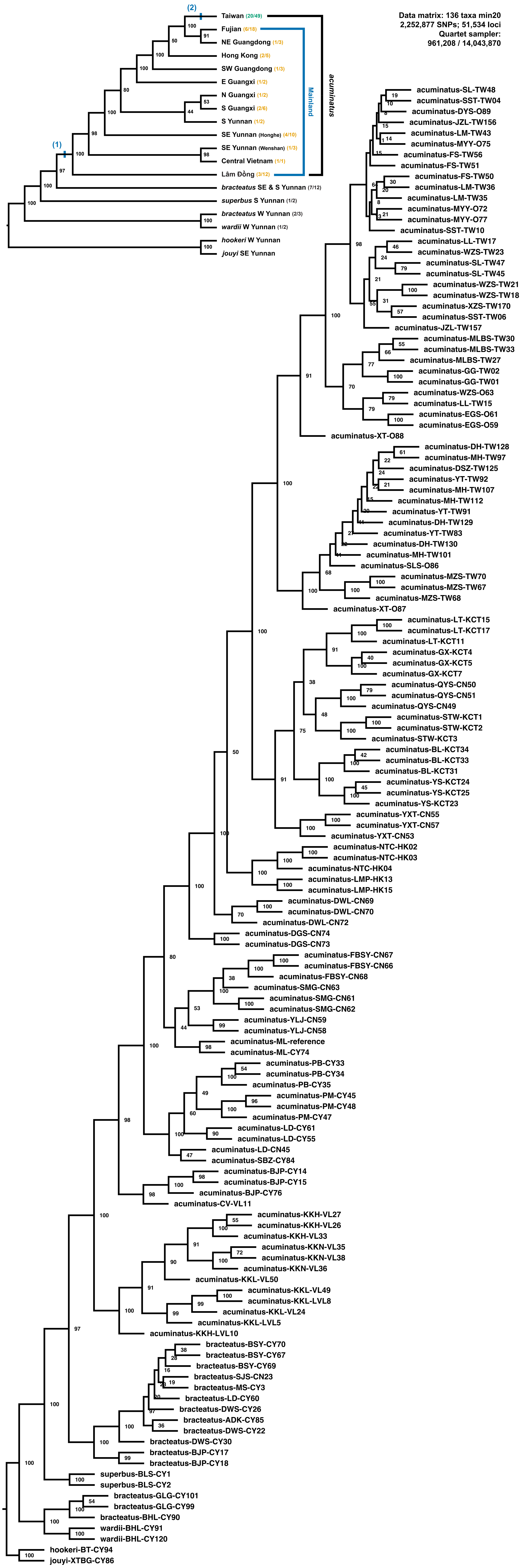
(2)
Taiwan (20/49)
Fujian (6/18)
NE Guangdong (1/3)
Hong Kong (2/5)
SW Guangdong (1/3)
E Guangxi (1/2)
N Guangxi (1/2)
S Guangxi (2/6)
S Yunnan (1/2)
SE Yunnan (Honghe) (4/10)
SE Yunnan (Wenshan) (1/3)
Central Vietnam (1/1)
Lâm Đồng (3/12)
(1)
bracteatus SE & S Yunnan (7/12)
superbus S Yunnan (1/2)
bracteatus W Yunnan (2/3)
wardii W Yunnan (1/2)
hookeri W Yunnan
jouyi SE Yunnan
Mainland
acuminatus
Data matrix: 136 taxa min20
2,252,877 SNPs; 51,534 loci
Quartet sampler:
961,208 / 14,043,870
acuminatus-SL-TW48
acuminatus-SST-TW04
acuminatus-DYS-O89
acuminatus-JZL-TW156
acuminatus-LM-TW43
acuminatus-MYY-O75
acuminatus-FS-TW56
acuminatus-FS-TW51
acuminatus-FS-TW50
acuminatus-LM-TW36
acuminatus-LM-TW35
acuminatus-MYY-O72
acuminatus-MYY-O77
acuminatus-SST-TW10
acuminatus-LL-TW17
acuminatus-WZS-TW23
acuminatus-SL-TW47
acuminatus-SL-TW45
acuminatus-WZS-TW21
acuminatus-WZS-TW18
acuminatus-XZS-TW170
acuminatus-SST-TW06
acuminatus-JZL-TW157
acuminatus-MLBS-TW30
acuminatus-MLBS-TW33
acuminatus-MLBS-TW27
acuminatus-GG-TW02
acuminatus-GG-TW01
acuminatus-WZS-O63
acuminatus-LL-TW15
acuminatus-EGS-O61
acuminatus-EGS-O59
acuminatus-XT-O88
acuminatus-DH-TW128
acuminatus-MH-TW97
acuminatus-DSZ-TW125
acuminatus-YT-TW92
acuminatus-MH-TW107
acuminatus-MH-TW112
acuminatus-YT-TW91
acuminatus-DH-TW129
acuminatus-YT-TW83
acuminatus-DH-TW130
acuminatus-MH-TW101
acuminatus-SLS-O86
acuminatus-MZS-TW70
acuminatus-MZS-TW67
acuminatus-MZS-TW68
acuminatus-XT-O87
acuminatus-LT-KCT15
acuminatus-LT-KCT17
acuminatus-LT-KCT11
acuminatus-GX-KCT4
acuminatus-GX-KCT5
acuminatus-GX-KCT7
acuminatus-QYS-CN50
acuminatus-QYS-CN51
acuminatus-QYS-CN49
acuminatus-STW-KCT1
acuminatus-STW-KCT2
acuminatus-STW-KCT3
acuminatus-BL-KCT34
acuminatus-BL-KCT33
acuminatus-BL-KCT31
acuminatus-YS-KCT24
acuminatus-YS-KCT25
acuminatus-YS-KCT23
acuminatus-YXT-CN55
acuminatus-YXT-CN57
acuminatus-YXT-CN53
acuminatus-NTC-HK02
acuminatus-NTC-HK03
acuminatus-NTC-HK04
acuminatus-LMP-HK13
acuminatus-LMP-HK15
acuminatus-DWL-CN69
acuminatus-DWL-CN70
acuminatus-DWL-CN72
acuminatus-DGS-CN74
acuminatus-DGS-CN73
acuminatus-FBSY-CN67
acuminatus-FBSY-CN66
acuminatus-FBSY-CN68
acuminatus-SMG-CN63
acuminatus-SMG-CN61
acuminatus-SMG-CN62
acuminatus-YLJ-CN59
acuminatus-YLJ-CN58
acuminatus-ML-reference
acuminatus-ML-CY74
acuminatus-PB-CY33
acuminatus-PB-CY34
acuminatus-PB-CY35
acuminatus-PM-CY45
acuminatus-PM-CY48
acuminatus-PM-CY47
acuminatus-LD-CY61
acuminatus-LD-CY55
acuminatus-LD-CN45
acuminatus-SBZ-CY84
acuminatus-BJP-CY14
acuminatus-BJP-CY15
acuminatus-BJP-CY76
acuminatus-CV-VL11
acuminatus-KKH-VL27
acuminatus-KKH-VL26
acuminatus-KKH-VL33
acuminatus-KKN-VL35
acuminatus-KKN-VL38
acuminatus-KKN-VL36
acuminatus-KKL-VL50
acuminatus-KKL-VL49
acuminatus-KKL-LVL8
acuminatus-KKL-VL24
acuminatus-KKL-LVL5
acuminatus-KKH-LVL10
bracteatus-BSY-CY70
bracteatus-BSY-CY67
bracteatus-BSY-CY69
bracteatus-SJS-CN23
bracteatus-MS-CY3
bracteatus-LD-CY60
bracteatus-DWS-CY26
bracteatus-ADK-CY85
bracteatus-DWS-CY22
bracteatus-DWS-CY30
bracteatus-BJP-CY17
bracteatus-BJP-CY18
superbus-BLS-CY1
superbus-BLS-CY2
bracteatus-GLG-CY101
bracteatus-GLG-CY99
bracteatus-BHL-CY90
wardii-BHL-CY91
wardii-BHL-CY120
hookeri-BT-CY94
jouyi-XTBG-CY86

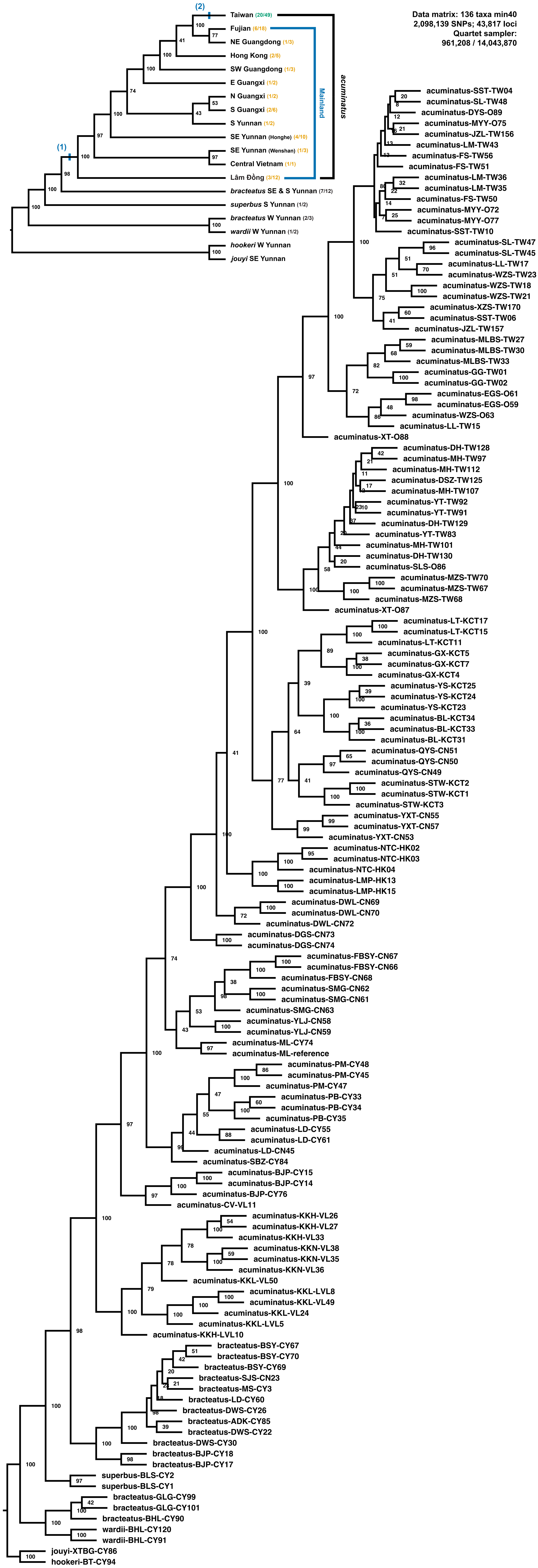

Data matrix: 136 taxa min40
2,098,139 SNPs; 43,817 loci
Quartet sampler:
961,208 / 14,043,870
(1)
(2)
acuminatus
Mainland
Taiwan (20/49)
Fujian (6/18)
NE Guangdong (1/3)
Hong Kong (2/5)
SW Guangdong (1/3)
E Guangxi (1/2)
N Guangxi (1/2)
S Guangxi (2/6)
S Yunnan (1/2)
SE Yunnan (Honghe) (4/10)
SE Yunnan (Wenshan) (1/3)
Central Vietnam (1/1)
Lâm Đồng (3/12)
bracteatus SE & S Yunnan (7/12)
superbus S Yunnan (1/2)
bracteatus W Yunnan (2/3)
wardii W Yunnan (1/2)
hookeri W Yunnan
jouyi SE Yunnan
acuminatus-SST-TW04
acuminatus-SL-TW48
acuminatus-DYS-O89
acuminatus-MYY-O75
acuminatus-JZL-TW156
acuminatus-LM-TW43
acuminatus-FS-TW56
acuminatus-FS-TW51
acuminatus-LM-TW36
acuminatus-LM-TW35
acuminatus-FS-TW50
acuminatus-MYY-O72
acuminatus-MYY-O77
acuminatus-SST-TW10
acuminatus-SL-TW47
acuminatus-SL-TW45
acuminatus-LL-TW17
acuminatus-WZS-TW23
acuminatus-WZS-TW18
acuminatus-WZS-TW21
acuminatus-XZS-TW170
acuminatus-SST-TW06
acuminatus-JZL-TW157
acuminatus-MLBS-TW27
acuminatus-MLBS-TW30
acuminatus-MLBS-TW33
acuminatus-GG-TW01
acuminatus-GG-TW02
acuminatus-EGS-O61
acuminatus-EGS-O59
acuminatus-WZS-O63
acuminatus-LL-TW15
acuminatus-XT-O88
acuminatus-DH-TW128
acuminatus-MH-TW97
acuminatus-MH-TW112
acuminatus-DSZ-TW125
acuminatus-MH-TW107
acuminatus-YT-TW92
acuminatus-YT-TW91
acuminatus-DH-TW129
acuminatus-YT-TW83
acuminatus-MH-TW101
acuminatus-DH-TW130
acuminatus-SLS-O86
acuminatus-MZS-TW70
acuminatus-MZS-TW67
acuminatus-MZS-TW68
acuminatus-XT-O87
acuminatus-LT-KCT17
acuminatus-LT-KCT15
acuminatus-LT-KCT11
acuminatus-GX-KCT5
acuminatus-GX-KCT7
acuminatus-GX-KCT4
acuminatus-YS-KCT25
acuminatus-YS-KCT24
acuminatus-YS-KCT23
acuminatus-BL-KCT34
acuminatus-BL-KCT33
acuminatus-BL-KCT31
acuminatus-QYS-CN51
acuminatus-QYS-CN50
acuminatus-QYS-CN49
acuminatus-STW-KCT2
acuminatus-STW-KCT1
acuminatus-STW-KCT3
acuminatus-YXT-CN55
acuminatus-YXT-CN57
acuminatus-YXT-CN53
acuminatus-NTC-HK02
acuminatus-NTC-HK03
acuminatus-NTC-HK04
acuminatus-LMP-HK13
acuminatus-LMP-HK15
acuminatus-DWL-CN69
acuminatus-DWL-CN70
acuminatus-DWL-CN72
acuminatus-DGS-CN73
acuminatus-DGS-CN74
acuminatus-FBSY-CN67
acuminatus-FBSY-CN66
acuminatus-FBSY-CN68
acuminatus-SMG-CN62
acuminatus-SMG-CN61
acuminatus-SMG-CN63
acuminatus-YLJ-CN58
acuminatus-YLJ-CN59
acuminatus-ML-CY74
acuminatus-ML-reference
acuminatus-PM-CY48
acuminatus-PM-CY45
acuminatus-PM-CY47
acuminatus-PB-CY33
acuminatus-PB-CY34
acuminatus-PB-CY35
acuminatus-LD-CY55
acuminatus-LD-CY61
acuminatus-LD-CN45
acuminatus-SBZ-CY84
acuminatus-BJP-CY15
acuminatus-BJP-CY14
acuminatus-BJP-CY76
acuminatus-CV-VL11
acuminatus-KKH-VL26
acuminatus-KKH-VL27
acuminatus-KKH-VL33
acuminatus-KKN-VL38
acuminatus-KKN-VL35
acuminatus-KKN-VL36
acuminatus-KKL-VL50
acuminatus-KKL-LVL8
acuminatus-KKL-VL49
acuminatus-KKL-VL24
acuminatus-KKL-LVL5
acuminatus-KKH-LVL10
bracteatus-BSY-CY67
bracteatus-BSY-CY70
bracteatus-BSY-CY69
bracteatus-SJS-CN23
bracteatus-MS-CY3
bracteatus-LD-CY60
bracteatus-DWS-CY26
bracteatus-ADK-CY85
bracteatus-DWS-CY22
bracteatus-DWS-CY30
bracteatus-BJP-CY18
bracteatus-BJP-CY17
superbus-BLS-CY2
superbus-BLS-CY1
bracteatus-GLG-CY99
bracteatus-GLG-CY101
bracteatus-BHL-CY90
wardii-BHL-CY120
wardii-BHL-CY91
jouyi-XTBG-CY86
hookeri-BT-CY94

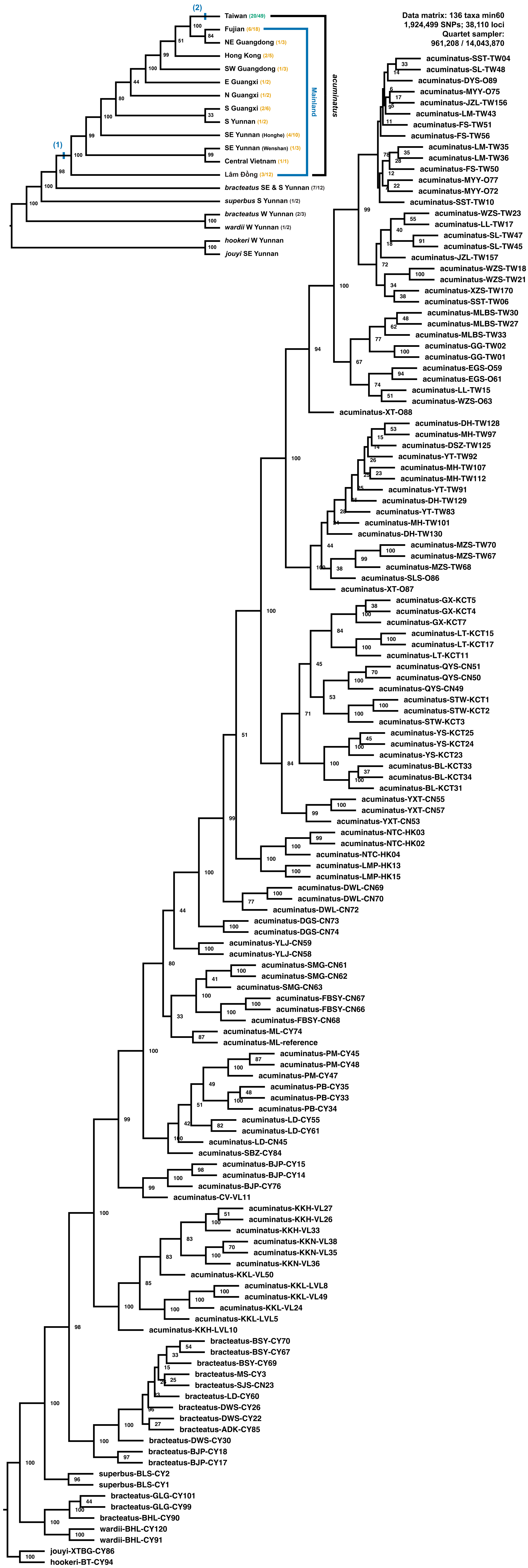

(1)
(2)
Taiwan (20/49)
Fujian (6/18)
NE Guangdong (1/3)
Hong Kong (2/5)
SW Guangdong (1/3)
E Guangxi (1/2)
N Guangxi (1/2)
S Guangxi (2/6)
S Yunnan (1/2)
SE Yunnan (Honghe) (4/10)
SE Yunnan (Wenshan) (1/3)
Central Vietnam (1/1)
Lâm Đồng (3/12)
Mainland
acuminatus
bracteatus SE & S Yunnan (7/12)
superbus S Yunnan (1/2)
bracteatus W Yunnan (2/3)
wardii W Yunnan (1/2)
hookeri W Yunnan
jouyi SE Yunnan
Data matrix: 136 taxa min60
1,924,499 SNPs; 38,110 loci
Quartet sampler:
961,208 / 14,043,870
acuminatus-SST-TW04
acuminatus-SL-TW48
acuminatus-DYS-O89
acuminatus-MYY-O75
acuminatus-JZL-TW156
acuminatus-LM-TW43
acuminatus-FS-TW51
acuminatus-FS-TW56
acuminatus-LM-TW35
acuminatus-LM-TW36
acuminatus-FS-TW50
acuminatus-MYY-O77
acuminatus-MYY-O72
acuminatus-SST-TW10
acuminatus-WZS-TW23
acuminatus-LL-TW17
acuminatus-SL-TW47
acuminatus-SL-TW45
acuminatus-JZL-TW157
acuminatus-WZS-TW18
acuminatus-WZS-TW21
acuminatus-XZS-TW170
acuminatus-SST-TW06
acuminatus-MLBS-TW30
acuminatus-MLBS-TW27
acuminatus-MLBS-TW33
acuminatus-GG-TW02
acuminatus-GG-TW01
acuminatus-EGS-O59
acuminatus-EGS-O61
acuminatus-LL-TW15
acuminatus-WZS-O63
acuminatus-XT-O88
acuminatus-DH-TW128
acuminatus-MH-TW97
acuminatus-DSZ-TW125
acuminatus-YT-TW92
acuminatus-MH-TW107
acuminatus-MH-TW112
acuminatus-YT-TW91
acuminatus-DH-TW129
acuminatus-YT-TW83
acuminatus-MH-TW101
acuminatus-DH-TW130
acuminatus-MZS-TW70
acuminatus-MZS-TW67
acuminatus-MZS-TW68
acuminatus-SLS-O86
acuminatus-XT-O87
acuminatus-GX-KCT5
acuminatus-GX-KCT4
acuminatus-GX-KCT7
acuminatus-LT-KCT15
acuminatus-LT-KCT17
acuminatus-LT-KCT11
acuminatus-QYS-CN51
acuminatus-QYS-CN50
acuminatus-QYS-CN49
acuminatus-STW-KCT1
acuminatus-STW-KCT2
acuminatus-STW-KCT3
acuminatus-YS-KCT25
acuminatus-YS-KCT24
acuminatus-YS-KCT23
acuminatus-BL-KCT33
acuminatus-BL-KCT34
acuminatus-BL-KCT31
acuminatus-YXT-CN55
acuminatus-YXT-CN57
acuminatus-YXT-CN53
acuminatus-NTC-HK03
acuminatus-NTC-HK02
acuminatus-NTC-HK04
acuminatus-LMP-HK13
acuminatus-LMP-HK15
acuminatus-DWL-CN69
acuminatus-DWL-CN70
acuminatus-DWL-CN72
acuminatus-DGS-CN73
acuminatus-DGS-CN74
acuminatus-YLJ-CN59
acuminatus-YLJ-CN58
acuminatus-SMG-CN61
acuminatus-SMG-CN62
acuminatus-SMG-CN63
acuminatus-FBSY-CN67
acuminatus-FBSY-CN66
acuminatus-FBSY-CN68
acuminatus-ML-CY74
acuminatus-ML-reference
acuminatus-PM-CY45
acuminatus-PM-CY48
acuminatus-PM-CY47
acuminatus-PB-CY35
acuminatus-PB-CY33
acuminatus-PB-CY34
acuminatus-LD-CY55
acuminatus-LD-CY61
acuminatus-LD-CN45
acuminatus-SBZ-CY84
acuminatus-BJP-CY15
acuminatus-BJP-CY14
acuminatus-BJP-CY76
acuminatus-CV-VL11
acuminatus-KKH-VL27
acuminatus-KKH-VL26
acuminatus-KKH-VL33
acuminatus-KKN-VL38
acuminatus-KKN-VL35
acuminatus-KKN-VL36
acuminatus-KKL-VL50
acuminatus-KKL-LVL8
acuminatus-KKL-VL49
acuminatus-KKL-VL24
acuminatus-KKL-LVL5
acuminatus-KKH-LVL10
bracteatus-BSY-CY70
bracteatus-BSY-CY67
bracteatus-BSY-CY69
bracteatus-MS-CY3
bracteatus-SJS-CN23
bracteatus-LD-CY60
bracteatus-DWS-CY26
bracteatus-DWS-CY22
bracteatus-ADK-CY85
bracteatus-DWS-CY30
bracteatus-BJP-CY18
bracteatus-BJP-CY17
superbus-BLS-CY2
superbus-BLS-CY1
bracteatus-GLG-CY101
bracteatus-GLG-CY99
bracteatus-BHL-CY90
wardii-BHL-CY120
wardii-BHL-CY91
jouyi-XTBG-CY86
hookeri-BT-CY94
