# Supplementary material for: Testing macroevolutionary predictions of the Grant‐Stebbins model in the origin of Aeschynanthus acuminatus
Source: New Phytol. 2026 Jan 27;249(6):3137–48. doi: 10.1111/nph.70871 (PMC12917478; doi:10.1111/nph.70871)
Supplement: Supplementary file 3 — Fig. S4 Maximum likelihood phylogenetic inference of Aeschynanthus acuminatus and related species using IQ‐TREE analyses based on concatenated SNP matrices of eight data sets. [file NPH-249-3137-s004.pdf]

New Phytologist Supporting Information

Article title: Testing macroevolutionary predictions of the Grant-Stebbins model in the origin of *Aeschynanthus acuminatus*
Authors: Jing-Yi Lu, Yaowu Xing, Hong Truong Luu, Richard H Ree
Article acceptance date: 4 December 2025

Figure S4. Maximum likelihood phylogenetic inference of *Aeschynanthus acuminatus* and related species using IQ-TREE analyses based on concatenated SNP matrices of eight data sets. Terminal branches represent monophyletic groups and are labeled by geographic location and the number of sampled populations and individuals. Node labels show bootstrap values. Vertical bars on branches mark key events: (1) Speciation of *A. acuminatus* on the mainland and (2) colonization of Taiwan.

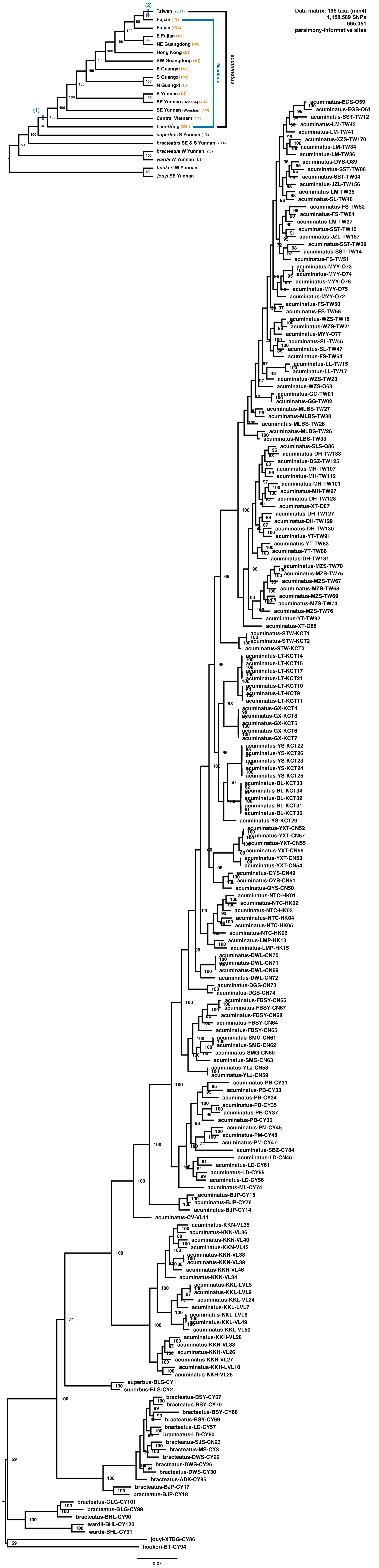

Data matrix: 195 taxa (min4)
1,158,589 SNPs
665,051
parsimony-informative sites
(1)
(2)
Taiwan (20/71)
Fujian (1/3)
Fujian (4/23)
E Fujian (1/3)
NE Guangdong (1/6)
Hong Kong (2/8)
SW Guangdong (1/4)
E Guangxi (1/2)
S Guangxi (2/9)
N Guangxi (1/2)
S Yunnan (1/1)
SE Yunnan (Honghe) (4/14)
SE Yunnan (Wenshan) (1/3)
Central Vietnam (1/1)
Lâm Đồng (3/21)
superbus S Yunnan (1/2)
bracteatus SE & S Yunnan (7/14)
bracteatus W Yunnan (2/3)
wardii W Yunnan (1/2)
hookeri W Yunnan
jouyi SE Yunnan
Mainland
acuminatus
acuminatus-EGS-O59
acuminatus-EGS-O61
acuminatus-SST-TW12
acuminatus-LM-TW43
acuminatus-LM-TW41
acuminatus-XZS-TW170
acuminatus-LM-TW34
acuminatus-LM-TW36
acuminatus-DYS-O89
acuminatus-SST-TW06
acuminatus-SST-TW04
acuminatus-JZL-TW156
acuminatus-LM-TW35
acuminatus-SL-TW48
acuminatus-FS-TW52
acuminatus-FS-TW64
acuminatus-LM-TW37
acuminatus-SST-TW10
acuminatus-JZL-TW157
acuminatus-SST-TW09
acuminatus-SST-TW14
acuminatus-FS-TW51
acuminatus-MYY-O73
acuminatus-MYY-O74
acuminatus-MYY-O76
acuminatus-MYY-O75
acuminatus-MYY-O72
acuminatus-FS-TW50
acuminatus-FS-TW56
acuminatus-WZS-TW18
acuminatus-WZS-TW21
acuminatus-MYY-O77
acuminatus-SL-TW45
acuminatus-SL-TW47
acuminatus-FS-TW54
acuminatus-LL-TW15
acuminatus-LL-TW17
acuminatus-WZS-TW23
acuminatus-WZS-O63
acuminatus-GG-TW01
acuminatus-GG-TW02
acuminatus-MLBS-TW27
acuminatus-MLBS-TW30
acuminatus-MLBS-TW28
acuminatus-MLBS-TW26
acuminatus-MLBS-TW33
acuminatus-SLS-O86
acuminatus-DH-TW133
acuminatus-DSZ-TW125
acuminatus-MH-TW107
acuminatus-MH-TW112
acuminatus-MH-TW101
acuminatus-MH-TW97
acuminatus-DH-TW128
acuminatus-XT-O87
acuminatus-DH-TW127
acuminatus-DH-TW129
acuminatus-DH-TW130
acuminatus-YT-TW91
acuminatus-YT-TW83
acuminatus-YT-TW86
acuminatus-DH-TW131
acuminatus-MZS-TW70
acuminatus-MZS-TW75
acuminatus-MZS-TW67
acuminatus-MZS-TW68
acuminatus-MZS-TW69
acuminatus-MZS-TW74
acuminatus-MZS-TW76
acuminatus-YT-TW92
acuminatus-XT-O88
acuminatus-STW-KCT1
acuminatus-STW-KCT2
acuminatus-STW-KCT3
acuminatus-LT-KCT14
acuminatus-LT-KCT15
acuminatus-LT-KCT17
acuminatus-LT-KCT21
acuminatus-LT-KCT10
acuminatus-LT-KCT9
acuminatus-LT-KCT11
acuminatus-GX-KCT4
acuminatus-GX-KCT8
acuminatus-GX-KCT5
acuminatus-GX-KCT6
acuminatus-GX-KCT7
acuminatus-YS-KCT22
acuminatus-YS-KCT26
acuminatus-YS-KCT23
acuminatus-YS-KCT24
acuminatus-YS-KCT25
acuminatus-BL-KCT33
acuminatus-BL-KCT34
acuminatus-BL-KCT32
acuminatus-BL-KCT31
acuminatus-BL-KCT35
acuminatus-YS-KCT29
acuminatus-YXT-CN52
acuminatus-YXT-CN57
acuminatus-YXT-CN55
acuminatus-YXT-CN56
acuminatus-YXT-CN53
acuminatus-YXT-CN54
acuminatus-QYS-CN49
acuminatus-QYS-CN51
acuminatus-QYS-CN50
acuminatus-NTC-HK01
acuminatus-NTC-HK02
acuminatus-NTC-HK03
acuminatus-NTC-HK04
acuminatus-NTC-HK05
acuminatus-NTC-HK06
acuminatus-LMP-HK13
acuminatus-LMP-HK15
acuminatus-DWL-CN70
acuminatus-DWL-CN71
acuminatus-DWL-CN69
acuminatus-DWL-CN72
acuminatus-DGS-CN73
acuminatus-DGS-CN74
acuminatus-FBSY-CN66
acuminatus-FBSY-CN67
acuminatus-FBSY-CN68
acuminatus-FBSY-CN64
acuminatus-FBSY-CN65
acuminatus-SMG-CN61
acuminatus-SMG-CN62
acuminatus-SMG-CN60
acuminatus-SMG-CN63
acuminatus-YLJ-CN58
acuminatus-YLJ-CN59
acuminatus-PB-CY31
acuminatus-PB-CY33
acuminatus-PB-CY34
acuminatus-PB-CY35
acuminatus-PB-CY37
acuminatus-PB-CY36
acuminatus-PM-CY45
acuminatus-PM-CY48
acuminatus-PM-CY47
acuminatus-SBZ-CY84
acuminatus-LD-CN45
acuminatus-LD-CY61
acuminatus-LD-CY55
acuminatus-LD-CY56
acuminatus-ML-CY74
acuminatus-BJP-CY15
acuminatus-BJP-CY76
acuminatus-BJP-CY14
acuminatus-CV-VL11
acuminatus-KKN-VL35
acuminatus-KKN-VL36
acuminatus-KKN-VL40
acuminatus-KKN-VL42
acuminatus-KKN-VL38
acuminatus-KKN-VL39
acuminatus-KKN-VL46
acuminatus-KKN-VL34
acuminatus-KKL-LVL5
acuminatus-KKL-LVL9
acuminatus-KKL-VL24
acuminatus-KKL-LVL7
acuminatus-KKL-LVL8
acuminatus-KKL-VL49
acuminatus-KKL-VL50
acuminatus-KKH-VL28
acuminatus-KKH-VL33
acuminatus-KKH-VL26
acuminatus-KKH-VL27
acuminatus-KKH-LVL10
acuminatus-KKH-VL25
superbus-BLS-CY1
superbus-BLS-CY2
bracteatus-BSY-CY67
bracteatus-BSY-CY70
bracteatus-BSY-CY68
bracteatus-BSY-CY69
bracteatus-LD-CY57
bracteatus-LD-CY60
bracteatus-SJS-CN23
bracteatus-MS-CY3
bracteatus-DWS-CY22
bracteatus-DWS-CY26
bracteatus-DWS-CY30
bracteatus-ADK-CY85
bracteatus-BJP-CY17
bracteatus-BJP-CY18
bracteatus-GLG-CY101
bracteatus-GLG-CY99
bracteatus-BHL-CY90
wardii-BHL-CY120
wardii-BHL-CY91
jouyi-XTBG-CY86
hookeri-BT-CY94
0.07

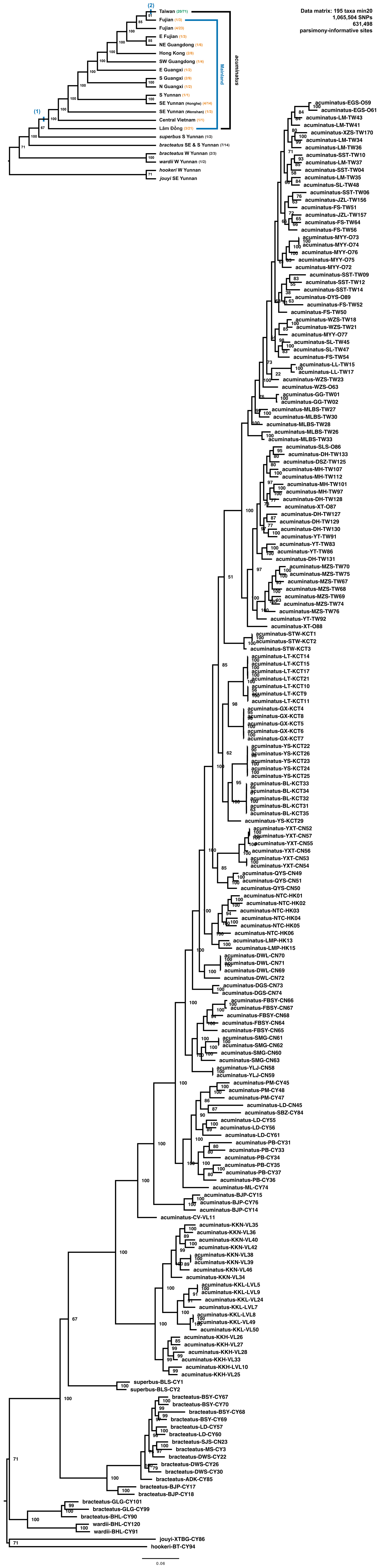

Data matrix: 195 taxa min20
1,065,504 SNPs
631,498
parsimony-informative sites
(1)
(2)
Taiwan (20/71)
Fujian (1/3)
Fujian (4/23)
E Fujian (1/2)
NE Guangdong (1/6)
Hong Kong (2/8)
SW Guangdong (1/4)
E Guangxi (1/2)
S Guangxi (2/9)
N Guangxi (1/2)
S Yunnan (1/1)
SE Yunnan (Honghe) (4/14)
SE Yunnan (Wenshan) (1/3)
Central Vietnam (1/1)
Lâm Đồng (3/21)
superbus S Yunnan (1/2)
bracteatus SE & S Yunnan (7/14)
bracteatus W Yunnan (2/3)
wardii W Yunnan (1/2)
hookeri W Yunnan
jouyi SE Yunnan
acuminatus
Mainland
acuminatus-EGS-O59
acuminatus-EGS-O61
acuminatus-LM-TW43
acuminatus-LM-TW41
acuminatus-XZS-TW170
acuminatus-LM-TW34
acuminatus-LM-TW36
acuminatus-SST-TW10
acuminatus-LM-TW37
acuminatus-SST-TW04
acuminatus-LM-TW35
acuminatus-SL-TW48
acuminatus-SST-TW06
acuminatus-JZL-TW156
acuminatus-FS-TW51
acuminatus-JZL-TW157
acuminatus-FS-TW64
acuminatus-FS-TW56
acuminatus-MYY-O73
acuminatus-MYY-O74
acuminatus-MYY-O76
acuminatus-MYY-O75
acuminatus-MYY-O72
acuminatus-SST-TW09
acuminatus-SST-TW12
acuminatus-SST-TW14
acuminatus-DYS-O89
acuminatus-FS-TW52
acuminatus-FS-TW50
acuminatus-WZS-TW18
acuminatus-WZS-TW21
acuminatus-MYY-O77
acuminatus-SL-TW45
acuminatus-SL-TW47
acuminatus-FS-TW54
acuminatus-LL-TW15
acuminatus-LL-TW17
acuminatus-WZS-TW23
acuminatus-WZS-O63
acuminatus-GG-TW01
acuminatus-GG-TW02
acuminatus-MLBS-TW27
acuminatus-MLBS-TW30
acuminatus-MLBS-TW28
acuminatus-MLBS-TW26
acuminatus-MLBS-TW33
acuminatus-SLS-O86
acuminatus-DH-TW133
acuminatus-DSZ-TW125
acuminatus-MH-TW107
acuminatus-MH-TW112
acuminatus-MH-TW101
acuminatus-MH-TW97
acuminatus-DH-TW128
acuminatus-XT-O87
acuminatus-DH-TW127
acuminatus-DH-TW129
acuminatus-DH-TW130
acuminatus-YT-TW91
acuminatus-YT-TW83
acuminatus-YT-TW86
acuminatus-DH-TW131
acuminatus-MZS-TW70
acuminatus-MZS-TW75
acuminatus-MZS-TW67
acuminatus-MZS-TW68
acuminatus-MZS-TW69
acuminatus-MZS-TW74
acuminatus-MZS-TW76
acuminatus-YT-TW92
acuminatus-XT-O88
acuminatus-STW-KCT1
acuminatus-STW-KCT2
acuminatus-STW-KCT3
acuminatus-LT-KCT14
acuminatus-LT-KCT15
acuminatus-LT-KCT17
acuminatus-LT-KCT21
acuminatus-LT-KCT10
acuminatus-LT-KCT9
acuminatus-LT-KCT11
acuminatus-GX-KCT4
acuminatus-GX-KCT8
acuminatus-GX-KCT5
acuminatus-GX-KCT6
acuminatus-GX-KCT7
acuminatus-YS-KCT22
acuminatus-YS-KCT26
acuminatus-YS-KCT23
acuminatus-YS-KCT24
acuminatus-YS-KCT25
acuminatus-BL-KCT33
acuminatus-BL-KCT34
acuminatus-BL-KCT32
acuminatus-BL-KCT31
acuminatus-BL-KCT35
acuminatus-YS-KCT29
acuminatus-YXT-CN52
acuminatus-YXT-CN57
acuminatus-YXT-CN55
acuminatus-YXT-CN56
acuminatus-YXT-CN53
acuminatus-YXT-CN54
acuminatus-QYS-CN49
acuminatus-QYS-CN51
acuminatus-QYS-CN50
acuminatus-NTC-HK01
acuminatus-NTC-HK02
acuminatus-NTC-HK03
acuminatus-NTC-HK04
acuminatus-NTC-HK05
acuminatus-NTC-HK06
acuminatus-LMP-HK13
acuminatus-LMP-HK15
acuminatus-DWL-CN70
acuminatus-DWL-CN71
acuminatus-DWL-CN69
acuminatus-DWL-CN72
acuminatus-DGS-CN73
acuminatus-DGS-CN74
acuminatus-FBSY-CN66
acuminatus-FBSY-CN67
acuminatus-FBSY-CN68
acuminatus-FBSY-CN64
acuminatus-FBSY-CN65
acuminatus-SMG-CN61
acuminatus-SMG-CN62
acuminatus-SMG-CN60
acuminatus-SMG-CN63
acuminatus-YLJ-CN58
acuminatus-YLJ-CN59
acuminatus-PM-CY45
acuminatus-PM-CY48
acuminatus-PM-CY47
acuminatus-LD-CN45
acuminatus-SBZ-CY84
acuminatus-LD-CY55
acuminatus-LD-CY56
acuminatus-LD-CY61
acuminatus-PB-CY31
acuminatus-PB-CY33
acuminatus-PB-CY34
acuminatus-PB-CY35
acuminatus-PB-CY37
acuminatus-PB-CY36
acuminatus-ML-CY74
acuminatus-BJP-CY15
acuminatus-BJP-CY76
acuminatus-BJP-CY14
acuminatus-CV-VL11
acuminatus-KKN-VL35
acuminatus-KKN-VL36
acuminatus-KKN-VL40
acuminatus-KKN-VL42
acuminatus-KKN-VL38
acuminatus-KKN-VL39
acuminatus-KKN-VL46
acuminatus-KKN-VL34
acuminatus-KKL-LVL5
acuminatus-KKL-LVL9
acuminatus-KKL-VL24
acuminatus-KKL-LVL7
acuminatus-KKL-LVL8
acuminatus-KKL-VL49
acuminatus-KKL-VL50
acuminatus-KKH-VL26
acuminatus-KKH-VL27
acuminatus-KKH-VL28
acuminatus-KKH-VL33
acuminatus-KKH-LVL10
acuminatus-KKH-VL25
superbus-BLS-CY1
superbus-BLS-CY2
bracteatus-BSY-CY67
bracteatus-BSY-CY70
bracteatus-BSY-CY68
bracteatus-BSY-CY69
bracteatus-LD-CY57
bracteatus-LD-CY60
bracteatus-SJS-CN23
bracteatus-MS-CY3
bracteatus-DWS-CY22
bracteatus-DWS-CY26
bracteatus-DWS-CY30
bracteatus-ADK-CY85
bracteatus-BJP-CY17
bracteatus-BJP-CY18
bracteatus-GLG-CY101
bracteatus-GLG-CY99
bracteatus-BHL-CY90
wardii-BHL-CY120
wardii-BHL-CY91
jouyi-XTBG-CY86
hookeri-BT-CY94
0.06

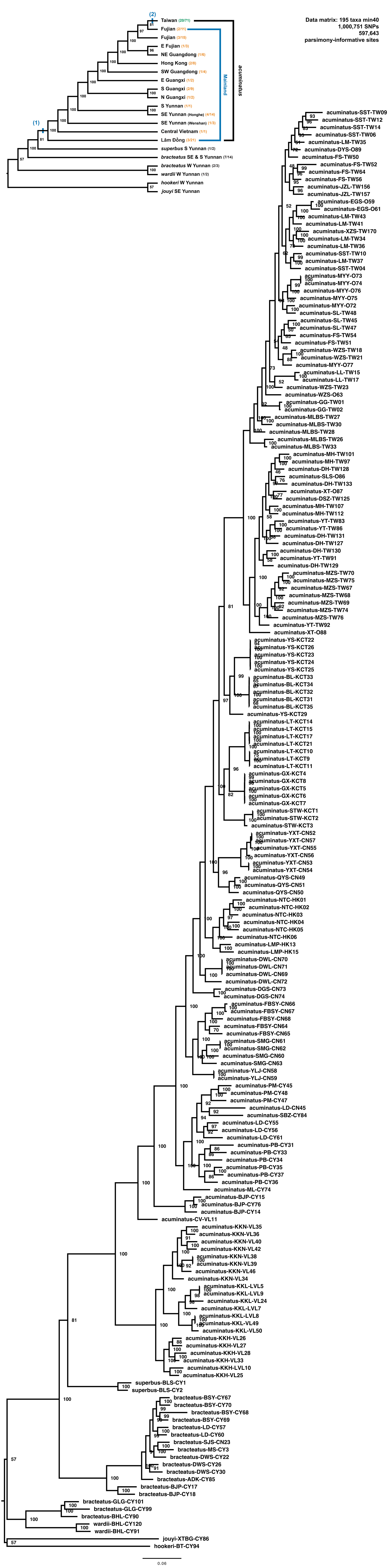

Data matrix: 195 taxa min40
1,000,751 SNPs
597,643
parsimony-informative sites
(1)
(2)
Taiwan (20/71)
Fujian (2/11)
Fujian (3/15)
E Fujian (1/3)
NE Guangdong (1/6)
Hong Kong (2/8)
SW Guangdong (1/4)
E Guangxi (1/2)
S Guangxi (2/9)
N Guangxi (1/2)
S Yunnan (1/1)
SE Yunnan (Honghe) (4/14)
SE Yunnan (Wenshan) (1/3)
Central Vietnam (1/1)
Lâm Đồng (3/21)
superbus S Yunnan (1/2)
bracteatus SE & S Yunnan (7/14)
bracteatus W Yunnan (2/3)
wardii W Yunnan (1/2)
hookeri W Yunnan
jouyi SE Yunnan
Mainland
acuminatus
acuminatus-SST-TW09
acuminatus-SST-TW12
acuminatus-SST-TW14
acuminatus-SST-TW06
acuminatus-LM-TW35
acuminatus-DYS-O89
acuminatus-FS-TW50
acuminatus-FS-TW52
acuminatus-FS-TW64
acuminatus-FS-TW56
acuminatus-JZL-TW156
acuminatus-JZL-TW157
acuminatus-EGS-O59
acuminatus-EGS-O61
acuminatus-LM-TW43
acuminatus-LM-TW41
acuminatus-XZS-TW170
acuminatus-LM-TW34
acuminatus-LM-TW36
acuminatus-SST-TW10
acuminatus-LM-TW37
acuminatus-SST-TW04
acuminatus-MYY-O73
acuminatus-MYY-O74
acuminatus-MYY-O76
acuminatus-MYY-O75
acuminatus-SL-TW48
acuminatus-SL-TW45
acuminatus-SL-TW47
acuminatus-FS-TW54
acuminatus-FS-TW51
acuminatus-WZS-TW18
acuminatus-WZS-TW21
acuminatus-MYY-O77
acuminatus-LL-TW15
acuminatus-LL-TW17
acuminatus-WZS-TW23
acuminatus-WZS-O63
acuminatus-GG-TW01
acuminatus-GG-TW02
acuminatus-MLBS-TW27
acuminatus-MLBS-TW30
acuminatus-MLBS-TW28
acuminatus-MLBS-TW26
acuminatus-MLBS-TW33
acuminatus-MH-TW101
acuminatus-MH-TW97
acuminatus-DH-TW128
acuminatus-SLS-O86
acuminatus-DH-TW133
acuminatus-XT-O87
acuminatus-DSZ-TW125
acuminatus-MH-TW107
acuminatus-MH-TW112
acuminatus-YT-TW83
acuminatus-YT-TW86
acuminatus-DH-TW131
acuminatus-DH-TW127
acuminatus-DH-TW130
acuminatus-YT-TW91
acuminatus-DH-TW129
acuminatus-MZS-TW70
acuminatus-MZS-TW75
acuminatus-MZS-TW67
acuminatus-MZS-TW68
acuminatus-MZS-TW69
acuminatus-MZS-TW74
acuminatus-MZS-TW76
acuminatus-YT-TW92
acuminatus-XT-O88
acuminatus-YS-KCT22
acuminatus-YS-KCT26
acuminatus-YS-KCT23
acuminatus-YS-KCT24
acuminatus-YS-KCT25
acuminatus-BL-KCT33
acuminatus-BL-KCT34
acuminatus-BL-KCT32
acuminatus-BL-KCT31
acuminatus-BL-KCT35
acuminatus-YS-KCT29
acuminatus-LT-KCT14
acuminatus-LT-KCT15
acuminatus-LT-KCT17
acuminatus-LT-KCT21
acuminatus-LT-KCT10
acuminatus-LT-KCT9
acuminatus-LT-KCT11
acuminatus-GX-KCT4
acuminatus-GX-KCT8
acuminatus-GX-KCT5
acuminatus-GX-KCT6
acuminatus-GX-KCT7
acuminatus-STW-KCT1
acuminatus-STW-KCT2
acuminatus-STW-KCT3
acuminatus-YXT-CN52
acuminatus-YXT-CN57
acuminatus-YXT-CN55
acuminatus-YXT-CN56
acuminatus-YXT-CN53
acuminatus-YXT-CN54
acuminatus-QYS-CN49
acuminatus-QYS-CN51
acuminatus-QYS-CN50
acuminatus-NTC-HK01
acuminatus-NTC-HK02
acuminatus-NTC-HK03
acuminatus-NTC-HK04
acuminatus-NTC-HK05
acuminatus-NTC-HK06
acuminatus-LMP-HK13
acuminatus-LMP-HK15
acuminatus-DWL-CN70
acuminatus-DWL-CN71
acuminatus-DWL-CN69
acuminatus-DWL-CN72
acuminatus-DGS-CN73
acuminatus-DGS-CN74
acuminatus-FBSY-CN66
acuminatus-FBSY-CN67
acuminatus-FBSY-CN68
acuminatus-FBSY-CN64
acuminatus-FBSY-CN65
acuminatus-SMG-CN61
acuminatus-SMG-CN62
acuminatus-SMG-CN60
acuminatus-SMG-CN63
acuminatus-YLJ-CN58
acuminatus-YLJ-CN59
acuminatus-PM-CY45
acuminatus-PM-CY48
acuminatus-PM-CY47
acuminatus-LD-CN45
acuminatus-SBZ-CY84
acuminatus-LD-CY55
acuminatus-LD-CY56
acuminatus-LD-CY61
acuminatus-PB-CY31
acuminatus-PB-CY33
acuminatus-PB-CY34
acuminatus-PB-CY35
acuminatus-PB-CY37
acuminatus-PB-CY36
acuminatus-ML-CY74
acuminatus-BJP-CY15
acuminatus-BJP-CY76
acuminatus-BJP-CY14
acuminatus-CV-VL11
acuminatus-KKN-VL35
acuminatus-KKN-VL36
acuminatus-KKN-VL40
acuminatus-KKN-VL42
acuminatus-KKN-VL38
acuminatus-KKN-VL39
acuminatus-KKN-VL46
acuminatus-KKN-VL34
acuminatus-KKL-LVL5
acuminatus-KKL-LVL9
acuminatus-KKL-VL24
acuminatus-KKL-LVL7
acuminatus-KKL-LVL8
acuminatus-KKL-VL49
acuminatus-KKL-VL50
acuminatus-KKH-VL26
acuminatus-KKH-VL27
acuminatus-KKH-VL28
acuminatus-KKH-VL33
acuminatus-KKH-LVL10
acuminatus-KKH-VL25
superbus-BLS-CY1
superbus-BLS-CY2
bracteatus-BSY-CY67
bracteatus-BSY-CY70
bracteatus-BSY-CY68
bracteatus-BSY-CY69
bracteatus-LD-CY57
bracteatus-LD-CY60
bracteatus-SJS-CN23
bracteatus-MS-CY3
bracteatus-DWS-CY22
bracteatus-DWS-CY26
bracteatus-DWS-CY30
bracteatus-ADK-CY85
bracteatus-BJP-CY17
bracteatus-BJP-CY18
bracteatus-GLG-CY101
bracteatus-GLG-CY99
bracteatus-BHL-CY90
wardii-BHL-CY120
wardii-BHL-CY91
jouyi-XTBG-CY86
hookeri-BT-CY94
0.06

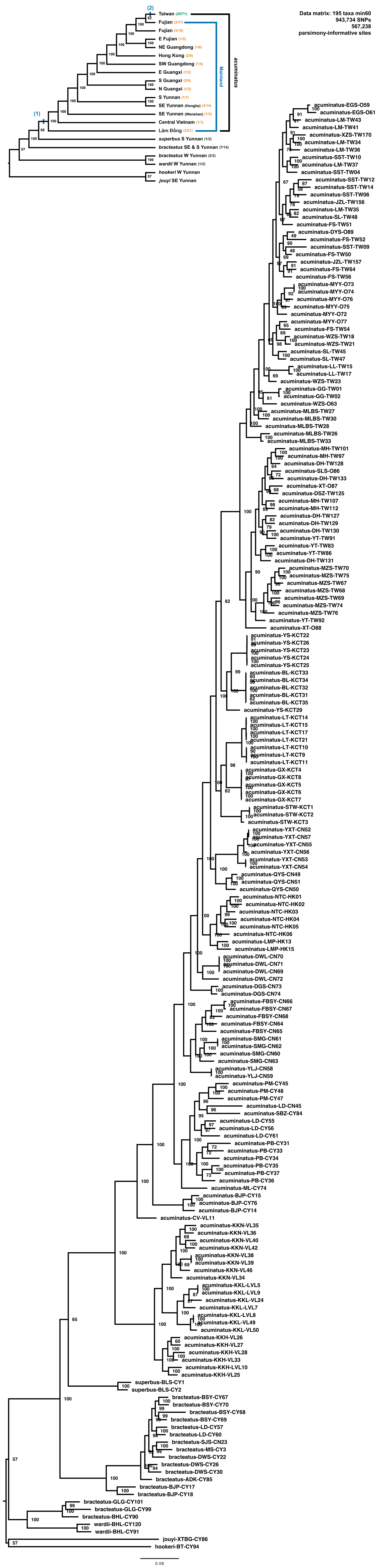

(2)
Taiwan (20/71)
Fujian (2/11)
Fujian (3/15)
E Fujian (1/3)
NE Guangdong (1/6)
Hong Kong (2/8)
SW Guangdong (1/4)
E Guangxi (1/2)
S Guangxi (2/9)
N Guangxi (1/2)
S Yunnan (1/1)
SE Yunnan (Honghe) (4/14)
SE Yunnan (Wenshan) (1/3)
Central Vietnam (1/1)
Lâm Đồng (3/21)
(1)
superbus S Yunnan (1/2)
bracteatus SE & S Yunnan (7/14)
bracteatus W Yunnan (2/3)
wardii W Yunnan (1/2)
hookeri W Yunnan
jouyi SE Yunnan
Mainland
acuminatus
Data matrix: 195 taxa min60
943,734 SNPs
567,238
parsimony-informative sites
acuminatus-EGS-O59
acuminatus-EGS-O61
acuminatus-LM-TW43
acuminatus-LM-TW41
acuminatus-XZS-TW170
acuminatus-LM-TW34
acuminatus-LM-TW36
acuminatus-SST-TW10
acuminatus-LM-TW37
acuminatus-SST-TW04
acuminatus-SST-TW12
acuminatus-SST-TW14
acuminatus-SST-TW06
acuminatus-JZL-TW156
acuminatus-LM-TW35
acuminatus-SL-TW48
acuminatus-FS-TW51
acuminatus-DYS-O89
acuminatus-FS-TW52
acuminatus-SST-TW09
acuminatus-FS-TW50
acuminatus-JZL-TW157
acuminatus-FS-TW64
acuminatus-FS-TW56
acuminatus-MYY-O73
acuminatus-MYY-O74
acuminatus-MYY-O76
acuminatus-MYY-O75
acuminatus-MYY-O72
acuminatus-MYY-O77
acuminatus-FS-TW54
acuminatus-WZS-TW18
acuminatus-WZS-TW21
acuminatus-SL-TW45
acuminatus-SL-TW47
acuminatus-LL-TW15
acuminatus-LL-TW17
acuminatus-WZS-TW23
acuminatus-GG-TW01
acuminatus-GG-TW02
acuminatus-WZS-O63
acuminatus-MLBS-TW27
acuminatus-MLBS-TW30
acuminatus-MLBS-TW28
acuminatus-MLBS-TW26
acuminatus-MLBS-TW33
acuminatus-MH-TW101
acuminatus-MH-TW97
acuminatus-DH-TW128
acuminatus-SLS-O86
acuminatus-DH-TW133
acuminatus-XT-O87
acuminatus-DSZ-TW125
acuminatus-MH-TW107
acuminatus-MH-TW112
acuminatus-DH-TW127
acuminatus-DH-TW129
acuminatus-DH-TW130
acuminatus-YT-TW91
acuminatus-YT-TW83
acuminatus-YT-TW86
acuminatus-DH-TW131
acuminatus-MZS-TW70
acuminatus-MZS-TW75
acuminatus-MZS-TW67
acuminatus-MZS-TW68
acuminatus-MZS-TW69
acuminatus-MZS-TW74
acuminatus-MZS-TW76
acuminatus-YT-TW92
acuminatus-XT-O88
acuminatus-YS-KCT22
acuminatus-YS-KCT26
acuminatus-YS-KCT23
acuminatus-YS-KCT24
acuminatus-YS-KCT25
acuminatus-BL-KCT33
acuminatus-BL-KCT34
acuminatus-BL-KCT32
acuminatus-BL-KCT31
acuminatus-BL-KCT35
acuminatus-YS-KCT29
acuminatus-LT-KCT14
acuminatus-LT-KCT15
acuminatus-LT-KCT17
acuminatus-LT-KCT21
acuminatus-LT-KCT10
acuminatus-LT-KCT9
acuminatus-LT-KCT11
acuminatus-GX-KCT4
acuminatus-GX-KCT8
acuminatus-GX-KCT5
acuminatus-GX-KCT6
acuminatus-GX-KCT7
acuminatus-STW-KCT1
acuminatus-STW-KCT2
acuminatus-STW-KCT3
acuminatus-YXT-CN52
acuminatus-YXT-CN57
acuminatus-YXT-CN55
acuminatus-YXT-CN56
acuminatus-YXT-CN53
acuminatus-YXT-CN54
acuminatus-QYS-CN49
acuminatus-QYS-CN51
acuminatus-QYS-CN50
acuminatus-NTC-HK01
acuminatus-NTC-HK02
acuminatus-NTC-HK03
acuminatus-NTC-HK04
acuminatus-NTC-HK05
acuminatus-NTC-HK06
acuminatus-LMP-HK13
acuminatus-LMP-HK15
acuminatus-DWL-CN70
acuminatus-DWL-CN71
acuminatus-DWL-CN69
acuminatus-DWL-CN72
acuminatus-DGS-CN73
acuminatus-DGS-CN74
acuminatus-FBSY-CN66
acuminatus-FBSY-CN67
acuminatus-FBSY-CN68
acuminatus-FBSY-CN64
acuminatus-FBSY-CN65
acuminatus-SMG-CN61
acuminatus-SMG-CN62
acuminatus-SMG-CN60
acuminatus-SMG-CN63
acuminatus-YLJ-CN58
acuminatus-YLJ-CN59
acuminatus-PM-CY45
acuminatus-PM-CY48
acuminatus-PM-CY47
acuminatus-LD-CN45
acuminatus-SBZ-CY84
acuminatus-LD-CY55
acuminatus-LD-CY56
acuminatus-LD-CY61
acuminatus-PB-CY31
acuminatus-PB-CY33
acuminatus-PB-CY34
acuminatus-PB-CY35
acuminatus-PB-CY37
acuminatus-PB-CY36
acuminatus-ML-CY74
acuminatus-BJP-CY15
acuminatus-BJP-CY76
acuminatus-BJP-CY14
acuminatus-CV-VL11
acuminatus-KKN-VL35
acuminatus-KKN-VL36
acuminatus-KKN-VL40
acuminatus-KKN-VL42
acuminatus-KKN-VL38
acuminatus-KKN-VL39
acuminatus-KKN-VL46
acuminatus-KKN-VL34
acuminatus-KKL-LVL5
acuminatus-KKL-VL9
acuminatus-KKL-VL24
acuminatus-KKL-LVL7
acuminatus-KKL-LVL8
acuminatus-KKL-VL49
acuminatus-KKL-VL50
acuminatus-KKH-VL26
acuminatus-KKH-VL27
acuminatus-KKH-VL28
acuminatus-KKH-VL33
acuminatus-KKH-LVL10
acuminatus-KKH-VL25
superbus-BLS-CY1
superbus-BLS-CY2
bracteatus-BSY-CY67
bracteatus-BSY-CY70
bracteatus-BSY-CY68
bracteatus-BSY-CY69
bracteatus-LD-CY57
bracteatus-LD-CY60
bracteatus-SJS-CN23
bracteatus-MS-CY3
bracteatus-DWS-CY22
bracteatus-DWS-CY26
bracteatus-DWS-CY30
bracteatus-ADK-CY85
bracteatus-BJP-CY17
bracteatus-BJP-CY18
bracteatus-GLG-CY101
bracteatus-GLG-CY99
bracteatus-BHL-CY90
wardii-BHL-CY120
wardii-BHL-CY91
jouyi-XTBG-CY86
hookeri-BT-CY94
0.06

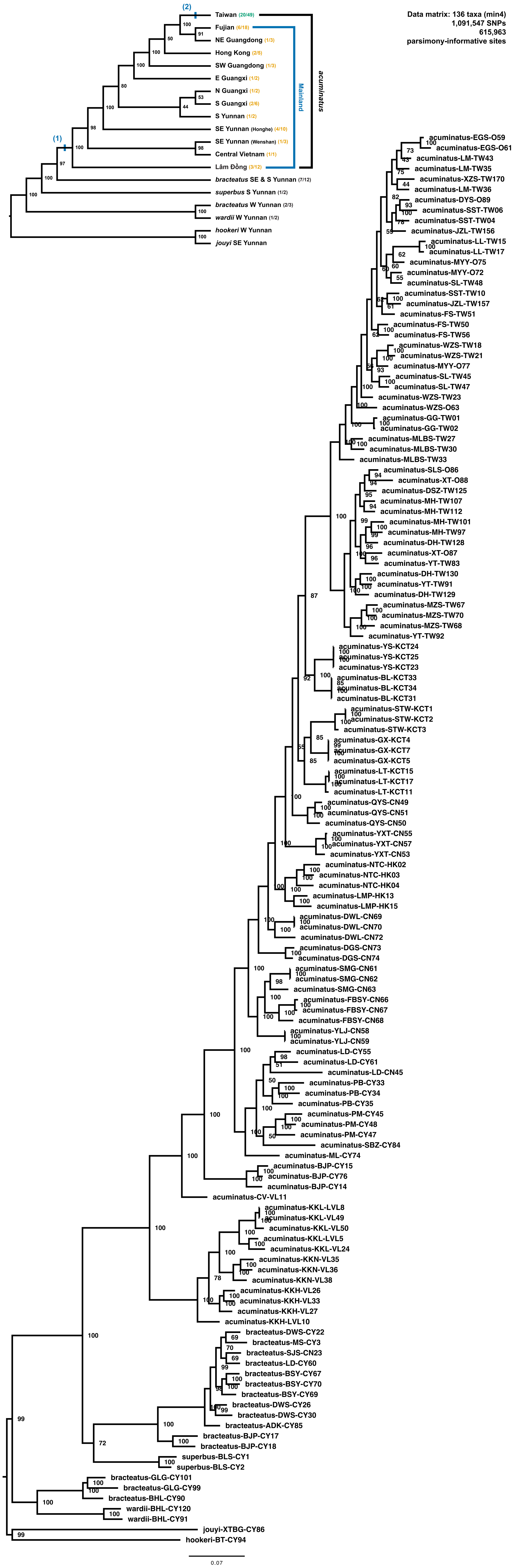

Data matrix: 136 taxa (min4)
1,091,547 SNPs
615,963
parsimony-informative sites
(1)
(2)
Taiwan (20/49)
Fujian (6/18)
NE Guangdong (1/3)
Hong Kong (2/5)
SW Guangdong (1/3)
E Guangxi (1/2)
N Guangxi (1/2)
S Guangxi (2/6)
S Yunnan (1/2)
SE Yunnan (Honghe) (4/10)
SE Yunnan (Wenshan) (1/3)
Central Vietnam (1/1)
Lâm Đồng (3/12)
bracteatus SE & S Yunnan (7/12)
superbus S Yunnan (1/2)
bracteatus W Yunnan (2/3)
wardii W Yunnan (1/2)
hookeri W Yunnan
jouyi SE Yunnan
Mainland
acuminatus
acuminatus-EGS-O59
acuminatus-EGS-O61
acuminatus-LM-TW43
acuminatus-LM-TW35
acuminatus-XZS-TW170
acuminatus-LM-TW36
acuminatus-DYS-O89
acuminatus-SST-TW06
acuminatus-SST-TW04
acuminatus-JZL-TW156
acuminatus-LL-TW15
acuminatus-LL-TW17
acuminatus-MYY-O75
acuminatus-MYY-O72
acuminatus-SL-TW48
acuminatus-SST-TW10
acuminatus-JZL-TW157
acuminatus-FS-TW51
acuminatus-FS-TW50
acuminatus-FS-TW56
acuminatus-WZS-TW18
acuminatus-WZS-TW21
acuminatus-MYY-O77
acuminatus-SL-TW45
acuminatus-SL-TW47
acuminatus-WZS-TW23
acuminatus-WZS-O63
acuminatus-GG-TW01
acuminatus-GG-TW02
acuminatus-MLBS-TW27
acuminatus-MLBS-TW30
acuminatus-MLBS-TW33
acuminatus-SLS-O86
acuminatus-XT-O88
acuminatus-DSZ-TW125
acuminatus-MH-TW107
acuminatus-MH-TW112
acuminatus-MH-TW101
acuminatus-MH-TW97
acuminatus-DH-TW128
acuminatus-XT-O87
acuminatus-YT-TW83
acuminatus-DH-TW130
acuminatus-YT-TW91
acuminatus-DH-TW129
acuminatus-MZS-TW67
acuminatus-MZS-TW70
acuminatus-MZS-TW68
acuminatus-YT-TW92
acuminatus-YS-KCT24
acuminatus-YS-KCT25
acuminatus-YS-KCT23
acuminatus-BL-KCT33
acuminatus-BL-KCT34
acuminatus-BL-KCT31
acuminatus-STW-KCT1
acuminatus-STW-KCT2
acuminatus-STW-KCT3
acuminatus-GX-KCT4
acuminatus-GX-KCT7
acuminatus-GX-KCT5
acuminatus-LT-KCT15
acuminatus-LT-KCT17
acuminatus-LT-KCT11
acuminatus-QYS-CN49
acuminatus-QYS-CN51
acuminatus-QYS-CN50
acuminatus-YXT-CN55
acuminatus-YXT-CN57
acuminatus-YXT-CN53
acuminatus-NTC-HK02
acuminatus-NTC-HK03
acuminatus-NTC-HK04
acuminatus-LMP-HK13
acuminatus-LMP-HK15
acuminatus-DWL-CN69
acuminatus-DWL-CN70
acuminatus-DWL-CN72
acuminatus-DGS-CN73
acuminatus-DGS-CN74
acuminatus-SMG-CN61
acuminatus-SMG-CN62
acuminatus-SMG-CN63
acuminatus-FBSY-CN66
acuminatus-FBSY-CN67
acuminatus-FBSY-CN68
acuminatus-YLJ-CN58
acuminatus-YLJ-CN59
acuminatus-LD-CY55
acuminatus-LD-CY61
acuminatus-LD-CN45
acuminatus-PB-CY33
acuminatus-PB-CY34
acuminatus-PB-CY35
acuminatus-PM-CY45
acuminatus-PM-CY48
acuminatus-PM-CY47
acuminatus-SBZ-CY84
acuminatus-ML-CY74
acuminatus-BJP-CY15
acuminatus-BJP-CY76
acuminatus-BJP-CY14
acuminatus-CV-VL11
acuminatus-KKL-LVL8
acuminatus-KKL-VL49
acuminatus-KKL-VL50
acuminatus-KKL-LVL5
acuminatus-KKL-VL24
acuminatus-KKN-VL35
acuminatus-KKN-VL36
acuminatus-KKN-VL38
acuminatus-KKH-VL26
acuminatus-KKH-VL33
acuminatus-KKH-VL27
acuminatus-KKH-LVL10
bracteatus-DWS-CY22
bracteatus-MS-CY3
bracteatus-SJS-CN23
bracteatus-LD-CY60
bracteatus-BSY-CY67
bracteatus-BSY-CY70
bracteatus-BSY-CY69
bracteatus-DWS-CY26
bracteatus-DWS-CY30
bracteatus-ADK-CY85
bracteatus-BJP-CY17
bracteatus-BJP-CY18
superbus-BLS-CY1
superbus-BLS-CY2
bracteatus-GLG-CY101
bracteatus-GLG-CY99
bracteatus-BHL-CY90
wardii-BHL-CY120
wardii-BHL-CY91
jouyi-XTBG-CY86
hookeri-BT-CY94
0.07

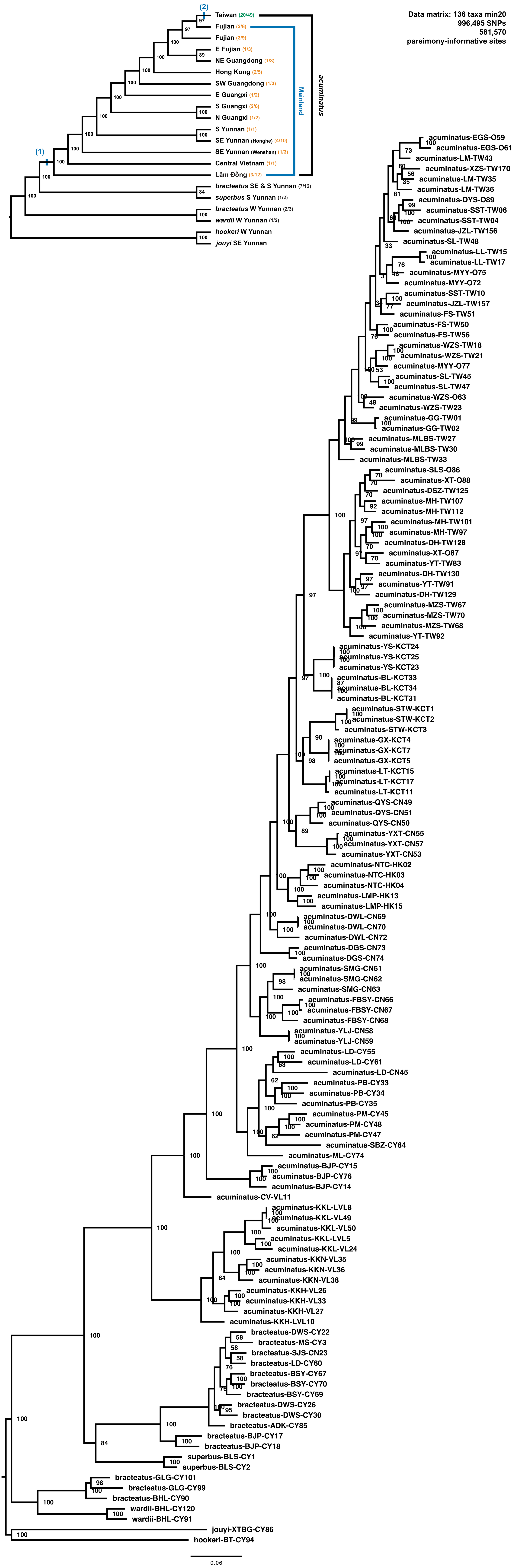

Data matrix: 136 taxa min20
996,495 SNPs
581,570
parsimony-informative sites
(1)
(2)
Taiwan (20/49)
Fujian (2/6)
Fujian (3/9)
E Fujian (1/3)
NE Guangdong (1/3)
Hong Kong (2/5)
SW Guangdong (1/3)
E Guangxi (1/2)
S Guangxi (2/6)
N Guangxi (1/2)
S Yunnan (1/1)
SE Yunnan (Honghe) (4/10)
SE Yunnan (Wenshan) (1/3)
Central Vietnam (1/1)
Lâm Đồng (3/12)
bracteatus SE & S Yunnan (7/12)
superbus S Yunnan (1/2)
bracteatus W Yunnan (2/3)
wardii W Yunnan (1/2)
hookeri W Yunnan
jouyi SE Yunnan
Mainland
acuminatus
acuminatus-EGS-O59
acuminatus-EGS-O61
acuminatus-LM-TW43
acuminatus-XZS-TW170
acuminatus-LM-TW35
acuminatus-LM-TW36
acuminatus-DYS-O89
acuminatus-SST-TW06
acuminatus-SST-TW04
acuminatus-JZL-TW156
acuminatus-SL-TW48
acuminatus-LL-TW15
acuminatus-LL-TW17
acuminatus-MYY-O75
acuminatus-MYY-O72
acuminatus-SST-TW10
acuminatus-JZL-TW157
acuminatus-FS-TW51
acuminatus-FS-TW50
acuminatus-FS-TW56
acuminatus-WZS-TW18
acuminatus-WZS-TW21
acuminatus-MYY-O77
acuminatus-SL-TW45
acuminatus-SL-TW47
acuminatus-WZS-O63
acuminatus-WZS-TW23
acuminatus-GG-TW01
acuminatus-GG-TW02
acuminatus-MLBS-TW27
acuminatus-MLBS-TW30
acuminatus-MLBS-TW33
acuminatus-SLS-O86
acuminatus-XT-O88
acuminatus-DSZ-TW125
acuminatus-MH-TW107
acuminatus-MH-TW112
acuminatus-MH-TW101
acuminatus-MH-TW97
acuminatus-DH-TW128
acuminatus-XT-O87
acuminatus-YT-TW83
acuminatus-DH-TW130
acuminatus-YT-TW91
acuminatus-DH-TW129
acuminatus-MZS-TW67
acuminatus-MZS-TW70
acuminatus-MZS-TW68
acuminatus-YT-TW92
acuminatus-YS-KCT24
acuminatus-YS-KCT25
acuminatus-YS-KCT23
acuminatus-BL-KCT33
acuminatus-BL-KCT34
acuminatus-BL-KCT31
acuminatus-STW-KCT1
acuminatus-STW-KCT2
acuminatus-STW-KCT3
acuminatus-GX-KCT4
acuminatus-GX-KCT7
acuminatus-GX-KCT5
acuminatus-LT-KCT15
acuminatus-LT-KCT17
acuminatus-LT-KCT11
acuminatus-QYS-CN49
acuminatus-QYS-CN51
acuminatus-QYS-CN50
acuminatus-YXT-CN55
acuminatus-YXT-CN57
acuminatus-YXT-CN53
acuminatus-NTC-HK02
acuminatus-NTC-HK03
acuminatus-NTC-HK04
acuminatus-LMP-HK13
acuminatus-LMP-HK15
acuminatus-DWL-CN69
acuminatus-DWL-CN70
acuminatus-DWL-CN72
acuminatus-DGS-CN73
acuminatus-DGS-CN74
acuminatus-SMG-CN61
acuminatus-SMG-CN62
acuminatus-SMG-CN63
acuminatus-FBSY-CN66
acuminatus-FBSY-CN67
acuminatus-FBSY-CN68
acuminatus-YLJ-CN58
acuminatus-YLJ-CN59
acuminatus-LD-CY55
acuminatus-LD-CY61
acuminatus-LD-CN45
acuminatus-PB-CY33
acuminatus-PB-CY34
acuminatus-PB-CY35
acuminatus-PM-CY45
acuminatus-PM-CY48
acuminatus-PM-CY47
acuminatus-SBZ-CY84
acuminatus-ML-CY74
acuminatus-BJP-CY15
acuminatus-BJP-CY76
acuminatus-BJP-CY14
acuminatus-CV-VL11
acuminatus-KKL-LVL8
acuminatus-KKL-VL49
acuminatus-KKL-VL50
acuminatus-KKL-LVL5
acuminatus-KKL-VL24
acuminatus-KKN-VL35
acuminatus-KKN-VL36
acuminatus-KKN-VL38
acuminatus-KKH-VL26
acuminatus-KKH-VL33
acuminatus-KKH-VL27
acuminatus-KKH-LVL10
bracteatus-DWS-CY22
bracteatus-MS-CY3
bracteatus-SJS-CN23
bracteatus-LD-CY60
bracteatus-BSY-CY67
bracteatus-BSY-CY70
bracteatus-BSY-CY69
bracteatus-DWS-CY26
bracteatus-DWS-CY30
bracteatus-ADK-CY85
bracteatus-BJP-CY17
bracteatus-BJP-CY18
superbus-BLS-CY1
superbus-BLS-CY2
bracteatus-GLG-CY101
bracteatus-GLG-CY99
bracteatus-BHL-CY90
wardii-BHL-CY120
wardii-BHL-CY91
jouyi-XTBG-CY86
hookeri-BT-CY94
0.06

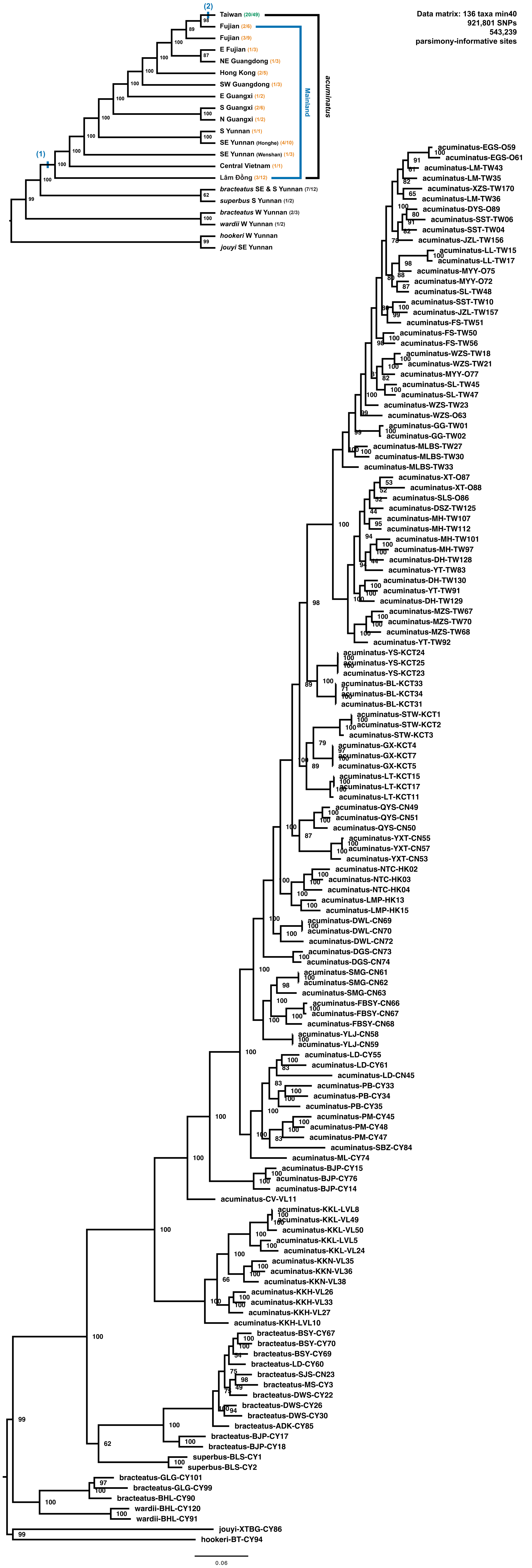

Data matrix: 136 taxa min40
921,801 SNPs
543,239
parsimony-informative sites
(1)
(2)
Taiwan (20/49)
Fujian (2/6)
Fujian (3/9)
E Fujian (1/3)
NE Guangdong (1/3)
Hong Kong (2/5)
SW Guangdong (1/3)
E Guangxi (1/2)
S Guangxi (2/6)
N Guangxi (1/2)
S Yunnan (1/1)
SE Yunnan (Honghe) (4/10)
SE Yunnan (Wenshan) (1/3)
Central Vietnam (1/1)
Lâm Đồng (3/12)
Mainland
acuminatus
bracteatus SE & S Yunnan (7/12)
superbus S Yunnan (1/2)
bracteatus W Yunnan (2/3)
wardii W Yunnan (1/2)
hookeri W Yunnan
jouyi SE Yunnan
acuminatus-EGS-O59
acuminatus-EGS-O61
acuminatus-LM-TW43
acuminatus-LM-TW35
acuminatus-XZS-TW170
acuminatus-LM-TW36
acuminatus-DYS-O89
acuminatus-SST-TW06
acuminatus-SST-TW04
acuminatus-JZL-TW156
acuminatus-LL-TW15
acuminatus-LL-TW17
acuminatus-MYY-O75
acuminatus-MYY-O72
acuminatus-SL-TW48
acuminatus-SST-TW10
acuminatus-JZL-TW157
acuminatus-FS-TW51
acuminatus-FS-TW50
acuminatus-FS-TW56
acuminatus-WZS-TW18
acuminatus-WZS-TW21
acuminatus-MYY-O77
acuminatus-SL-TW45
acuminatus-SL-TW47
acuminatus-WZS-TW23
acuminatus-WZS-O63
acuminatus-GG-TW01
acuminatus-GG-TW02
acuminatus-MLBS-TW27
acuminatus-MLBS-TW30
acuminatus-MLBS-TW33
acuminatus-XT-O87
acuminatus-XT-O88
acuminatus-SLS-O86
acuminatus-DSZ-TW125
acuminatus-MH-TW107
acuminatus-MH-TW112
acuminatus-MH-TW101
acuminatus-MH-TW97
acuminatus-DH-TW128
acuminatus-YT-TW83
acuminatus-DH-TW130
acuminatus-YT-TW91
acuminatus-DH-TW129
acuminatus-MZS-TW67
acuminatus-MZS-TW70
acuminatus-MZS-TW68
acuminatus-YT-TW92
acuminatus-YS-KCT24
acuminatus-YS-KCT25
acuminatus-YS-KCT23
acuminatus-BL-KCT33
acuminatus-BL-KCT34
acuminatus-BL-KCT31
acuminatus-STW-KCT1
acuminatus-STW-KCT2
acuminatus-STW-KCT3
acuminatus-GX-KCT4
acuminatus-GX-KCT7
acuminatus-GX-KCT5
acuminatus-LT-KCT15
acuminatus-LT-KCT17
acuminatus-LT-KCT11
acuminatus-QYS-CN49
acuminatus-QYS-CN51
acuminatus-QYS-CN50
acuminatus-YXT-CN55
acuminatus-YXT-CN57
acuminatus-YXT-CN53
acuminatus-NTC-HK02
acuminatus-NTC-HK03
acuminatus-NTC-HK04
acuminatus-LMP-HK13
acuminatus-LMP-HK15
acuminatus-DWL-CN69
acuminatus-DWL-CN70
acuminatus-DWL-CN72
acuminatus-DGS-CN73
acuminatus-DGS-CN74
acuminatus-SMG-CN61
acuminatus-SMG-CN62
acuminatus-SMG-CN63
acuminatus-FBSY-CN66
acuminatus-FBSY-CN67
acuminatus-FBSY-CN68
acuminatus-YLJ-CN58
acuminatus-YLJ-CN59
acuminatus-LD-CY55
acuminatus-LD-CY61
acuminatus-LD-CN45
acuminatus-PB-CY33
acuminatus-PB-CY34
acuminatus-PB-CY35
acuminatus-PM-CY45
acuminatus-PM-CY48
acuminatus-PM-CY47
acuminatus-SBZ-CY84
acuminatus-ML-CY74
acuminatus-BJP-CY15
acuminatus-BJP-CY76
acuminatus-BJP-CY14
acuminatus-CV-VL11
acuminatus-KKL-LVL8
acuminatus-KKL-VL49
acuminatus-KKL-VL50
acuminatus-KKL-LVL5
acuminatus-KKL-LVL24
acuminatus-KKN-VL35
acuminatus-KKN-VL36
acuminatus-KKN-VL38
acuminatus-KKH-VL26
acuminatus-KKH-VL33
acuminatus-KKH-VL27
acuminatus-KKH-LVL10
bracteatus-BSY-CY67
bracteatus-BSY-CY70
bracteatus-BSY-CY69
bracteatus-LD-CY60
bracteatus-SJS-CN23
bracteatus-MS-CY3
bracteatus-DWS-CY22
bracteatus-DWS-CY26
bracteatus-DWS-CY30
bracteatus-ADK-CY85
bracteatus-BJP-CY17
bracteatus-BJP-CY18
superbus-BLS-CY1
superbus-BLS-CY2
bracteatus-GLG-CY101
bracteatus-GLG-CY99
bracteatus-BHL-CY90
wardii-BHL-CY120
wardii-BHL-CY91
jouyi-XTBG-CY86
hookeri-BT-CY94
0.06

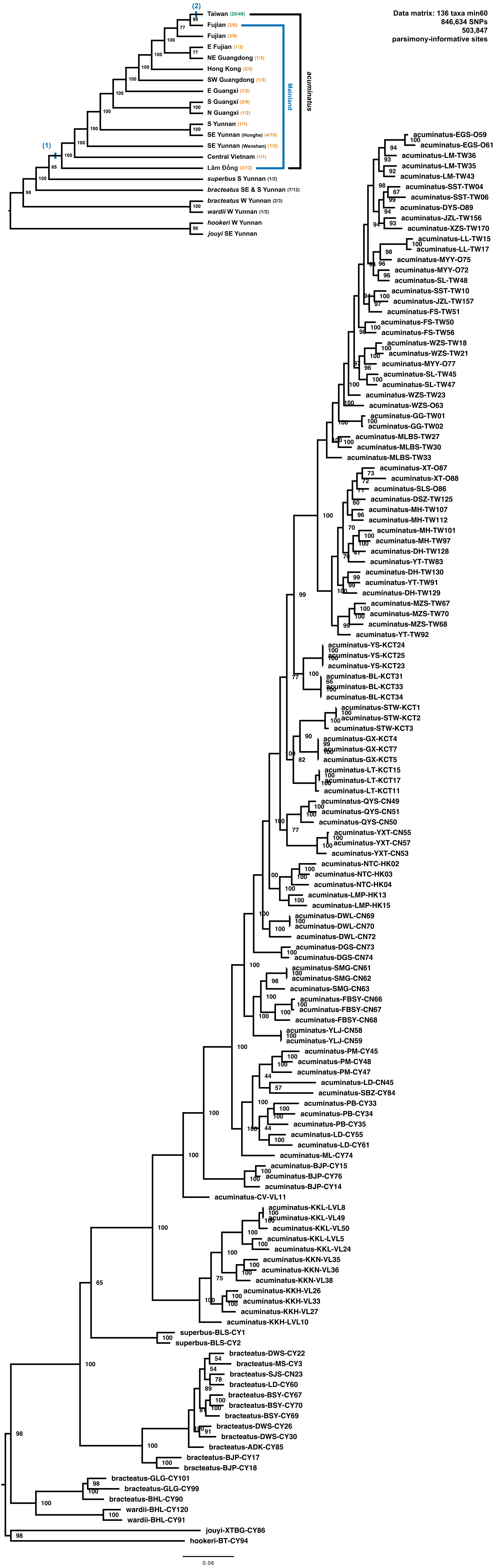
Data matrix: 136 taxa min60
846,634 SNPs
503,847
parsimony-informative sites
acuminatus
Mainland
Taiwan (20/49)
Fujian (2/6)
Fujian (3/9)
E Fujian (1/3)
NE Guangdong (1/3)
Hong Kong (2/5)
SW Guangdong (1/3)
E Guangxi (1/2)
S Guangxi (2/6)
N Guangxi (1/2)
S Yunnan (1/1)
SE Yunnan (Honghe) (4/10)
SE Yunnan (Wenshan) (1/3)
Central Vietnam (1/1)
Lâm Đồng (3/12)
superbus S Yunnan (1/2)
bracteatus SE & S Yunnan (7/12)
bracteatus W Yunnan (2/3)
wardii W Yunnan (1/2)
hookeri W Yunnan
jouyi SE Yunnan
0.06
