# Supplementary material for: Testing macroevolutionary predictions of the Grant‐Stebbins model in the origin of Aeschynanthus acuminatus
Source: New Phytol. 2026 Jan 27;249(6):3137–48. doi: 10.1111/nph.70871 (PMC12917478; doi:10.1111/nph.70871)

New Phytologist Supporting Information

Article title: Testing macroevolutionary predictions of the Grant-Stebbins model in the origin of *Aeschynanthus acuminatus*

Authors: Jing-Yi Lu, Yaowu Xing, Hong Truong Luu, Richard H Ree

Article acceptance date: 4 December 2025

Figure S5. Results of *TreeMix* analyses. The first page shows the log likelihood scores across different admixture edges (m) for (a) the full 195 taxa dataset, (b) the subsample 136 taxa dataset. The following pages are population trees with inferred splits and admixture events for both datasets, shown for m = 1–5. Each page encompasses results from 9 randomly subsampled iterations per dataset and admixture level combination. Abbreviations for populations: WAR = *wardii*; BRAW = *bracteatus* (W Yunnan); BRAW = *bracteatus* (W Yunnan); BRAE = *bracteatus* (SE/S Yunnan); SUP = *superbus*; LD = Lâm Đồng; CV = Central Vietnam; YNSEW = SE Yunnan (Wenshan); YNSEH = SE Yunnan (Honghe); YNS = S Yunnan; GXS = S Guangxi; GXN = N Guangxi; GXE = E Guangxi; GD = SW Guangdong; HK = Hong Kong; FJ = Fujian and NE Guangdong; TW = Taiwan.

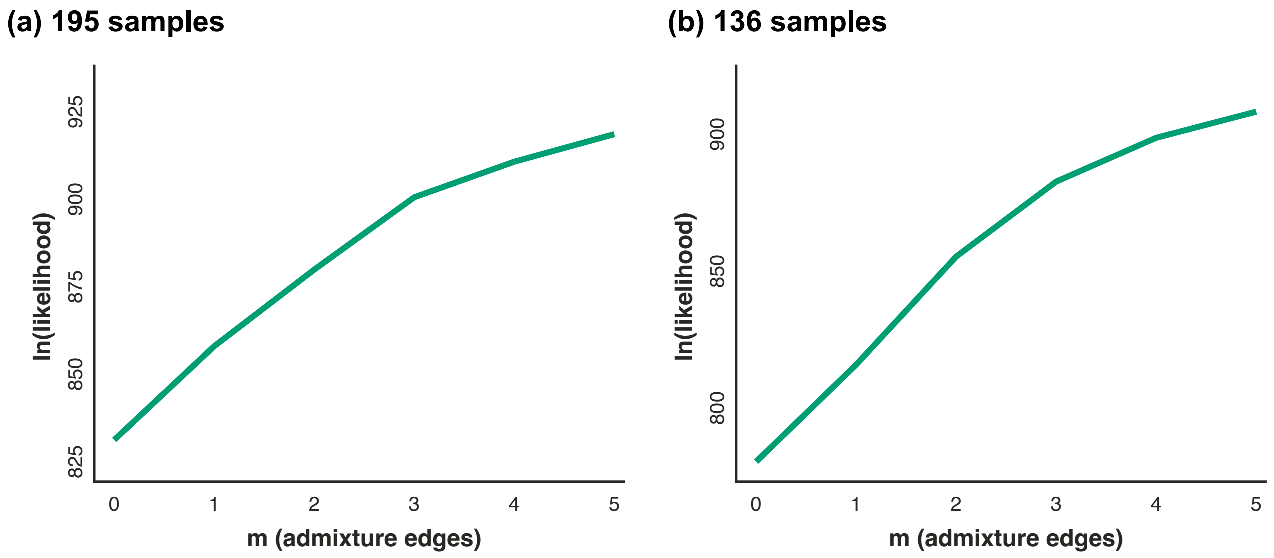
(a) 195 samples
ln(likelihood)
825
850
875
900
925
0
1
2
3
4
5
m (admixture edges)
(b) 136 samples
ln(likelihood)
800
850
900
0
1
2
3
4
5
m (admixture edges)

Data matrix: 195 taxa (min4); m (admixture edges) = 1
10,274 unlink SNPs (loci) sampled from 221,022 SNPs

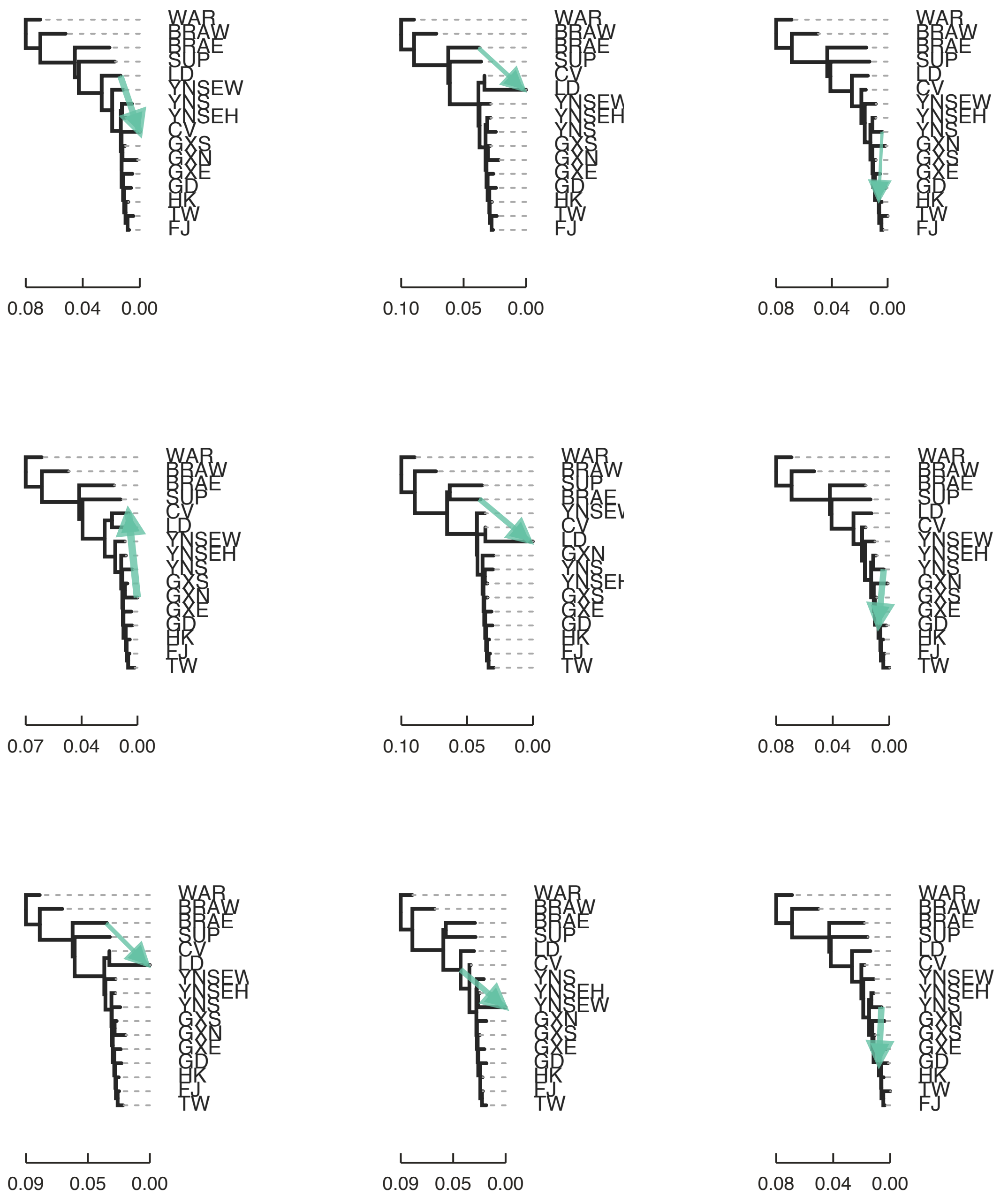

Data matrix: 195 taxa (min4); m (admixture edges) = 2
10,274 unlink SNPs (loci) sampled from 221,022 SNPs

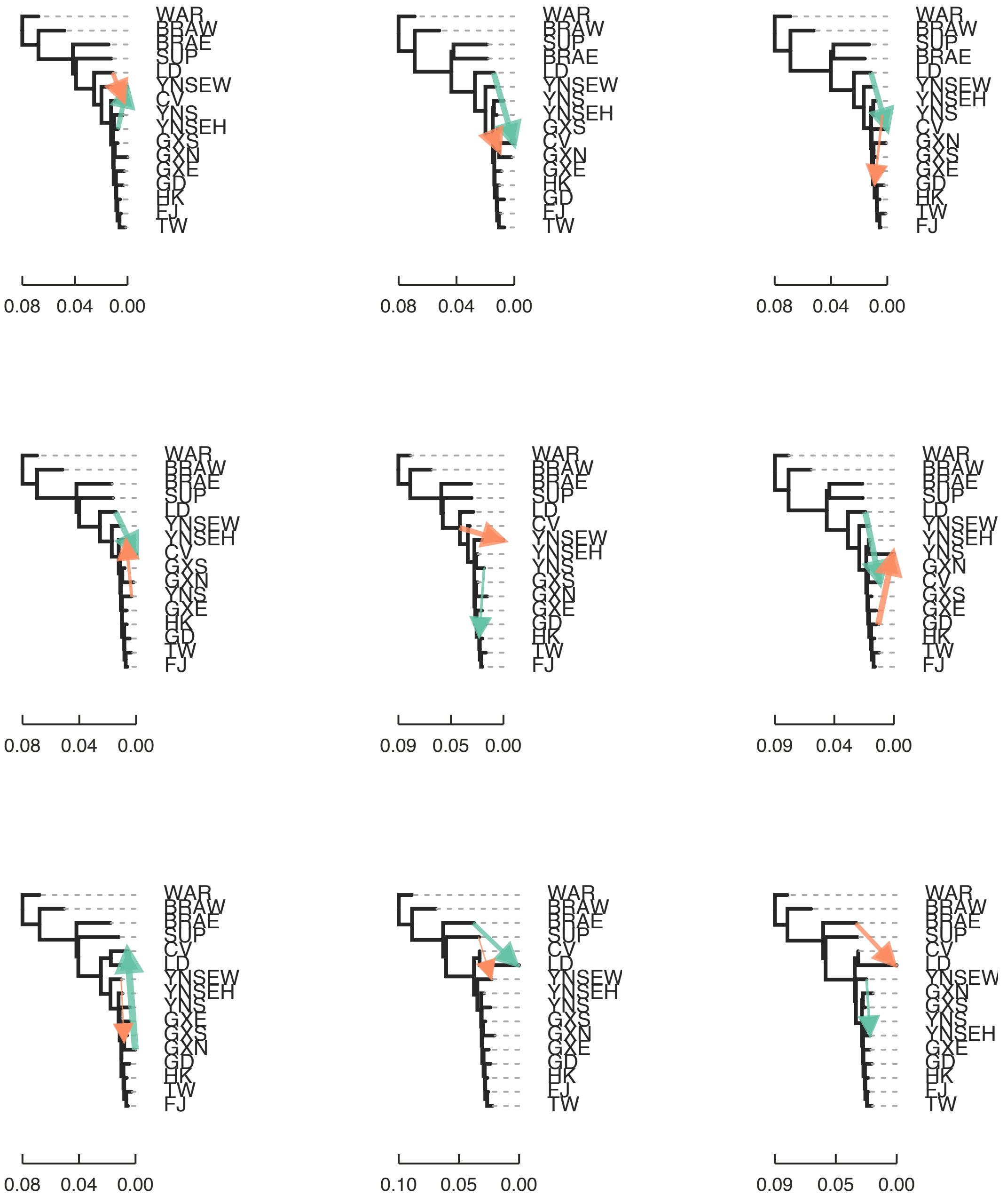

Data matrix: 195 taxa (min4); m (admixture edges) = 3
10,274 unlink SNPs (loci) sampled from 221,022 SNPs

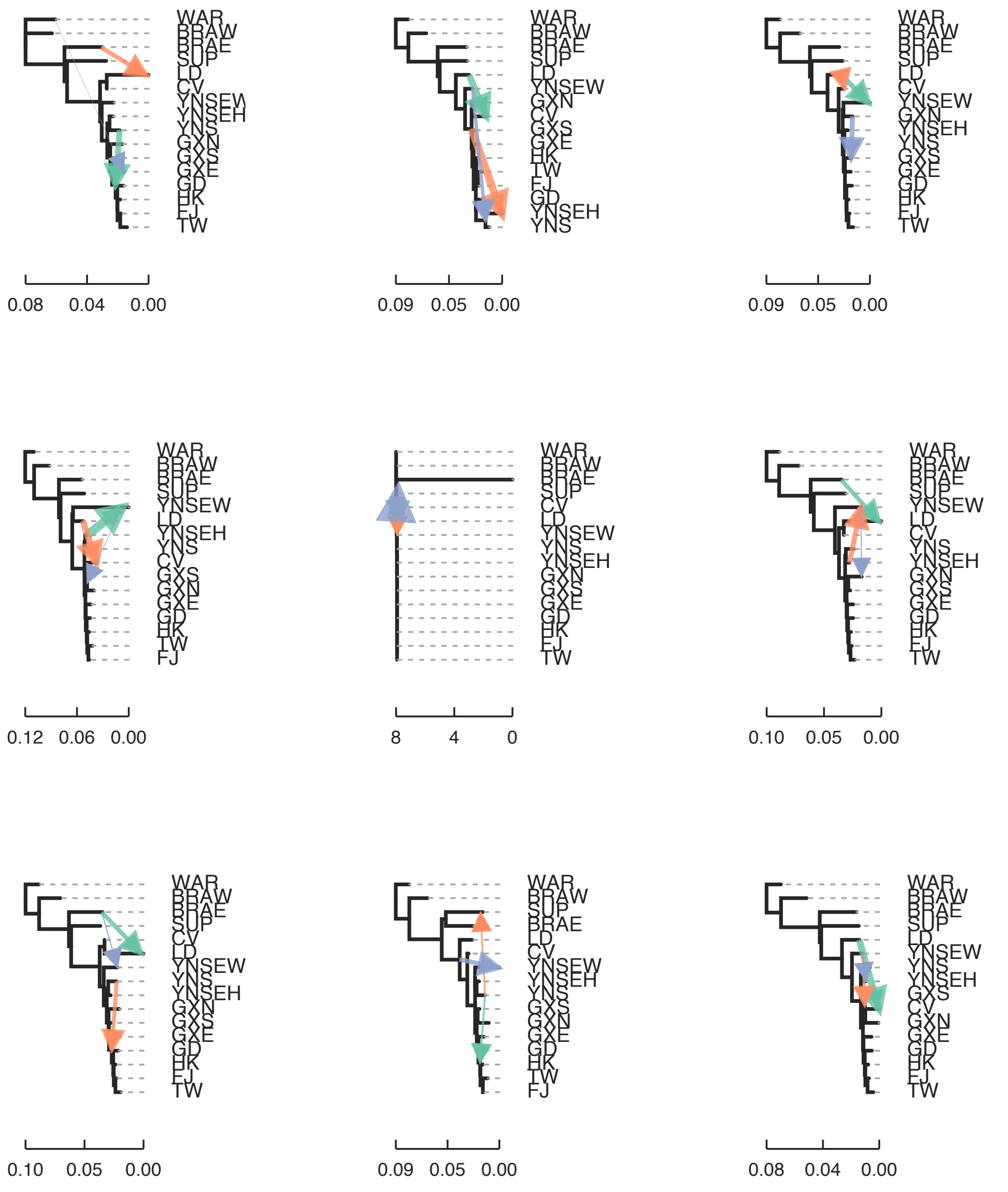

Data matrix: 195 taxa (min4); m (admixture edges) = 4
10,274 unlink SNPs (loci) sampled from 221,022 SNPs

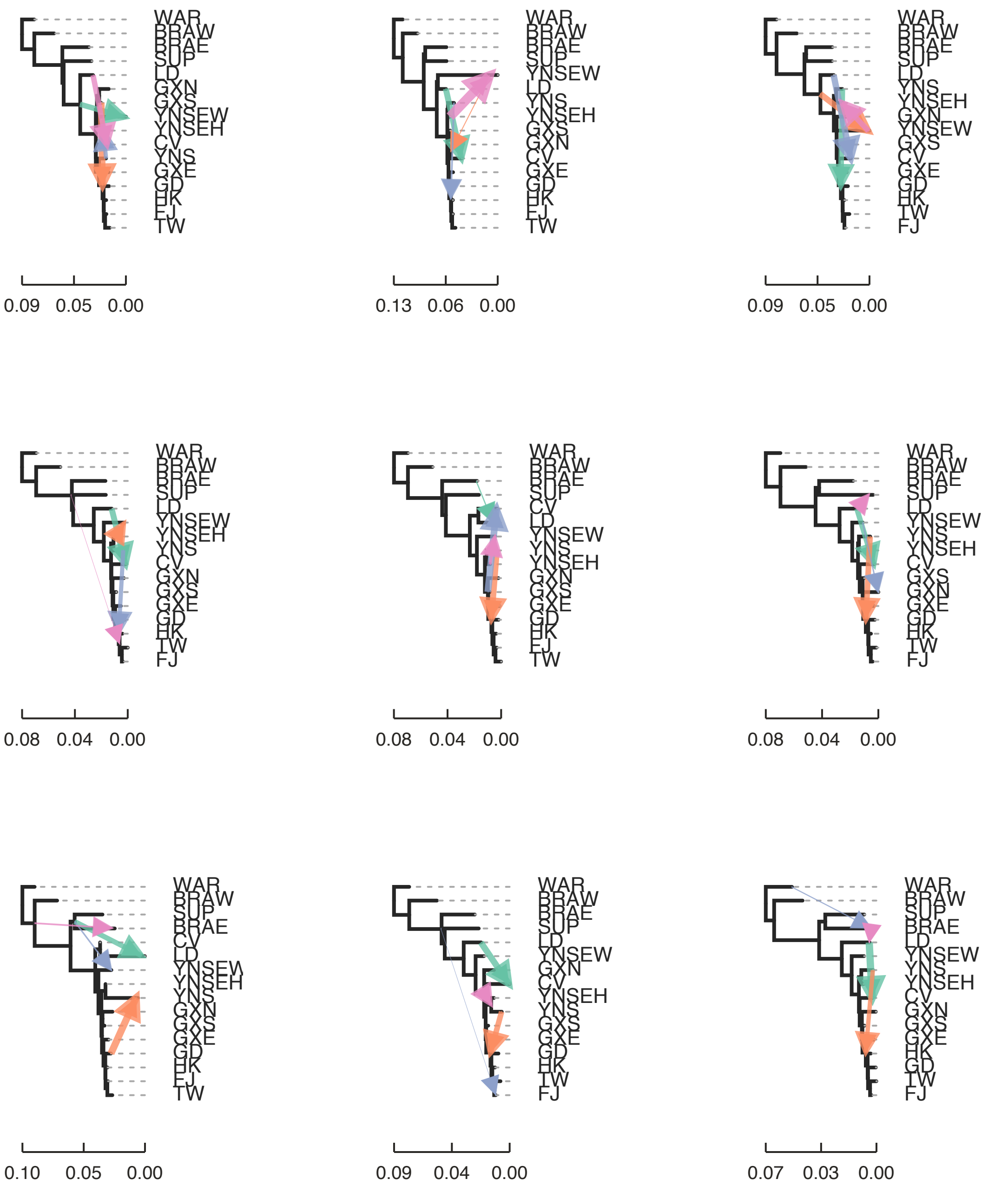

Data matrix: 195 taxa (min4); m (admixture edges) = 5
10,274 unlink SNPs (loci) sampled from 221,022 SNPs

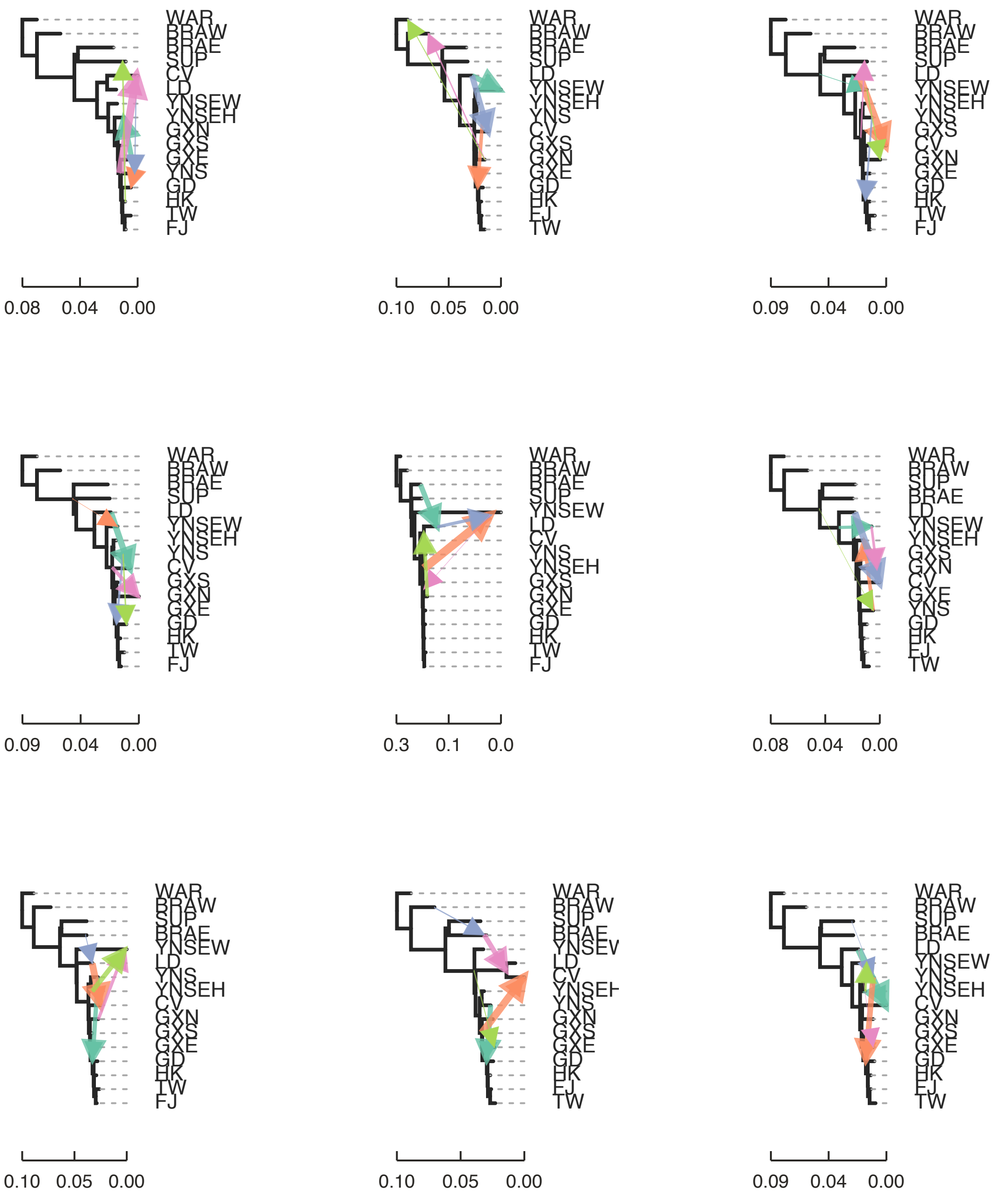

Data matrix: 136 taxa (min4); m (admixture edges) = 1
10,259 unlink SNPs (loci) sampled from 197,779 SNPs

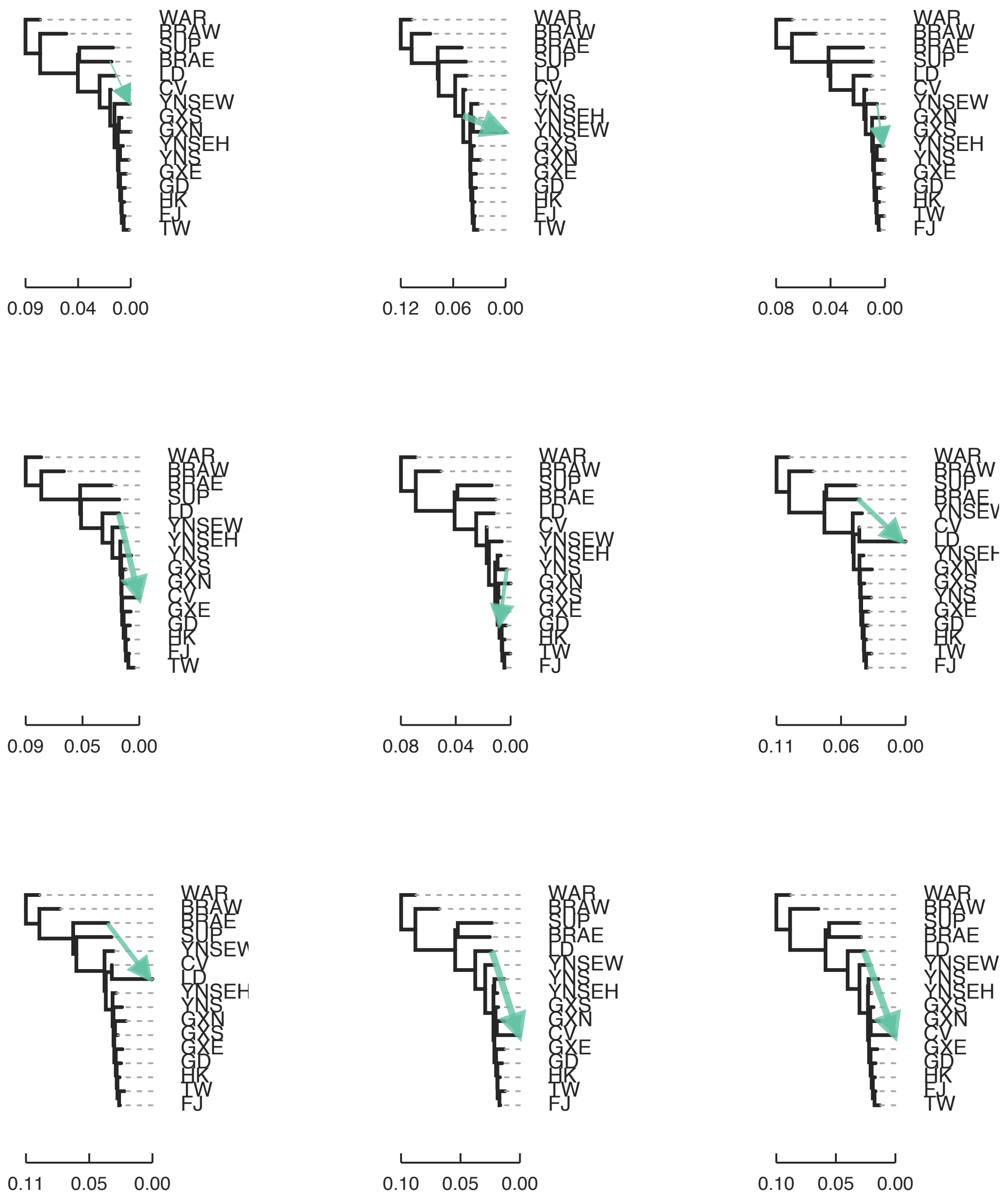

Data matrix: 136 taxa (min4); m (admixture edges) = 2
10,259 unlink SNPs (loci) sampled from 197,779 SNPs

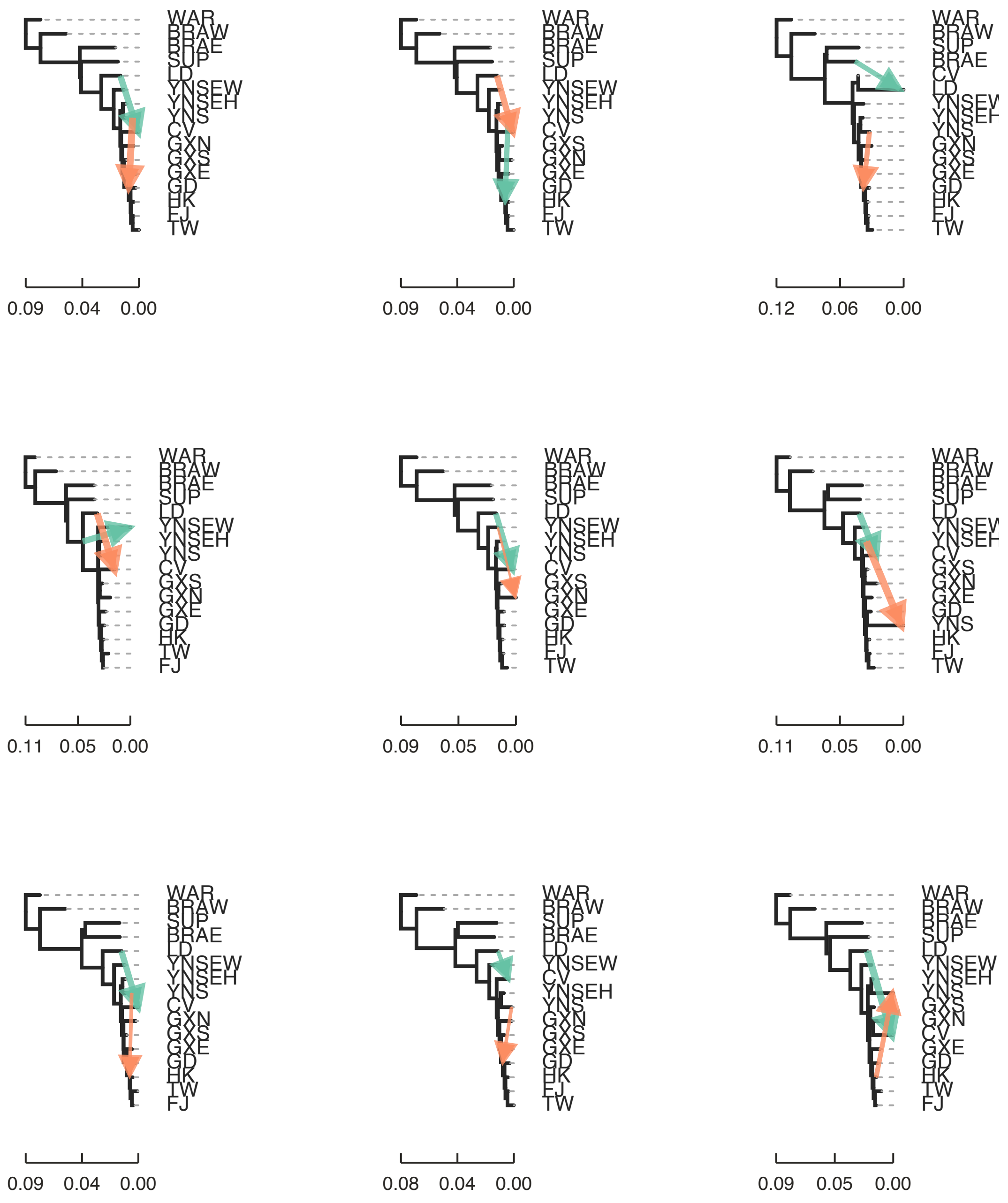

Data matrix: 136 taxa (min4); m (admixture edges) = 3
10,259 unlink SNPs (loci) sampled from 197,779 SNPs

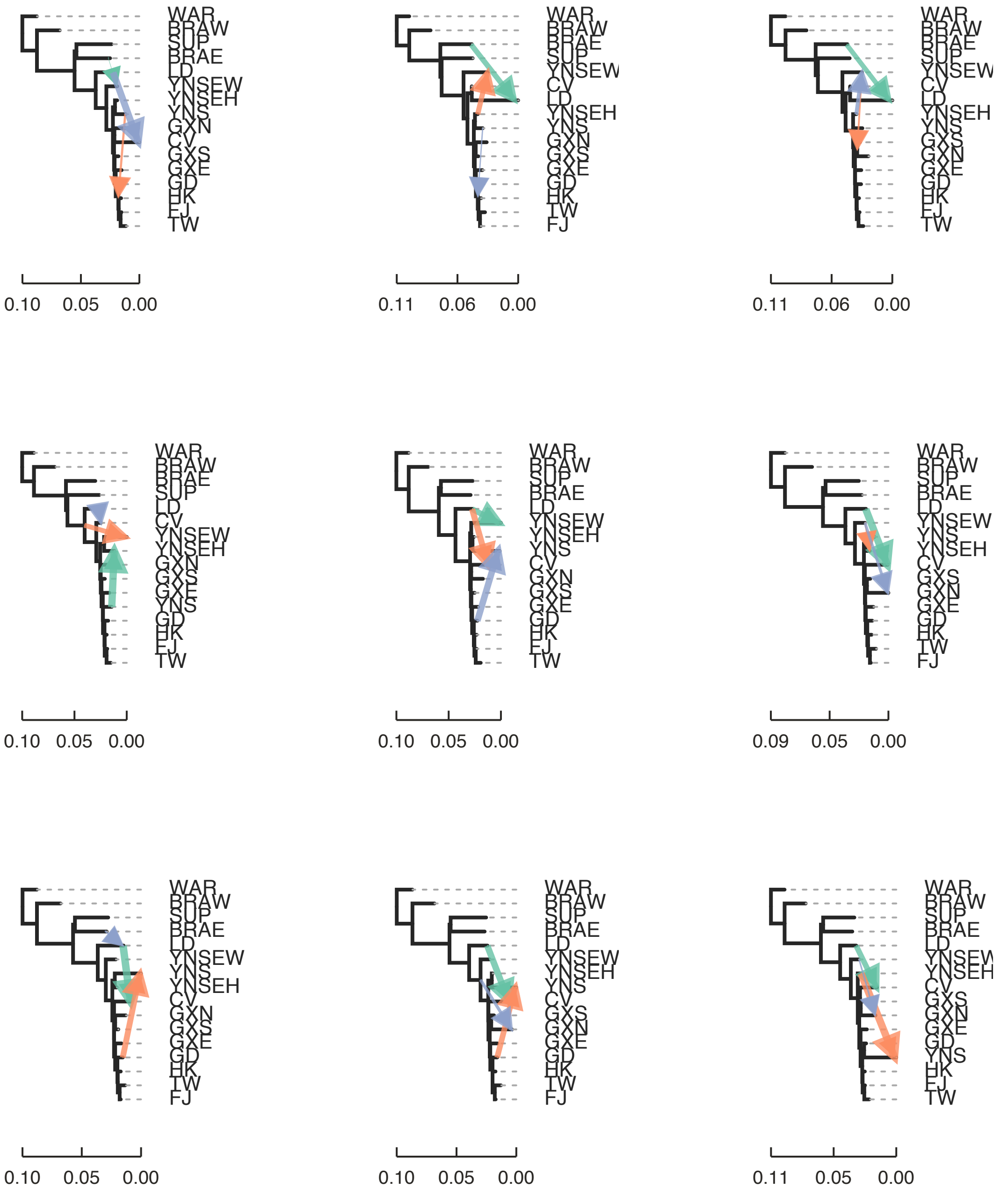

Data matrix: 136 taxa (min4); m (admixture edges) = 4
10,259 unlink SNPs (loci) sampled from 197,779 SNPs

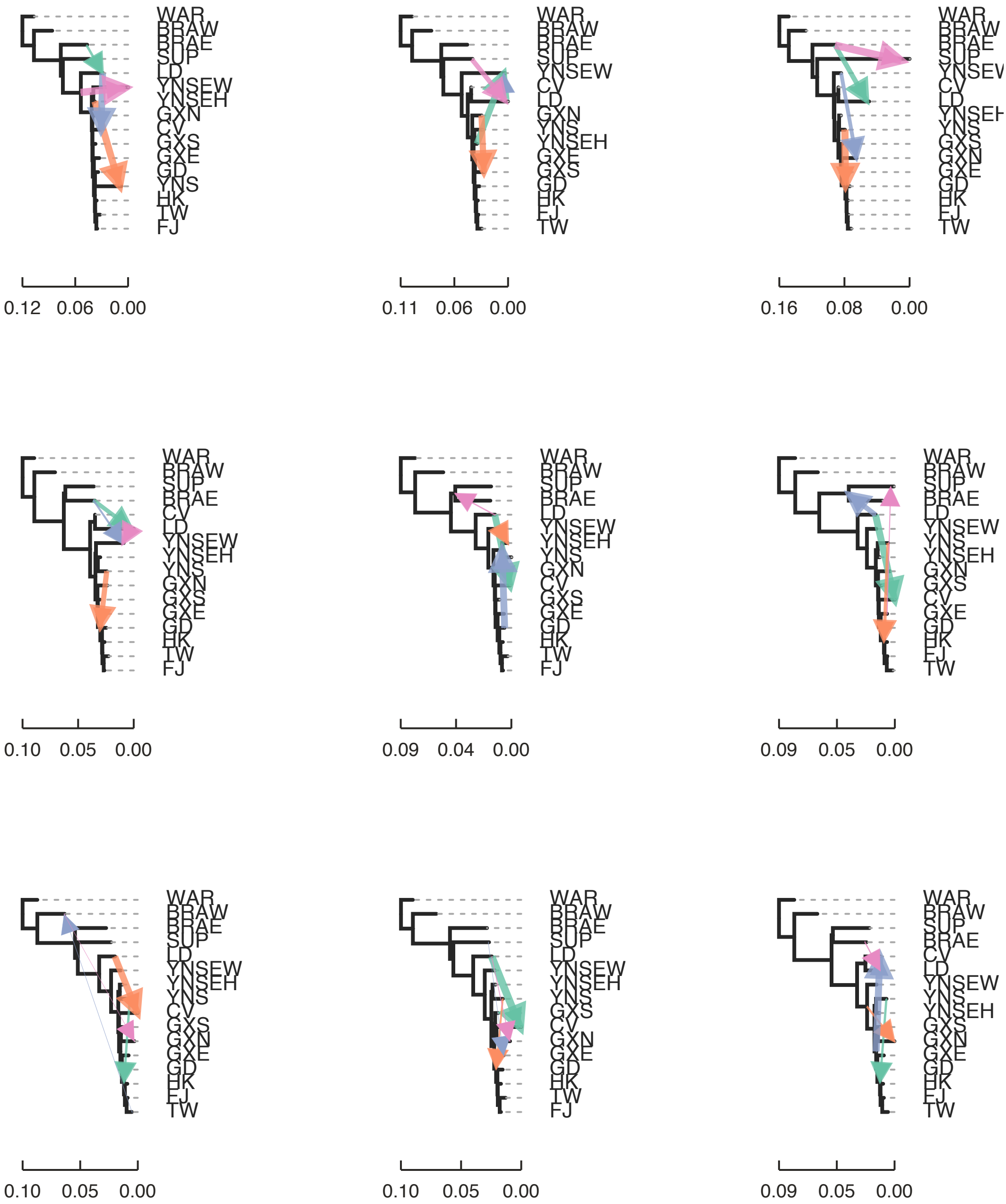

Data matrix: 136 taxa (min4); m (admixture edges) = 5
10,259 unlink SNPs (loci) sampled from 197,779 SNPs

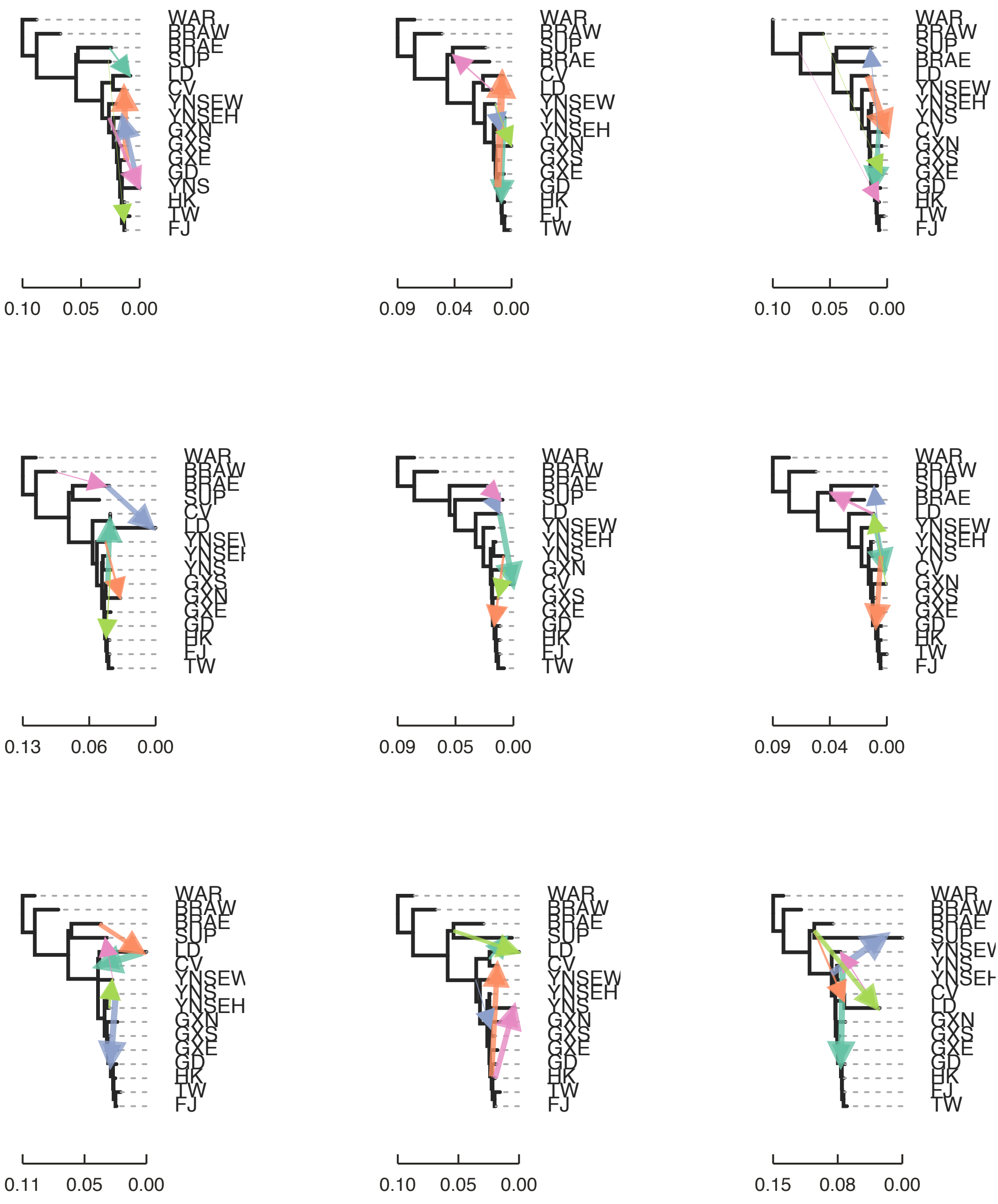

Supplement: Supplementary file 4 — Fig. S5 Results of TreeMix analyses. Fig. S6 Correlations between different floral trait measurements among three datasets. Fig. S7 Principal component analysis (PCA) of floral traits for the female‐staged individuals. Methods S1 Categorization of pollinator functional groups. Notes S1 Identification, habits, and morphology of rodent visitors in Vietnam. [file NPH-249-3137-s006.pdf]
