# Supplementary material for: Testing macroevolutionary predictions of the Grant‐Stebbins model in the origin of Aeschynanthus acuminatus
Source: New Phytol. 2026 Jan 27;249(6):3137–48. doi: 10.1111/nph.70871 (PMC12917478; doi:10.1111/nph.70871)
Supplement: Supplementary file 6 — Table S3 Shapiro–Wilk tests for normality and Levene's tests for equality of variances among floral traits. Table S4 F ST among populations. Table S5 Visitation rate, contact rate, and pollinator importance among pollinator functional groups across study sites. Table S6 Means and SD of floral trait measurements among pollinator functional groups. Table S7 Welch's ANOVA for testing floral morphology among pollinator functional groups. Table S8 Games‐Howell nonparametric post‐hoc tests for significant differences in floral traits between pollinator functional groups. Please note: Wiley is not responsible for the content or functionality of any Supporting Information supplied by the authors. Any queries (other than missing material) should be directed to the New Phytologist Central Office. [file NPH-249-3137-s003.pdf]

New Phytologist Supporting Information

Article title: Testing macroevolutionary predictions of the Grant-Stebbins model in the origin of *Aeschynanthus acuminatus*
Authors: Jing-Yi Lu, Yaowu Xing, Hong Truong Luu, Richard H Ree
Article acceptance date: 4 December 2025

Figure S2. Cross-validation errors (CV errors) and individual ancestry proportions in *ADMIXTURE* analyses.

Figure S6. Correlations between different floral trait measurements for three datasets.

Figure S7. Principal component analysis (PCA) of floral traits for the female-staged individuals.

Table S3. Shapiro–Wilk tests for normality and Levene's tests for equality of variances among floral traits.

Table S4. F_{ST} among populations of *Aeschynanthus acuminatus*.

Table S5. Visitation rate, contact rate, and pollinator importance among pollinator functional groups across study sites.

Table S6. Means and standard deviations of floral trait measurements among pollinator functional groups.

Table S7. Welch's ANOVA for testing floral morphology among pollinator functional groups.

Table S8. Games-Howell nonparametric post-hoc tests for significant differences in floral traits between pollinator functional groups.

Method S1. Categorization of pollinator functional groups.

Note S1. Identification, habits, and morphology of rodent visitors in Vietnam.

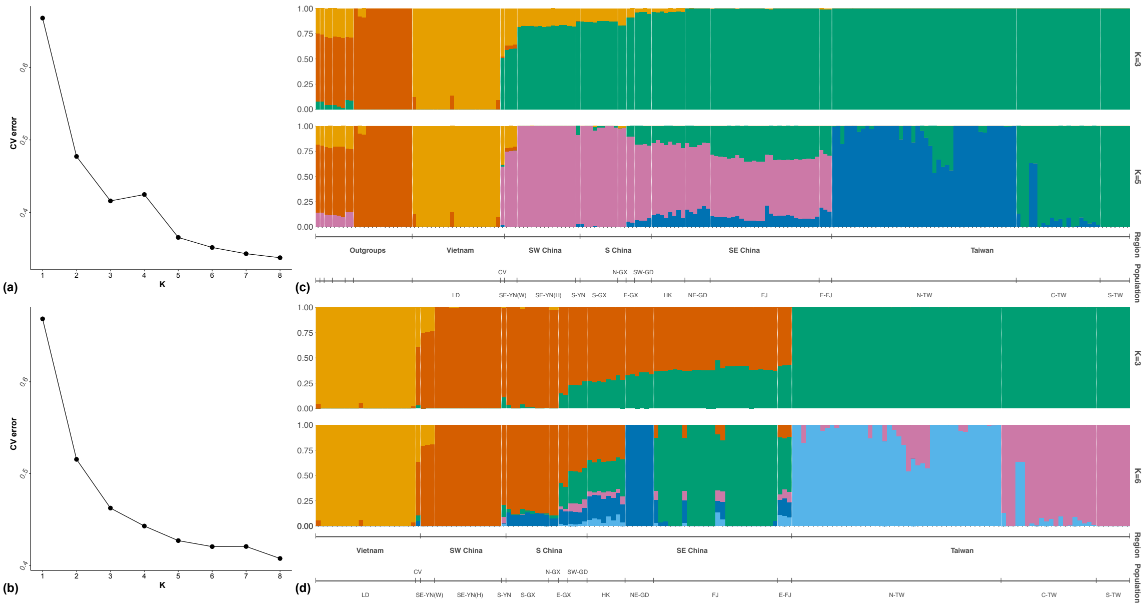

Figure S2: Cross-validation errors (CV errors) and individual ancestry proportions in *ADMIXTURE* analyses. (a)-(b) CV errors across K values for the two *ADMIXTURE* analyses. (c)-(d) individual ancestry proportions at selected K values. Each vertical bar represents an individual, partitioned into K colored segments, where each color corresponds to a distinct genetic cluster. Individuals are grouped by coarse geographic regions, then finer-scaled populations: LD = Lâm Đồng; CV = Central Vietnam; SE-YN(W) = SE Yunnan (Wenshan); SE-YN(H) = SE Yunnan (Honghe); S-YN = S Yunnan; S-GX = S Guangxi; N-GX = N Guangxi; E-GX = E Guangxi; SW-GD = SW Guangdong; HK = Hong Kong; NE-GD = NE Guangdong; FJ = Fujian; E-FJ = E Fujian; N/C/S-TW = N/C/S Taiwan. (a) and (c) full dataset with 194 samples. (b) and (d) subset dataset excluding outgroup species with 171 samples.

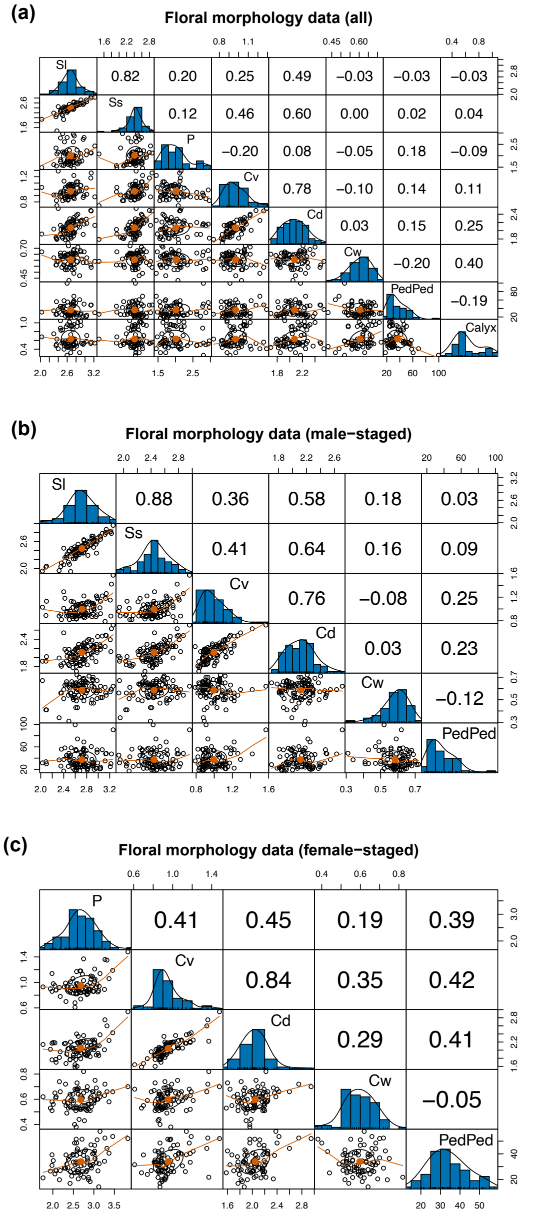

Figure S6: Correlations between different floral trait measurements for three datasets: (a) All individuals included, calyx trait not removed. (b) Male-staged individuals only, calyx trait removed. (c) Female-staged individuals only, calyx trait removed.

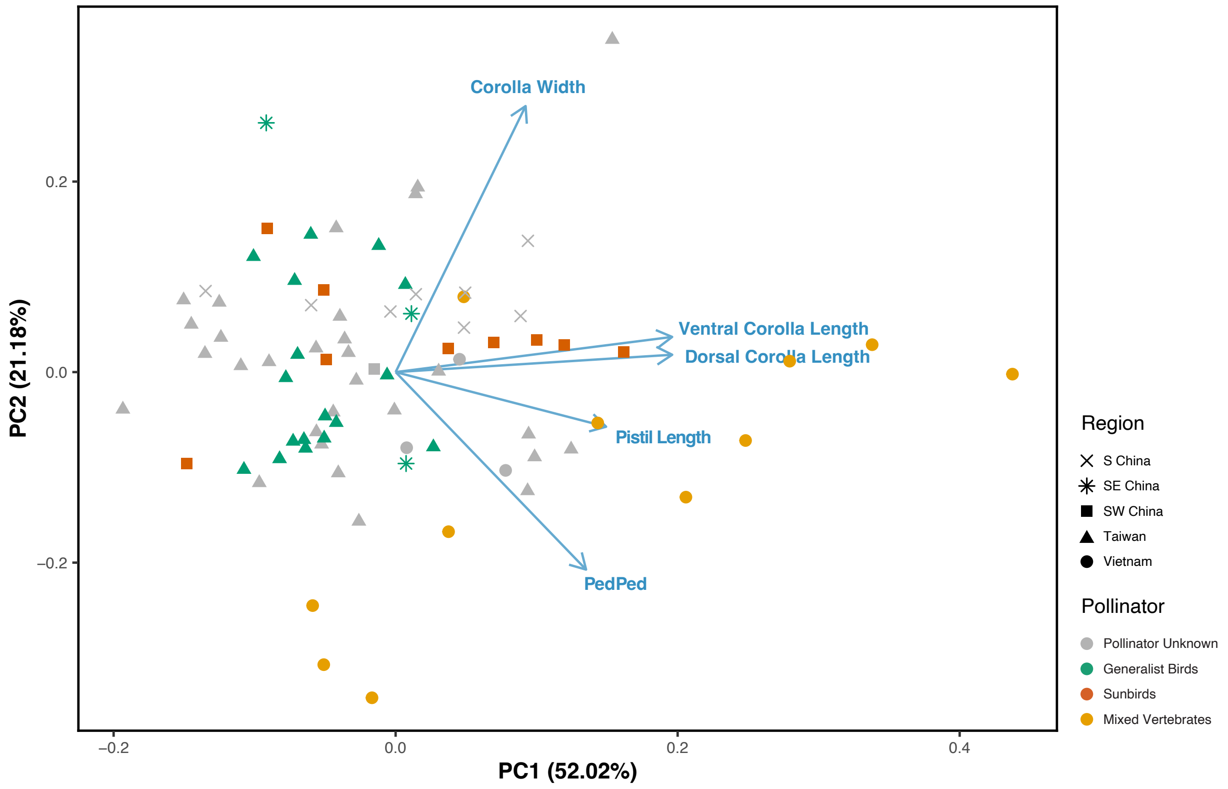

Figure S7: Principal component analysis (PCA) of floral traits for the female-staged individuals. Each point represents an individual flower of female-staged *Aeschynanthus acuminatus*. Color indicates pollinators: green = generalist birds, orange = mixed vertebrates, vermilion = sunbirds, gray = pollinator unknown. Point shapes depict geographic regions. Blue arrows indicate floral trait variable loadings.

Floral traits	Shapiro-Wolk Test			Levene's Test		
	Generalist passerines	Mixed vertebrates	Sunbirds	d.f.	F-value	Pr
Dorsal corolla	0.9766	0.3206	0.5357	(2, 34)	4.4509	0.01919*
Ventral corolla	0.3684	0.3188	0.2084	(2, 34)	6.2517	0.004874**
Corolla width	0.06458	0.8309	0.06791	(2, 34)	0.124	0.8838
Longer stamens	0.4461	0.3013	0.6172	(2, 61)	6.6354	0.002469**
Shorter stamens	0.05526	0.3994	0.5227	(2, 61)	3.5888	0.03361*
Pistils	0.6987	0.5012	0.4239	(2, 37)	1.6596	0.2041
PedPed	0.001093**	0.3158	0.4709	(2, 34)	1.0017	0.3778
PC1 (male)	0.8790	0.9749	0.5835	(2, 61)	6.3268	0.003185**
PC2 (male)	0.02478*	0.3623	0.02091*	(2, 61)	2.3132	0.2764
PC1 (female)	0.1079	0.5188	0.7136	(2, 37)	15.638	1.183×10^{-5}***
PC2 (female)	0.01799*	0.5138	0.1281	(2, 37)	3.0279	0.06055

Table S3: Shapiro–Wilk tests for normality and Levene's tests for equality of variances among floral traits.

	LD	CV*	YNSEW	YNSEH	YNS*	GXS	GXN	GXE	GD	HK	FJ	TW
LD		0.28607	0.41439	0.42292	0.4077	0.43384	0.43542	0.42774	0.44301	0.45833	0.48775	0.54508
CV*			0.33648	0.21294	N/A	0.26307	0.61826	0.41294	0.42561	0.32285	0.34693	0.43778
YNSEW				0.25736	0.32936	0.31903	0.39942	0.36349	0.38299	0.35977	0.38166	0.45035
YNSEH					0.058108	0.16175	0.19667	0.1607	0.17136	0.18732	0.23366	0.31799
YNS*						0.1966	0.6223	0.35229	0.33173	0.21586	0.24119	0.32944
GXS							0.18397	0.16702	0.19176	0.17657	0.19985	0.30546
GXN								0.43654	0.40244	0.27145	0.27106	0.36291
GXE									0.28227	0.1844	0.19915	0.29825
GD										0.15285	0.16056	0.2541
HK											0.1289	0.22843
FJ												0.18017
TW												

Table S4: F_{ST} among populations of *Aeschynanthus acuminatus*. Asterisks indicate populations with only one individual (CV and YNS). Abbreviations for each population: LD = Lâm Đồng; CV = Central Vietnam; YNSEW = SE Yunnan (Wenshan); YNSEH = SE Yunnan (Honghe); YNS = S Yunnan; GXS = S Guangxi; GXN = N Guangxi; GXE = E Guangxi; GD = Guangdong; HK = Hong Kong; FJ = Fujian; TW = Taiwan.

Region	Location	Observation hours	Pollinator functional group	Visitation rate (visits /hour)	Contact rate	Pollinator importance
Southern Vietnam	K'Long K'Lanh	912.85	Generalist passerines	0.0022	50%	0.0011 (0.143)
			Sunbirds	0.0022	100%	0.0022 (0.286)
			Rodents	0.0044	100%	0.0044 (0.571)
Southwestern China	Ladeng	219.17	Sunbirds	0.0319	42.9%	0.0137
	Shibanzhai	215	Sunbirds	0.0558	25%	0.0140
Southeastern China	Guxi	1054.33	Generalist passerines	0.0369	97.4%	0.0360
Taiwan	Fushan	482	Generalist passerines	0.0125	100%	0.0125
	Sanshuitan	332.5	Generalist passerines	0.0180	100%	0.0180
	Dinghu	280.83	Generalist passerines	0.0320	100%	0.0320

Table S5: Visitation rate, contact rate, and pollinator importance among pollinator functional groups across study sites. For the population with multiple pollinator functional groups, i.e., K'Long K'Lanh, we also calculated the relative pollinator importance (in parentheses).

Floral traits	Dataset	Generalist passerines	Mixed vertebrates	Sunbirds
Dorsal corolla	All (no missing)	1.90 ± 0.10	2.29 ± 0.21	2.09 ± 0.12
Ventral corolla	All (no missing)	0.88 ± 0.04	1.14 ± 0.15	0.97 ± 0.07
Corolla width	All (no missing)	0.61 ± 0.04	0.53 ± 0.04	0.62 ± 0.04
Longer stamens	Male (no missing)	2.66 ± 0.16	2.77 ± 0.34	2.62 ± 0.29
Shorter stamens	Male (no missing)	2.37 ± 0.15	2.52 ± 0.27	2.39 ± 0.20
Pistils	Female (no missing)	2.68 ± 0.27	3.03 ± 0.41	2.48 ± 0.33
PedPed	All (no missing)	32.6 ± 13.6	60.1 ± 20.1	36.3 ± 8.77
PC1 (male)	Male (no missing)	-1.11 ± 0.91	1.08 ± 2.23	-0.43 ± 1.23
PC2 (male)	Male (no missing)	0.60 ± 0.86	-1.21 ± 1.01	0.23 ± 0.69
PC1 (female)	Female (no missing)	-0.71 ± 0.59	2.13 ± 2.44	0.24 ± 1.53
PC2 (female)	Female (no missing)	-0.07 ± 0.96	-1.01 ± 1.31	0.30 ± 0.60

Table S6: Means and standard deviations of floral trait measurements among pollinator functional groups.

Floral traits	d.f.	F-value	Pr
Dorsal corolla	(2, 13.35)	16.971	$2.155 \times 10^{-4***}$
Ventral corolla	(2, 11.88)	18.083	$2.482 \times 10^{-4***}$
Corolla width	(2, 16.14)	10.005	0.001493^{**}
Longer stamens	(2, 25.41)	1.2592	0.301
Shorter stamens	(2, 28.03)	2.9052	0.7135
Pistils	(2, 16.63)	5.3894	0.01569^{*}
PedPed	(2, 14.30)	5.343	0.01849^{*}
PC1 (male)	(2, 27.75)	10.890	$3.228 \times 10^{-4***}$
PC2 (male)	(2, 33.73)	25.043	$2.152 \times 10^{-7***}$
PC1 (female)	(2, 12.99)	8.0990	0.005202^{**}
PC2 (female)	(2, 20.36)	4.2518	0.02864^{*}

Table S7: Welch's ANOVA for testing floral morphology among pollinator groups.

Floral traits	Mixed-Generalist passerines		Sunbirds-Generalist passerines		Sunbirds-Mixed	
	Estimate	Pr	Estimate	Pr	Estimate	Pr
Dorsal corolla	0.388 [0.141, 0.635]	0.006^{**}	0.192 [0.0844, 0.300]	$5.62 \times 10^{-4***}$	-0.196 [-0.445, 0.0534]	0.124
Ventral corolla	0.261 [0.0927, 0.429]	0.007^{**}	0.0897 [0.0897, 0.0361]	0.001^{***}	-0.171 [-0.340, -0.00270]	0.047^{*}
Corolla width	-0.0777 [-0.129, -0.0266]	0.004^{**}	[-0.0321, 0.0477]	0.878	0.0855 [0.0321, 0.139]	0.003^{**}
Longer stamens	0.108 [-0.0704, 0.287]	0.312	-0.0362 [-0.269, 0.197]	0.913	-0.144 [-0.410, 0.122]	0.381
Shorter stamens	0.146 [-0.0004, 0.293]	0.051	0.0204 [-0.149, 0.190]	0.949	-0.126 [-0.322, 0.0701]	0.267
Pistils	0.357 [-0.0029, 0.716]	0.052	-0.197 [-0.532, 0.137]	0.297	-0.554 [-0.981, 0.127]	0.01^{**}
PedPed	27.5 [4.14, 50.9]	0.024^{*}	3.75 [-6.35, 13.9]	0.633	-23.8 [-47.1, -0.499]	0.046^{*}
PC1 (male)	0.134 [0.0631, 0.205]	$1.58 \times 10^{-4***}$	0.0419 [-0.0209, 0.105]	0.229	-0.0923 [-0.177, -0.00756]	0.03^{*}
PC2 (male)	0.165 [0.108, 0.223]	$2.37 \times 10^{-8****}$	0.0341 [-0.0251, 0.0934]	0.34	-0.131 [-0.194, -0.0680]	$4.73 \times 10^{-5****}$
PC1 (female)	0.195 [0.0554, 0.335]	0.008^{**}	0.0651 [-0.0363, 0.167]	0.226	-0.130 [-0.288, 0.0283]	0.118
PC2 (female)	-0.117 [-0.242, 0.00820]	0.069	0.0247 [-0.0545, 0.104]	0.719	0.142 [0.0170, 0.267]	0.025^{*}

Table S8: Games-Howell nonparametric post-hoc tests for significant differences in floral traits between pollinator functional groups.

Methods S1. Categorization of pollinator functional groups.

In this study, we classified vertebrate pollinators into three functional groups—sunbirds (nectar specialists, Nectariniidae), generalist passerines, and rodents—following established classification in previous studies (e.g., Dellinger et al. 2019; Johnson et al. 2008).

We separated the visiting birds into two groups based mainly on their diet: nectar specialists and generalists. Sunbirds are the only known nectar specialist birds in the region. All other generalist birds were considered a single functional group. We used the term "generalist passerines" because all the generalist birds we observed were passerines (order Passeriformes).

These two groups of birds were not only separated by their diet, but they also demonstrated distinct morphology and behavior. Adapted to nectar feeding, sunbirds have evidently long and narrow beaks. The observed sunbird species in our study, Mrs. Gould's sunbird (*Aethopyga gouldiae*) and black-throated sunbirds (*Aethopyga saturata*), are similar in body sizes and beak dimensions. Thus, we expected flowers adapted to nectar specialists to have longer floral tubes with narrower corolla openings, facilitating successful contact. Sunbirds also have better hovering capabilities (not discussed in the current study). We would expect populations adapted to exclusive sunbird pollination to have longer peduncles/pedicels to reduce effective perch sites.

Here, we gathered and summarized the beak length measurements from previous research for sunbird and generalist passerine species in our study system (units in millimeters). The two sunbird species observed have relatively long beaks: Mrs. Gould's sunbird (*Aethopyga gouldiae*)—14.35 ± 0.09 ($n = 81$; Lu et al. 2023) and black-throated sunbird (*Aethopyga saturata*)—16.61 ± 0.15 ($n = 56$; Lu et al. 2023). Among generalist passerines, the most commonly observed species tend to have shorter beaks. For example, Taiwan yuhina (*Yuhina brunneiceps*)—11.7 ± 0.7 ($n = 309$; Shiu et al. 2005); Morrison's fulvetta (*Alcippe morrisonia*)—11.6 ± 1.0 ($n = 471$; Shiu et al. 2005); Huet's fulvetta (*Alcippe hueti*)—12.1 (Zhang et al. 2020). However, some generalist passerine species have beak length potentially overlapping with sunbirds, e.g., rufous-capped babbler (*Cyanoderma ruficeps*)—13.8 ± 1.0 ($n = 265$, Taiwan individuals; Shiu et al. 2005), or even larger than sunbirds, e.g., white-eared sibias (*Heterophasia auricularis*)—18.8 ± 1.2 ($n = 198$; Shiu et al. 2005), and Taiwan Scimitar-Babbler(*Pomatorhinus musicus*)—24.3 ± 1.2 ($n = 68$; Shiu et al. 2005). Nevertheless, the beak length can only partially reflect a single dimension of their mechanical fit with flowers when foraging. The narrowness of sunbird beaks is the other important aspect driving the adaptation of narrower corolla openings in other sunbird-specialized *Aeschynanthus* species. Beak width measurements are not widely available, even for species averages, so we could not provide a quantitative assessment here.

Note S1. Identification, habits, and morphology of rodent visitors of *Aeschynanthus acuminatus* in Vietnam.

The size and body proportion of visiting rodents in southern Vietnam suggest candidate species with appropriate habits and morphology for pollinating *Aeschynanthus acuminatus*. Based solely on the video recordings, we cannot confidently identify documented rodents to the species level. However, their small size and long tail, twice the body length, suggest it was either the Asiatic long-tailed climbing mouse (*Vandeleuria oleracea*, Muridae) or the Indomalayan Pencil-tailed Tree Mouse (*Chiropodomys gliroides*, Muridae). Our videos lacked sufficient detail of the fur on the tail tips and the claws on the fifth toes, which are necessary to further distinguish the two. While the two candidate species belong to different genera, they share similar habits and potentially function similarly if acting as pollinators. These two species are both nocturnal and arboreal, characterized by long tails that may aid their balance when climbing (Lunde et al. 2001). The great climbing capacity separates them from other rodents and equips them with the ability to visit flowers of epiphytes like *A. acuminatus*. Our camera traps clearly documented their agile movements up and down hanging lianas. The small size further separates them from large-bodied rodents, thereby reducing the probability of destructive impacts on flowers during visits.

References

Dellinger, A. S., M. Chartier, D. Fernández-Fernández, D. S. Penneys, M. Alvear, F. Almeda, F. A. Michelangeli, Y. Staedler, W. S. Armbruster, and J. Schönenberger. 2019. Beyond buzz-pollination–departures from an adaptive plateau lead to new pollination syndromes. *New Phytologist* **221**: 1136–1149.

Johnson, S. D. and S. W. Nicolson. 2008. Evolutionary associations between nectar properties and specificity in bird pollination systems. *Biology letters* **4**: 49–52.

Lu, W., S. Shao, L. Zu, X. Luo, and Y. Duan. 2023. Morphological diversity and altitudinal differentiation of *Aethopyga* species. *Ecology and Evolution* **13**: e10473.

Lunde, D. P. and T. S. Nguyen. 2001. *An identification guide to the rodents of Vietnam.* New York, NY, USA: American Museum of Natural History.

Shiu, H., T. Ding, J. Sheu, R. Lin, C. Koh, and P. Lee. 2005. Morphological characters of bird species in Taiwan. *Taiwania* **50**: 80.

Zhang, Q., M. Holyoak, C. Chen, Z. Liu, J. Liu, X. Che, A. Dong, C. Yang, and F. Zou. 2020. Trait-mediated filtering drives contrasting patterns of species richness and functional diversity across montane bird assemblages. *Journal of Biogeography* **47**: 301–312.
